# Plasma extracellular vesicle tau and TDP-43 as diagnostic biomarkers in FTD and ALS

**DOI:** 10.1038/s41591-024-02937-4

**Published:** 2024-06-18

**Authors:** Madhurima Chatterjee, Selcuk Özdemir, Christian Fritz, Wiebke Möbius, Luca Kleineidam, Eckhard Mandelkow, Jacek Biernat, Cem Doğdu, Oliver Peters, Nicoleta Carmen Cosma, Xiao Wang, Luisa-Sophia Schneider, Josef Priller, Eike Spruth, Andrea A. Kühn, Patricia Krause, Thomas Klockgether, Ina R. Vogt, Okka Kimmich, Annika Spottke, Daniel C. Hoffmann, Klaus Fliessbach, Carolin Miklitz, Cornelia McCormick, Patrick Weydt, Björn Falkenburger, Moritz Brandt, René Guenther, Elisabeth Dinter, Jens Wiltfang, Niels Hansen, Mathias Bähr, Inga Zerr, Agnes Flöel, Peter J. Nestor, Emrah Düzel, Wenzel Glanz, Enise Incesoy, Katharina Bürger, Daniel Janowitz, Robert Perneczky, Boris S. Rauchmann, Franziska Hopfner, Olivia Wagemann, Johannes Levin, Stefan Teipel, Ingo Kilimann, Doreen Goerss, Johannes Prudlo, Thomas Gasser, Kathrin Brockmann, David Mengel, Milan Zimmermann, Matthis Synofzik, Carlo Wilke, Judit Selma-González, Janina Turon-Sans, Miguel Angel Santos-Santos, Daniel Alcolea, Sara Rubio-Guerra, Juan Fortea, Álvaro Carbayo, Alberto Lleó, Ricardo Rojas-García, Ignacio Illán-Gala, Michael Wagner, Ingo Frommann, Sandra Roeske, Lucas Bertram, Michael T. Heneka, Frederic Brosseron, Alfredo Ramirez, Matthias Schmid, Rudi Beschorner, Annett Halle, Jochen Herms, Manuela Neumann, Nicolas R. Barthélemy, Randall J. Bateman, Patrizia Rizzu, Peter Heutink, Oriol Dols-Icardo, Günter Höglinger, Andreas Hermann, Anja Schneider

**Affiliations:** 1 German Center for Neurodegenerative Diseases (DZNE), Bonn, Germany; 2 Department of Genetics, Atatürk University, Erzurum, Turkey; 3 Department of Neurogenetics, Max Planck Institute for Multidisciplinary Sciences, Göttingen, Germany; 4 Cluster of Excellence ‘Multiscale Bioimaging: from Molecular Machines to Networks of Excitable Cells’ (MBExC), University of Göttingen, Göttingen, Germany; 5 Department of Old Age Psychiatry and Cognitive Disorders, University Hospital Bonn, University of Bonn, Bonn, Germany; 6 German Center for Neurodegenerative Diseases (DZNE), Berlin, Germany; 7 Charité – Universitätsmedizin Berlin, corporate member of Freie Universität Berlin and Humboldt-Universität zu Berlin, Institute of Psychiatry and Psychotherapy, Berlin, Germany; 8 Department of Psychiatry and Psychotherapy, Charité – Universitätsmedizin Berlin, Berlin, Germany; 9 Department of Psychiatry and Psychotherapy, Technical University of Munich School of Medicine, Munich, Germany; 10 University of Edinburgh and UK DRI, Edinburgh, UK; 11 Movement Disorder and Neuromodulation Unit, Department of Neurology, Charité – Universitätsmedizin Berlin, Berlin, Germany; 12 Department of Neurology, University of Bonn, Bonn, Germany; 13 German Center for Neurodegenerative Diseases (DZNE), Dresden, Germany; 14 Department of Neurology, University Hospital Carl Gustav Carus, Technische Universität Dresden, Dresden, Germany; 15 German Center for Neurodegenerative Diseases (DZNE), Göttingen, Germany; 16 Department of Psychiatry and Psychotherapy, University Medical Center Göttingen, University of Göttingen, Göttingen, Germany; 17 Neurosciences and Signaling Group, Institute of Biomedicine (iBiMED), Department of Medical Sciences, University of Aveiro, Aveiro, Portugal; 18 Department of Neurology, University Medical Center, Georg August University, Göttingen, Germany; 19 Cluster of Excellence Nanoscale Microscopy and Molecular Physiology of the Brain (CNMPB), University Medical Center Göttingen, Göttingen, Germany; 20 Department of Neurology, University Medicine Greifswald, Greifswald, Germany; 21 German Centre for Neurodegenerative Diseases (DZNE), Rostock/Greifswald, Germany; 22 German Center for Neurodegenerative Diseases (DZNE), Magdeburg, Germany; 23 Queensland Brain Institute, University of Queensland and Mater Public Hospital, Brisbane, Queensland Australia; 24 Institute of Cognitive Neurology and Dementia Research, Otto-von-Guericke University, Magdeburg, Germany; 25 Institute of Cognitive Neuroscience, University College London, London, UK; 26 Clinic for Neurology, University Hospital Magdeburg, Magdeburg, Germany; 27 Department of Psychiatry and Psychotherapy, University Hospital Magdeburg, Magdeburg, Germany; 28 German Center for Neurodegenerative Diseases (DZNE), Munich, Germany; 29 Institute for Stroke and Dementia Research, University Hospital, LMU Munich, Munich, Germany; 30 Department of Psychiatry and Psychotherapy, University Hospital, LMU Munich, Munich, Germany; 31 Munich Cluster for Systems Neurology (SyNergy) Munich, Munich, Germany; 32 Ageing Epidemiology Research Unit, School of Public Health, Imperial College London, London, UK; 33 Department of Neuroradiology, University Hospital LMU, Munich, Germany; 34 Sheffield Institute for Translational Neuroscience (SITraN), University of Sheffield, Sheffield, UK; 35 Department of Neurology, University Hospital of Munich, Ludwig-Maximilians-Universität (LMU) Munich, Munich, Germany; 36 Department of Psychosomatic Medicine, Rostock University Medical Center, Rostock, Germany; 37 Department of Neurology, Rostock University Medical Centre, Rostock, Germany; 38 German Center for Neurodegenerative Diseases (DZNE), Tübingen, Germany; 39 Hertie Institute for Clinical Brain Research, Department of Neurodegenerative Diseases, University of Tübingen, Tübingen, Germany; 40 Sant Pau Memory Unit, Department of Neurology, Institut de Recerca Sant Pau, Hospital de la Santa Creu i Sant Pau, Universitat Autònoma de Barcelona, Barcelona, Spain; 41 Motor Neuron Disease Clinic, Neuromuscular Diseases Unit, Institut de Recerca Sant Pau, Hospital de la Santa Creu i Sant Pau, Universitat Autònoma de Barcelona, Barcelona, Spain; 42 Centro de Investigación Biomédica en Red de Enfermedades Raras (CIBERER), Madrid, Spain; 43 Centro de Investigación Biomédica en Red de Enfermedades Neurodegenerativas (CIBERNED), Madrid, Spain; 44 Luxembourg Centre for Systems Biomedicine (LCSB), University of Luxembourg, Belvaux, Luxembourg; 45 Department of Infectious Diseases and Immunology, University of Massachussetss Medical School, North Worcester, MA USA; 46 Excellence Cluster on Cellular Stress Responses in Aging-Associated Diseases (CECAD), University of Cologne, Cologne, Germany; 47 Division of Neurogenetics and Molecular Psychiatry, Department of Psychiatry, University of Cologne, Cologne, Germany; 48 Department of Psychiatry, Glenn Biggs Institute for Alzheimer’s & Neurodegenerative Diseases, UT Health San Antonio, San Antonio, TX USA; 49 Institute for Medical Biometry, Informatics and Epidemiology, University Hospital Bonn, Bonn, Germany; 50 Department of Neuropathology, University of Tübingen, Tübingen, Germany; 51 Department of Neuropathology, University Hospital Bonn, Bonn, Germany; 52 Center for Neuropathology and Prion Research, LMU Munich, Munich, Germany; 53 Department of Neurology, Washington University School of Medicine, St. Louis, MO USA; 54 Tracy Family SILQ Center for Neurodegenerative Biology, St. Louis, MO USA; 55 Translational Neurodegeneration Section ‘Albrecht Kossel’ and Center for Transdisciplinary Neurosciences, University Medical Center Rostock, Rostock, Germany

**Keywords:** Diagnostic markers, Neurological disorders

## Abstract

Minimally invasive biomarkers are urgently needed to detect molecular pathology in frontotemporal dementia (FTD) and amyotrophic lateral sclerosis (ALS). Here, we show that plasma extracellular vesicles (EVs) contain quantifiable amounts of TDP-43 and full-length tau, which allow the quantification of 3-repeat (3R) and 4-repeat (4R) tau isoforms. Plasma EV TDP-43 levels and EV 3R/4R tau ratios were determined in a cohort of 704 patients, including 37 genetically and 31 neuropathologically proven cases. Diagnostic groups comprised patients with TDP-43 proteinopathy ALS, 4R tauopathy progressive supranuclear palsy, behavior variant FTD (bvFTD) as a group with either tau or TDP-43 pathology, and healthy controls. EV tau ratios were low in progressive supranuclear palsy and high in bvFTD with tau pathology. EV TDP-43 levels were high in ALS and in bvFTD with TDP-43 pathology. Both markers discriminated between the diagnostic groups with area under the curve values >0.9, and between TDP-43 and tau pathology in bvFTD. Both markers strongly correlated with neurodegeneration, and clinical and neuropsychological markers of disease severity. Findings were replicated in an independent validation cohort of 292 patients including 34 genetically confirmed cases. Taken together, the combination of EV TDP-43 levels and EV 3R/4R tau ratios may aid the molecular diagnosis of FTD, FTD spectrum disorders and ALS, providing a potential biomarker to monitor disease progression and target engagement in clinical trials.

## Abstract (web summary)

Plasma extracellular vesicles contain quantifiable amounts of TDP-43 and full-length tau, allowing the accurate assessment of pathology in frontotemporal dementia, frontotemporal dementia spectrum disorders and amyotrophic lateral sclerosis.

## Main

FTD encompasses different neurodegenerative disorders, including bvFTD, semantic variant primary progressive aphasia (svPPA) and nonfluent variant primary progressive aphasia. FTD, progressive supranuclear palsy (PSP), corticobasal degeneration (CBD) and ALS are part of a disease continuum with overlapping symptoms, genetics and molecular pathology^1^. Although ALS, FTD–ALS and roughly half of bvFTD cases are characterized by intracellular protein inclusions of TAR DNA-binding protein (TDP-43)^2^, PSP, CBD and approximately 40% of bvFTD cases have been linked to tau pathology at autopsy (frontotemporal lobar degeneration, FTLD-tau)^3^. Together, FTLD-tau and FTLD-TDP-43 account for nearly 90% of bvFTD cases. The microtubule-binding protein exists in six different isoforms caused by alternative splicing^4^. Based on the presence of three or four repetitive protein domains, so-called repeats, 3-repeat or 4-repeat isoforms are distinguished (3R, 4R tau). FTLD-tau can be characterized by the predominance of 3R tau aggregates (Pick’s disease) or 4R tau pathology PSP, CBD, argyrophilic grain disease or globular glial tauopathy (GGT)^5^.

So far, disease-modifying therapies are not available for FTD and ALS spectrum disorders. This is partially caused by the lack of biomarkers detecting the molecular pathology, which is a prerequisite for patient stratification in sporadic bvFTD. Currently, diagnosis of molecular pathology is only possible postmortem, with the exception of genetic cases in which a pathogenic mutation allows ante-mortem deduction of the associated molecular pathology. A diagnostic biomarker may further help in cases of diagnostic uncertainty and could facilitate early diagnosis, which is important because disease-modifying, novel therapies are expected to be more successful in the early disease stages when irreversible neuron loss is less progressed. Delayed and incorrect diagnoses have been reported for a substantial proportion of patients with ALS^6^, PSP^7^ and bvFTD^8^. Therefore, pathology-specific biomarkers are urgently needed.

Plasma glial fibrillary acidic protein/neurofilament light chain (NfL) ratios have been suggested to distinguish FTLD-tau from TDP-43 (ref. ^9^). Other studies have investigated TDP-43, phosphorylated or aggregated TDP-43 in blood or cerebrospinal fluid (CSF)^10–14^, CSF p-tau181/tau ratio^15^ or CSF peptides encoded by cryptic exons as markers of TDP-43 pathology^16^, albeit with conflicting results. CSF tau isoforms have been proposed as diagnostic markers for 3R or 4R predominant tauopathies^17^, but detection is hampered by tau fragmentation in extracellular fluids^18^ resulting in extremely low concentrations of full-length tau. We recently published a CSF assay employing immunoprecipitation followed by mass spectrometry that could overcome this obstacle^19^. However, because of the invasiveness of lumbar puncture, we turned to blood, more specifically to plasma EVs.

EVs contribute to intercellular communication or serve to clear toxic cellular content^20^. They can transport pathological tau^21–24^ and TDP-43 (ref. ^25^) species between cells and induce aggregate formation in target cells. Importantly, the presence of TDP-43 in EVs could reflect its disease-associated mislocalization from the nucleus to the cytosol, because extranuclear localization of TDP-43 is a prerequisite for its sorting into EVs.

**Extended Data Fig. 1:**
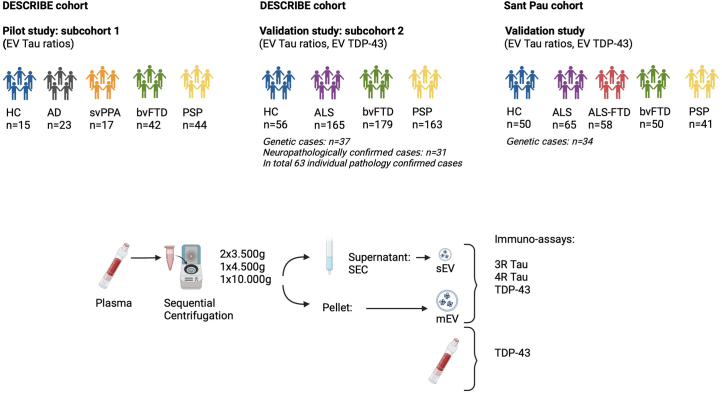
Study Design. Pilot study with samples from the DESCRIBE cohort (subcohort 1): HC, AD, svPPA, bvFTD, PSP groups, based on clinical diagnosis and supported by CSF biomarkers in AD; detection of plasma EV 3R/4R Tau. Validation study in the larger DESCRIBE subcohort 2, comprising samples of the DESCRIBE cohort with HC, ALS, bvFTD and PSP groups and 63 pathology confirmed samples: detection of plasma EV 3R/4R Tau ratios, plasma EV TDP-43 levels, and plasma TDP-43 concentrations. Validation of plasma EV Tau ratios, and plasma EV TDP-43 levels in the independent Sant Pau cohort, including HC, ALS, ALS-FTD, bvFTD, and PSP as diagnostic groups with altogether 34 genetically confirmed samples. Lower panel: Experimental flow of sEV and mEV preparation. This figure was created using BioRender.com.

Here, we show that plasma EVs contain substantial amounts of unfragmented tau. This allows the measurement of 3R and 4R tau isoform ratios, which has not been possible from blood, so far. We quantified the plasma EV 3R/4R tau ratio and TDP-43 in a large neurodegenerative disease cohort (DESCRIBE cohort) to test the hypothesis that a combination of both markers may distinguish FTLD-tau from FTLD-TDP-43 pathology. As diagnostic groups we selected bvFTD, which is largely associated with either FTLD-tau or FTLD-TDP-43 pathology, PSP based on its association with 4R tau pathology, Alzheimer disease (AD) as a secondary tauopathy with equally balanced 3R and 4R tau pathology, and svPPA and ALS as disorders with almost exclusive TDP-43 pathology, in addition to healthy controls (HC). Our cohort included 68 genetically and/or neuropathologically proven cases. Findings were validated in a second, independent cohort (Sant Pau cohort), comprising 287 participants with ALS, ALS–FTD, bvFTD, PSP and HC, including 34 genetically confirmed cases (Extended Data Fig. 1).

Intriguingly, we find that the combination of plasma EV 3R/4R tau ratio together with plasma EV TDP-43 allows the distinction of FTLD-tau from FTLD-TDP-43 in FTD and the detection of ALS-TDP-43 in ALS. Furthermore, the plasma EV 3R/4R tau ratio can serve as a biomarker to distinguish 4R tauopathy PSP from other FTD spectrum disorders and from HC.

## Results

### Detection of full-length tau in CSF and plasma EVs

We prepared medium-sized CSF and plasma EVs (mEVs) after sequential centrifugation from the 10,000*g* centrifugation pellet^26^. Small EVs (sEVs) were isolated from the 10,000*g* supernatant by size-exclusion chromatography as previously described^26^ (Supplementary Data and Supplementary Fig. 1c,d). Mass spectrometry revealed full-length tau in CSF and plasma EVs, allowing the distinction of 3R and 4R isoforms (Supplementary Data and Supplementary Fig. 2). Because venous puncture is less invasive than lumbar puncture, we decided to assess 3R and 4R tau isoform concentrations in plasma EVs, using sandwich immunoassays (Methods, Supplementary Fig. 3a–e and Supplementary Table 1).

To test whether plasma EV tau stems from brain or peripheral nerve cells^27^, thrombocytes^28^ or lymphocytes^29^, we immunoisolated anti-L1 cell adhesion molecule-positive EVs (L1CAM EVs) from plasma EV preparations. L1CAM EVs are considered brain–neuron derived, although this notion is controversial^30^. As shown in Supplementary Fig. 4, the vast majority of plasma EV tau resided in L1CAM EVs.

### Plasma EV 3R/4R tau ratio is low in PSP and high in bvFTD

We performed a pilot study on plasma EV 3R and 4R tau content in a subcohort of the DZNE multicenter DESCRIBE cohort (subcohort 1) (Extended Data Table 1 and Extended Data Fig. 1).

**Fig. 1:**
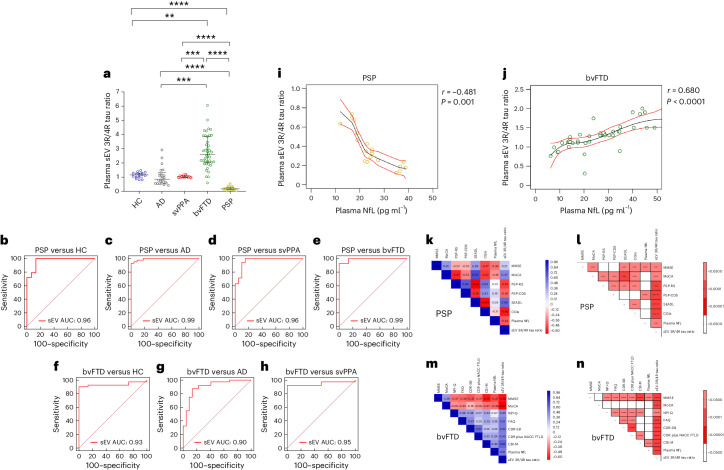
3R/4R tau ratio in plasma sEV in DESCRIBE subcohort 1. **a**, The long horizontal line represents the median and the short horizontal lines represent the IQR. Kruskal–Wallis test with Dunn’s correction for multiple comparisons. (HC versus bvFTD *P* = 0.0003, HC versus PSP *P* = 0.0000044, AD versus bvFTD *P* = 0.0003, AD versus PSP *P* = 0.0000052, svPPA versus bvFTD *P* = 0.0007, svPPA versus PSP *P* = 0.0000057, bvFTD versus PSP *P* = 0.0000019; *P < 0.05, **P < 0.001, ***P < 0.0001, ****P < 0.00001. Biologically independent samples: HC *n* = 15, AD *n* = 23, svPPA *n* = 17, bvFTD *n* = 42, PSP *n* = 44. **b**–**h**, ROC curve for sEV 3R/4R tau ratio in PSP versus HC (**b**), PSP versus AD (**c**), PSP versus svPPA (**d**), PSP versus bvFTD (**e**), bvFTD versus HC (**f**), bvFTD versus AD (**g**) and bvFTD versus svPPA (**h**). **i**,**j**, Two-tailed Spearman correlation analysis of associations and monotonic regression splines between sEV 3R/4R ratio and plasma NfL levels within PSP (**i**) and bvFTD (**j**) (*P* = 0.00009) diagnostic groups. **k**–**n**, Correlation matrix depicting results of two-tailed Spearman correlations, visualized by plotting strength of correlation (*r*) as a heat map along with *P* values (right): PSP (**k**,**l**) and bvFTD (**m**,**n**). PSP: MoCA^34^, PSP-RS^35^, PSP-CDS^36^, SEADL^37^, CGI-s^38^; PSP-SS^35^, MDS-UPDRS Part III^39^, SAS^40^ and the PSP-QoL^41^ (Supplementary Fig. 7a,b and Supplementary Table 5). bvFTD: MMSE^33^, MoCa, FAQ^42^, CDR-SB^43^, CDR plus NACC FTLD, previously termed CDR-SB FTD^44^, NPI-Q^45^ and CBI-M^46^.

The plasma EV 3R/4R tau ratio did not correlate with age, sex and disease duration (Supplementary Table 2). HC, AD and svPPA groups showed plasma sEV 3/4R tau ratios of ~1, consistent with the balanced ratios of 3R and 4R tau described in physiological conditions and in AD tau aggregates^31^ (HC median 1.16, interquartile range (IQR) [0.99–1.28]; AD median 0.91, IQR [0.57–1.25]; svPPA median 1.00, IQR [0.98–1.11]) (Fig. 1a). sEV 3R/4R tau ratios were lower in the 4R tauopathy PSP (median 0.18, IQR [0.13–0.29]; *P* < 0.0001 for all comparisons), and higher in bvFTD compared with all other groups (bvFTD median 2.59, IQR [2.02–3.87], *P* < 0.001 versus HC; *P* < 0.0001 versus all other groups). Individual bvFTD values overlapped partially with HC, svPPA and groups. Receiver operating characteristic (ROC) curve analysis revealed high diagnostic accuracies for the distinction of PSP (Fig. 1b–e) and bvFTD (Fig. 1f–h) from all other groups (AUC PSP versus HC 0.96, 95% confidence interval (CI) [0.83–0.98]; PSP versus AD 0.99, CI [0.90–1.00]; PSP versus svPPA 0.96, CI [0.90–0.98]; PSP versus bvFTD 0.99, CI [0.94–1.00]; bvFTD versus HC 0.93, CI [0.88–1.00]; bvFTD versus AD 0.90, CI [0.90–1.00]; bvFTD versus svPPA 0.95, CI [0.90–0.98]) (Supplementary Table 3).

Plasma sEV 3R/4R tau ratios correlated positively (*r* = 0.68, *P* < 0.0001) with plasma NfL levels in bvFTD, and negatively in the 4R tauopathy PSP (Fig. 1i,j (sEV *r* = −0.48, *P* = 0.001)). High plasma EV 3R/4R tau ratios in bvFTD corresponded to more severe clinical and cognitive impairment, as did low ratios in PSP, consistent with 4R tau predominance in PSP (Fig. 1k–n, Supplementary Fig. 5 and Supplementary Tables 4 and 5).

We next tested for a potential correlation of 3R/4R tau ratios between CSF and plasma sEV (Supplementary Fig. 6). However, low sample numbers and the need for larger CSF sample volumes prevent a clear conclusion on whether CSF and plasma sEV 3R and 4R tau correlate with each other (Supplementary Table 6).

**Fig. 2:**
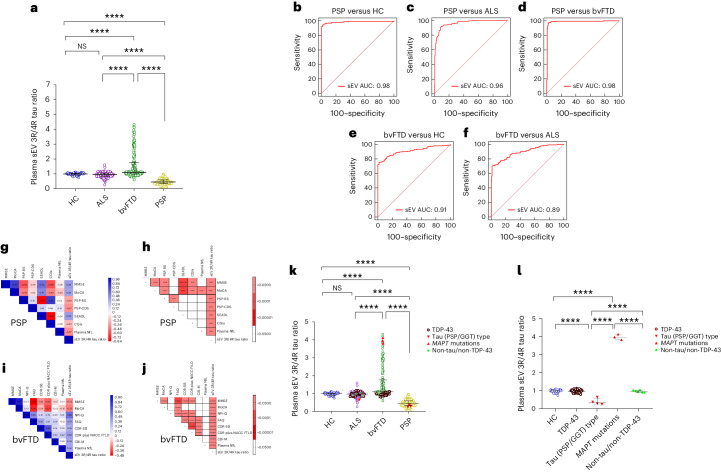
3R/4R tau ratio in plasma sEV in DESCRIBE subcohort 2. **a**, Horizontal lines indicate median and IQR. Kruskal–Wallis test with Dunn’s correction for multiple comparisons. HC versus bvFTD *P* = 0.0000057, HC versus PSP *P* = 0.000009, ALS versus bvFTD *P* = 0.0000074, ALS versus PSP *P* = 0.0000023, bvFTD versus PSP *P* = 0.0000067; ****P < 0.00001. Biologically independent samples: HC *n* = 56, ALS *n* = 165, bvFTD *n* = 179, PSP *n* = 163. **b**–**f**, ROC curve for plasma sEV 3R/4R tau ratio: PSP versus HC (**b**), PSP versus ALS (**c**), PSP versus bvFTD (**d**), bvFTD versus HC (**e**) and bvFTD versus ALS (**f**). **g**–**j**, Correlation matrix depicting the results of two-sided Spearman correlations, visualized by plotting strength of correlation (*r*) as a heat map along with *P* values (right): PSP (**g**,**h**) and bvFTD (**i**,**j**). **k**,**l**, 3R/4R tau ratio in plasma-derived sEV in genetically (*n* = 37) or autopsy-confirmed (*n* = 31) cases from DESCRIBE subcohort 2 (total number of individual cases *n* = 63, 5 of these cases had both genetic and neuropathological diagnosis). **k**, sEV 3R/4R tau ratios in the different pathology groups, stratified by clinical diagnosis. HC versus bvFTD *P* = 0.0000052, HC versus PSP *P* = 0.0000012, ALS versus bvFTD *P* = 0.0000097, ALS versus PSP *P* = 0.0000056, bvFTD versus PSP *P* = 0.0000041; ****P < 0.00001. **l**, sEV 3R/4R tau ratios of the different pathology groups, independent of clinical diagnostic group. The long horizontal line represents the median and the short horizontal lines represent the IQR. Kruskal–Wallis test with Dunn’s correction for multiple comparisons. HC versus tau (PSP/GGT)-type *P* = 0.000007, HC versus *MAPT* mutations *P* = 0.0000063, tau(PSP/GGT)-type versus *MAPT* mutations *P* = 0.000004, tau(PSP/GGT)-type versus non-tau/non-TDP-43 *P* = 0.0000078, *MAPT* mutations versus non-tau/non-TDP-43 *P* = 0.0000041; ****P < 0.00001. TDP-43 pathology group: bvFTD (*C9orf72* (*n* = 13), *GRN* (*n* = 4), *VCP* (*n* = 4), *TBK-1* (*n* = 2)); ALS (*C9orf72* (*n* = 5)); neuropathological diagnosis (FTLD-TDP (*n* = 1)); ALS-TDP (*n* = 17), ALS-FTLD-TDP (*n* = 6)). PSP/GGT-type tau pathology group: neuropathological diagnosis ((PSP-tau (*n* = 3); FTLD-tau GGT-type (*n* = 1)). bvFTD *MAPT* mutations (*MAPT P301L* (*n* = 1), *MAPT P364S* (*n* = 1), *MAPT IVS10*+*16C*>*T* (*n* = 1)). Non-tau/non-TDP-43 pathology group: ALS (*SOD-1* (*n* = 2); *FUS* (*n* = 2); *CHCHD10* (*n* = 1)); bvFTD (*CHCHD10* (*n* = 1)).

We validated our findings in additionally available samples of the DZNE DESCRIBE cohort (subcohort 2: 56 HC, 165 ALS, 179 bvFTD and 163 PSP samples). Patient demographics are given in Extended Data Table 2. ALS was chosen as a TDP-43 control group because the vast majority of ALS cases are associated with TDP-43 pathology^32^. Plasma sEV 3R/4R tau ratios were lowest in PSP (median sEV: 3R/4R tau 0.45, IQR [0.34–0.60]) and differed from all other diagnostic groups (median sEV: HC 0.99, IQR [0.91–1.03], PSP versus HC *P* < 0.0001; ALS 0.95, IQR [0.88–1.01], PSP versus ALS *P* < 0.0001; bvFTD 1.10, IQR [0.99–1.76], PSP versus bvFTD *P* < 0.0001) (Fig. 2a). ROC analysis (Fig. 2b–d), revealed high accuracy for the distinction of PSP from HC (AUC 0.98, CI [0.96–1.00]), ALS (AUC 0.96, CI [0.94–0.99]) and bvFTD (AUC 0.98, CI [0.73–1.00]) (Supplementary Table 3 (sEV) and Supplementary Fig. 7a–d and Supplementary Table 3 (mEV)).

Increased EV 3R/4R tau ratios were detected in bvFTD. Approximately 50% (54.19%) of bvFTD values were above the control group median, suggesting tau pathology in these cases (median sEV 3R/4R tau in bvFTD 2.28, IQR [1.13–2.4]; median mEV 3R/4R tau in bvFTD 1.84, IQR [1.19–2.13]; bvFTD versus all other diagnostic groups *P* < 0.0001 (median sEV); bvFTD versus all other diagnostic groups *P* < 0.0001 (median mEV)). Plasma EV 3R/4R tau ratios distinguished bvFTD from HC, ALS and PSP with high diagnostic accuracy (sEVs AUC 0.89–0.98 (Fig. 2d–f) and mEVs AUC 0.86–0.97 (Supplementary Fig. 7d,f); Supplementary Table 3). As in subcohort 1, EV tau ratios were not correlated with age, sex and disease duration (Supplementary Table 7).

### Plasma EV 3R/4R tau ratios correlate with disease severity

**Extended Data Fig. 2:**
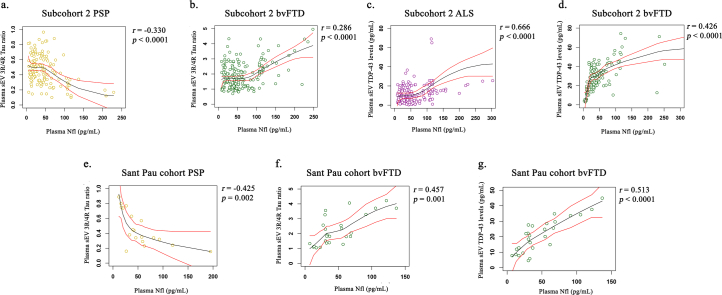
Correlation of plasma EV Tau ratio with NfL in DESCRIBE cohort 2 and Sant Pau cohort. (**a**-**d**) Two-sided Spearman correlation analysis of associations and monotonic regression splines between plasma sEV 3R/4R Tau ratios and plasma Nfl levels in (**a**) PSP (*p* = 0.000012) and (**b**) bvFTD (*p* = 0.000051) diagnostic groups. Spearman correlation analysis of associations and monotonic regression splines between plasma sEV TDP-43 levels and plasma Nfl levels in (**c**) ALS (*p* = 0.000046) and (**d**) bvFTD (*p* = 0.000063). (**e**-**g**) **Sant Pau cohort:** Spearman correlation analysis of associations and monotonic regression splines between plasma sEV 3R/4R Tau ratio and plasma Nfl levels in (**e**) PSP and (**f**) bvFTD. Spearman correlation analysis of associations and monotonic regression splines between plasma sEV TDP-43 and plasma Nfl levels in (**g**) bvFTD (*p* = 0.000085). (NfL measurements were only available for a subset of Sant Pau cases).

Similar to subcohort 1, plasma EV 3R/4R tau ratios correlated with plasma NfL in bvFTD (*r* = 0.28, *P* < 0.0001 (sEV) and *r* = 0.36, *P* = 0.002 (mEV)) and inversely in PSP (*r* = −0.33, *P* < 0.0001 (sEV) and *r* = −0.24, *P* = 0.005 (mEV)) (Extended Data Fig. 2a,b and Supplementary Fig. 7g,h).

Plasma EV 3R/4R tau ratios correlated with clinical, neurological and cognitive measures of disease severity in the PSP group, with low plasma ratios indicative of increased severity (PSP: Mini Mental State Examination (MMSE)^33^, Montreal Cognitive Assessment (MoCA)^34^, PSP rating scale (PSP-RS)^35^, PSP clinical deficits scale (PSP-CDS)^36^, Schwab and England disability scale (SEADL)^37^, Clinical Global Impression Severity Scale (CGI-s)^38^ (Fig. 2g,h and Supplementary Table 8 (sEV) and Supplementary Fig. 8i,j and Supplementary Table 8 (mEV)), PSP staging system (PSP-SS)^35^, MDS-Unified Parkinson’s Disability Rating Scale part III (MDS-UPDRS III)^39^, Starkstein Apathy Scale (SAS)^40^ and PSP quality of life scale (PSP-QoL)^41^ (Supplementary Fig. 8a,b and Supplementary Table 8).

In bvFTD, high plasma sEV 3R/4R tau ratios were associated with impaired cognition, compromised functional activities, increased symptom severity and a higher burden of behavior symptoms (MMSE, MoCA, Functional Activities Questionnaire (FAQ)^42^, Clinical Dementia Rating-Sum of Boxes (CDR-SB)^43^, Clinical Dementia Rating (CDR) plus National Alzheimer’s Coordinating Center (NACC) Behavior and Language Domains Frontotemporal Lobar Degeneration (NACC FTLD)^44^, Neuropsychiatric Inventory Questionnaire (NPI-Q)^45^, and the modified version of the Cambridge Behavior Inventory-Revised Version (CBI-M)^46^ (Fig. 2i,j and Supplementary Table 9)). Similar results were observed for mEVs (Supplementary Fig. 7k,l and Supplementary Table 9).

### Plasma EV tau ratios in pathology-confirmed cases

We stratified cases with known mutations (*n* = 37) or neuropathologically confirmed diagnoses (*n* = 31) into TDP-43, tau and non-TDP-43/non-tau pathology groups (number of individual cases *n* = 63, 5 of these had both genetic and neuropathological diagnosis) (Supplementary Tables 10 and 11). Most mutations were linked to TDP-43 pathology (18 *C9orf72*, 4 *GRN*, 4 *VCP* and 2 *TBK1*), with the exception of three *MAPT* mutations (*MAPT P301L, MAPT P364S* and *MAPT IVS10*+*16C*>*T*) in bvFTD.

Neuropathological diagnoses of nongenetic cases included 22 with TDP-43 (16 ALS-TDP, 6 FTLD-TDP) and 4 with tau pathology (3 PSP-type, 1 GGT-type). All genetic and neuropathologically confirmed cases with TDP-43 pathology were combined into a ‘TDP-43 pathology’ group (*n* = 50) and all with tau pathology into the ‘tau pathology’ group (*n* = 7). Cases with neither TDP-43 nor tau pathology (2 *SOD1*, 2 *FUS* and 2 *CHCHD10* mutation carriers) were classified as ‘non-TDP-43/non-tau pathology’ (*n* = 6).

In the TDP-43 pathology group, and in the non-TDP-43/non-tau pathology group, sEV 3R/4R tau ratios were ~1 and did not differ from the HC group (HC: median sEV 0.99, IQR [0.91–1.03]; TDP-43 group: median sEV 0.95, IQR [0.92–0.97], versus HC *P* > 0.05; non-TDP-43/non-tau group: median sEV 0.96, IQR [0.90–1.03], versus HC *P* > 0.05) (Fig. 2k,l (mEV) and Supplementary Fig. 9a,b). Importantly, all bvFTD TDP-43 pathology cases were in the lower range, comparable with HC, and ALS PSP/GGT-type 4R tau pathology cases were characterized by decreased plasma EV 3R/4R tau levels (median sEV 0.42, IQR [0.35–0.60]) compared with HC (median sEV 0.99, IQR [0.91–1.03], *P* < 0.00001), with TDP-43 pathology (median sEV 0.95, IQR [0.92–0.97], *P* < 0.00001) and with non-TDP43/non-tau pathology groups (median sEV 0.96, IQR [0.90–1.03], *P* < 0.00001). By contrast, EV 3R/4R tau ratios in *MAPT* mutation carriers were approximately three to four times higher (median sEV 3.96, IQR [3.81–4.12]) compared with HC, TDP-43 and non-TDP-43/non-tau control groups (*P* < 0.00001). Thus, EV 3R/4R tau ratios may separate FTLD-tau pathology from FTLD-TDP and detect PSP/GGT-type tau pathology.

### Plasma EVs contain TDP-43

Western blotting (Supplementary Fig. 10a) and single-molecule array (SIMOA) assay analysis confirmed the presence of TDP-43 in plasma EVs (for specificity and assay performance see Supplementary Data, Supplementary Fig. 10 and Supplementary Table 1). As illustrated in Supplementary Fig. 4, the majority of plasma EV TDP-43 stems from L1CAM-positive EVs.

### Plasma EV TDP-43 is increased in ALS and bvFTD

**Fig. 3:**
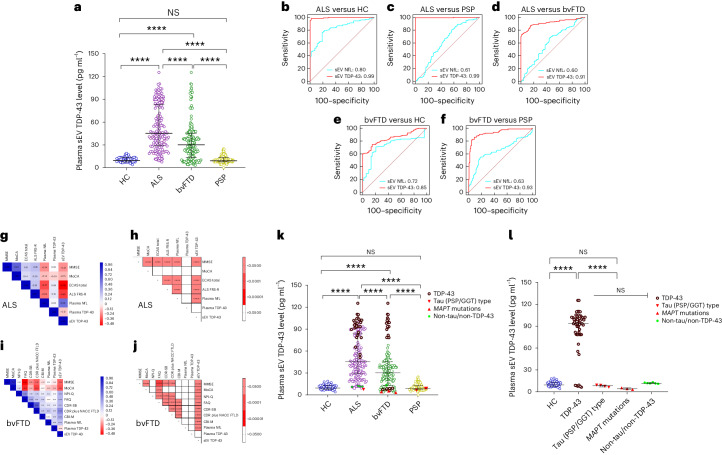
TDP-43 levels in plasma sEV in DESCRIBE subcohort 2. **a**, The long horizontal line represents the median and the short horizontal lines represent IQR. Kruskal–Wallis test with Dunn’s correction for multiple comparisons. HC versus ALS *P* = 0.000003, HC versus bvFTD *P* = 0.000006, ALS versus bvFTD *P* = 0.0000074, ALS versus PSP *P* = 0.0000028, bvFTD versus PSP *P* = 0.0000012; ****P < 0.00001. Biologically independent samples: HC *n* = 56, ALS *n* = 165, bvFTD *n* = 179 and PSP *n* = 163. **b**–**f**, ROC curve for sEV TDP-43 (red) and plasma NfL (blue): ALS versus HC (**b**), ALS versus PSP (**c**), ALS versus bvFTD (**d**), bvFTD versus HC (**e**) and bvFTD versus PSP (**f**). **g**–**j**, Correlation matrix depicting results of two-sided Spearman correlations, visualized by plotting strength of correlation (*r*) as a heat map along with *P* values (right). ALS (**g**,**h**) and bvFTD (**i**,**j**). TDP-43 in plasma sEV in genetically (*n* = 37) or autopsy-confirmed (*n* = 31) cases from the DESCRIBE subcohort 2 (total number of individual cases: *n* = 63, 5 of which had both genetic and neuropathological diagnoses). **k**, sEV TDP-43 in the different pathology groups, stratified by clinical diagnosis. HC versus ALS *P* = 0.000003, HC versus bvFTD *P* = 0.000006, ALS versus bvFTD *P* = 0.0000074, ALS versus PSP *P* = 0.0000028, bvFTD versus PSP *P* = 0.0000012; ****P < 0.00001. **l**, sEV TDP-43 concentrations in the different pathology groups, independent of clinical diagnostic group. The long horizontal line represents the median and the short horizontal lines represent the IQR. Kruskal–Wallis test with Dunn’s correction for multiple comparisons. HC versus TDP-43 *P* = 0.000008, TDP-43 versus tau (PSP/GGT)-type *P* = 0.000006, TDP-43 versus *MAPT* mutations *P* = 0.0000035, TDP-43 versus non-tau/non-TDP-43 *P* = 0.0000039; ****P < 0.00001. TDP-43 pathology group: bvFTD (*C9orf72* (*n* = 13), *GRN* (*n* = 4), *VCP* (*n* = 4), *TBK1* (*n* = 2)); ALS (*C9orf72* (*n* = 5)); neuropathological diagnosis (FTLD-TDP (*n* = 1); ALS-TDP (*n* = 17), ALS-FTLD-TDP (*n* = 6)). PSP/GGT-type tau pathology group: neuropathological diagnosis (PSP-tau (*n* = 3); FTLD-tau GGT-type (*n* = 1)). bvFTD *MAPT* mutations: *MAPT P301L* (*n* = 1), *MAPT P364S* (*n* = 1), *MAPT IVS10*+*16C*>*T* (*n* = 1). Non-tau/non-TDP-43 pathology group: ALS (*SOD-1* (*n* = 2); *FUS* (*n* = 2); *CHCHD10* (*n* = 1)); bvFTD (*CHCHD10* (*n* = 1)).

**Extended Data Fig. 3:**
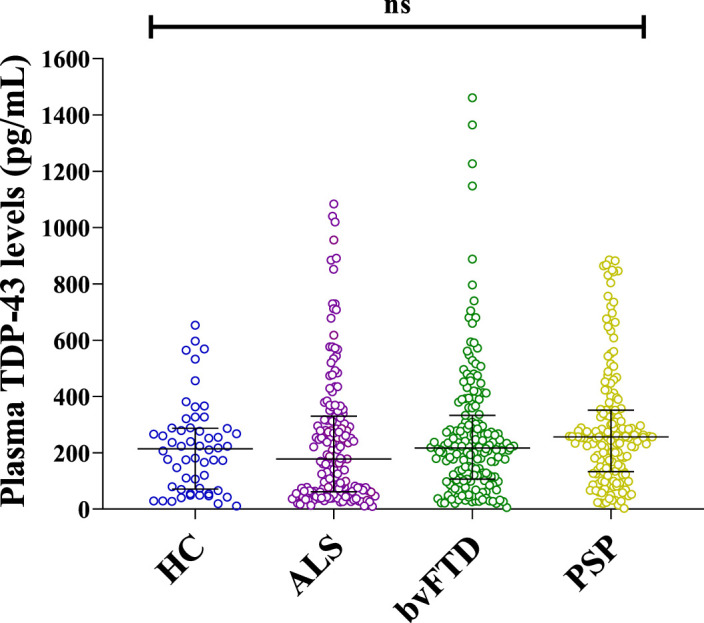
Plasma TDP-43 levels in DESCRIBE subcohort 2. (biologically independent samples: HC n = 56, ALS n = 165, bvFTD n = 179, PSP n = 163). The long horizontal line represents the median and the short horizontal lines represent the inter-quartile range (IQR) Kruskal–Wallis test with Dunn’s correction for multiple comparisons. ns: non-significant.

Plasma sEV TDP-43 levels were highest in ALS (median 45.45 pg ml^−1^, IQR [28.88−83.21]) compared with HC (9.47 pg ml^−1^, IQR [7.63−13.33], *P* < 0.00001), bvFTD (31.25 pg ml^−1^, IQR [14.45−41.09]) and PSP (9.09 pg ml^−1^, IQR [7.73−13.27], *P* < 0.00001) (Fig. 3a (sEV) and Supplementary Fig. 11a and Supplementary Table 3 (mEV)). Plasma sEV TDP-43 distinguished ALS from HC, PSP and bvFTD with AUC values of 0.99, CI [0.97–1.00]; 0.99, CI [0.98–1.00]; and 0.91, CI [0.88–0.94] (Fig. 3b–d). Similar results were obtained for plasma mEV TDP-43 concentrations and AUC values (Supplementary Fig. 11a–d). No correlation was observed with age, sex or disease duration (Supplementary Table 7). Of note, plasma TDP-43 levels did not differ between the diagnostic groups, highlighting the importance of EV analysis (Extended Data Fig. 3).

In bvFTD, plasma EV TDP-43 levels partially overlapped with HC and PSP (low levels) and the ALS group (high levels), suggesting that high levels could indicate TDP-43 pathology in bvFTD. Plasma sEV TDP-43 distinguished bvFTD from HC, PSP and ALS (AUC: bvFTD versus HC 0.85, CI [0.82–0.90]; versus PSP 0.93, CI [0.86–0.89]; versus ALS 0.91, CI [0.0.88–0.94]) (Fig. 3d–f (sEV) and Supplementary Fig. 11d–f (mEV)). Of note, plasma EV TDP-43-based AUC values exceeded plasma NfL-based AUC values (plasma NfL: ALS versus HC 0.83, CI [0.77–0.88]; versus PSP 0.62, CI [0.56–0.67]; versus bvFTD 0.61, CI [055–0.66]; bvFTD versus HC 0.73, CI [0.71–0.75]; versus PSP 0.63, CI [0.61–0.71]; *P* < 0.0001 for all comparisons) (Fig. 3b–f (sEV), Supplementary Fig. 11b–f and Supplementary Table 3 (mEV)).

### Plasma EV TDP-43 correlates with disease severity

Plasma EV TDP-43 levels were highly correlated with plasma NfL concentrations in ALS and bvFTD (ALS sEV: *r* = 0.67, *P* < 0.0001; bvFTD sEV: *r* = 0.42, *P* < 0.0001; Extended Data Fig. 2c,d (sEV) and Supplementary Fig. 11g,h (mEV)). In ALS, higher plasma EV TDP-43 levels were associated with worse cognitive performance and disease severity (MMSE, Edinburgh Cognitive and Behavioral ALS Screen total score, ALS Functional Rating Scale (ALS–FRS)) (Fig. 3g,h (sEV), Supplementary Fig. 11i,j (mEV) and Supplementary Table 12). In bvFTD, plasma EV TDP-43 concentrations correlated with cognitive impairment, impaired functional activities, increased symptom severity, more severe psychiatric and behavior symptoms (MMSE, MoCA, FAQ, CDR-SB, CDR plus NACC FTLD, NPI-Q, CBI-M) (Fig. 3i,j (sEV), Supplementary Fig. 11k,l (mEV) and Supplementary Table 9). Plasma EV TDP-43 levels correlated with CSF EV TDP-43 in the ALS group (Supplementary Fig. 12).

### Plasma EV TDP-43 levels in pathology-confirmed cases

We next compared plasma EV TDP-43 levels of confirmed TDP-43, tau or non-TDP-43/non-tau pathology cases stratified by clinical diagnosis (Fig. 3k) and independent of clinical diagnosis (Fig. 3l). In the TDP pathology cases, EV TDP-43 was increased compared with HC (median sEV: 63.95 pg ml^−1^, IQR [42.89–86.63], *P* < 0.0001), PSP/GGT-type tau (median sEV: 2.85 pg ml^−1^, IQR [2.10–3.52], *P* < 0.00001), genetic *MAPT* (median sEV: 2.86 pg ml^−1^, IQR [2.53–3.02], *P* < 0.00001) and the non-TDP-43/non-tau pathology group (median sEV: 11.35 pg ml^−1^, IQR [10.62–12.05], *P* < 0.00001) (Fig. 3i; see Supplementary Fig. 13a,b for mEV data). In bvFTD with confirmed TDP-43 pathology, plasma EV TDP-43 levels were higher compared with bvFTD with *MAPT* mutations (bvFTD with TDP-43 pathology median sEV TDP-43: 36.15 pg ml^−1^, IQR [4.52–52.65]; bvFTD with *MAPT* mutations median sEV TDP-43: 2.86 pg ml^−1^, IQR [2.53–3.02], *P* < 0.0001). EV TDP-43 levels in PSP/GGT-type tau pathology were comparable with non-TDP-43/non-tau and HC groups (*P* > 0.05) (Fig. 3l and Supplementary Fig. 13b). Surprisingly, *VCP* and *TBK1* mutation carriers showed low levels of EV TDP-43, although both mutations had been linked to TDP-43 pathology before^47,48^.

### Plasma EV tau ratio and TDP-43 aid the diagnosis of FTD and ALS

**Fig. 4:**
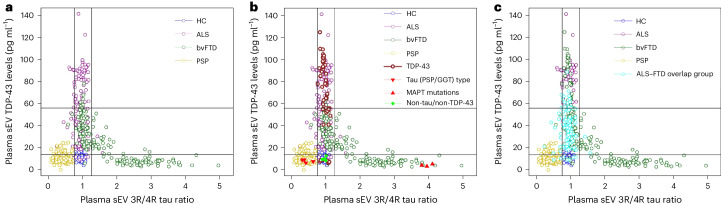
Distribution of plasma sEV 3R/4R tau ratio versus plasma EV TDP-43 levels stratified by diagnosis in DESCRIBE subcohort 2. **a**, Subcohort 2 without pathology-confirmed cases. Color codes indicate the different clinical diagnostic groups (ALS, bvFTD, PSP, HC). Cut-off values were determined by Gaussian mixture modeling. EV 3R/4R tau ratio cut-offs: 0.77 and 1.28; EV TDP-43 cut-offs: 13.87 pg ml^−1^ and 56.18 pg ml^−1^. **b**, Genetically or neuropathologically confirmed cases were also plotted. **c**, The ALS–FTD overlap group (ALS with FTD (ALS–FTD), ALS patients with cognitive impairment and ALS patients with behavioral impairment) is indicated in light blue.

**Extended Data Fig. 4:**
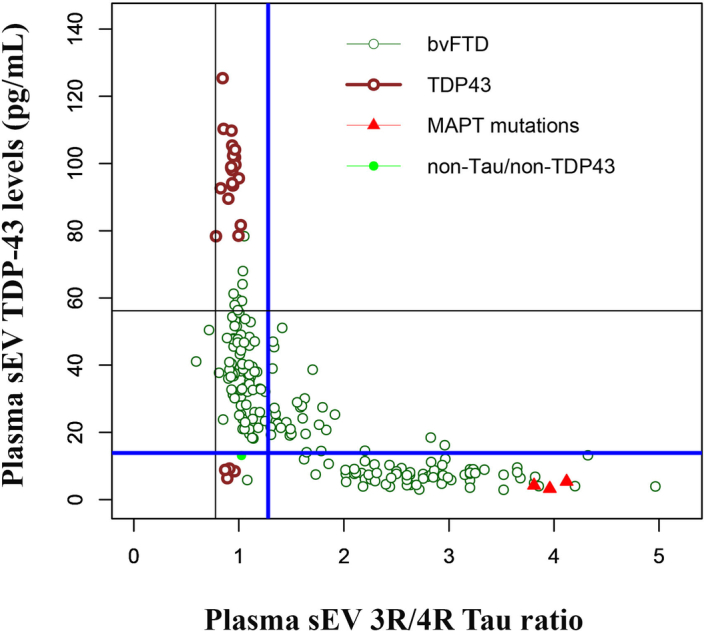
Plasma sEV 3R/4R Tau ratio versus plasma EV TDP-43 levels in bvFTD cases of DESCRIBE subcohort 2. Genetically or neuropathologically defined bvFTD cases are indicated by the different color-codes. Cut-off values as determined by Gaussian mixture modeling for subcohort 2 (Fig. 4, Supplementary Fig. 15). The two cut-offs for separation of bvFTD into putative TDP-43 and Tau pathology groups are indicated by bold blue lines (sEV 3R/4R Tau ratio cut-off: 1.27; sEV TDP-43 cut-off: 13.87 pg/ml).

In bvFTD, plasma EV TDP-43 concentrations were inversely correlated with EV 3R/4R tau ratios (sEV: *r* = −0.496, *P* < 0.0001; Supplementary Fig. 14a,b), indicating that high TDP-43 levels are associated with low tau ratios and vice versa. A plot of plasma sEV TDP-43 concentrations versus sEV 3R/4R tau ratios without genetically and neuropathologically confirmed cases revealed a clear separation of bvFTD cases into two subgroups (Fig. 4a). One of the two bvFTD subgroups, characterized by a low EV tau ratio and high EV TDP-43 levels, overlapped with ALS, whereas the other was characterized by high EV 3R/4R tau ratios but low TDP-43 levels (putative FTLD-TDP and FTLD-tau groups) (Fig. 4a (subcohort 2) and Extended Data Fig. 4 (bvFTD cases only)). PSP and HC groups formed separate clusters.

We next added pathology-confirmed cases to the graph (Fig. 4b). PSP/GGT-type tau pathology cases formed a cluster characterized by low TDP-43 and 3R/4R tau ratios. The HC group and all non-TDP-43/non-tau cases grouped together, consistent with the absence of TDP-43 and tau pathology. TDP-43-confirmed pathology cases were found in the cluster of ALS and TDP-43 high bvFTD cases. By contrast, bvFTD cases with confirmed *MAPT* pathology fell into the bvFTD group with high sEV tau ratios.

We applied a mixture modeling approach to sEV 3R/4R tau and sEV TDP-43 data to obtain cut-off values of 0.77 and 1.27 for 3R/4R tau, and 13.87 pg ml^−1^ and 56.18 pg ml^−1^ for TDP-43 (Fig. 4, Supplementary Data and Supplementary Fig. 15).

Low plasma sEV 3R/4R tau ratios (<0.77) discriminated clinical PSP cases form all other individuals in subcohort 2 (sensitivity: 93.25%, CI [88.25–96.58%]; specificity: 95.25%, CI [92.68–97.12%]) as well as PSP/GGT-type tau pathology cases from other pathology-confirmed cases (sensitivity: 100%, CI [39.76–100%]; specificity: 100%, CI [93.94–100%]). High plasma sEV 3R/4R tau ratios (>1.27) were found in 38.55% of clinical bvFTD cases. All *MAPT* mutation carriers, but no other patients with confirmed pathology, fell into the high plasma sEV tau ratio category (sensitivity: 100%, CI [29.24–100%]; specificity: 100%, CI [94.04–100%]). Importantly, all but one of the remaining clinical bvFTD patients (61.45%) showed tau ratios below the upper cut-off (<1.27) but elevated TDP-43 levels (>13.87 pg ml^−1^), suggesting that sEV measurements can distinguish two separate subgroups among bvFTD patients.

TDP-43 pathology cases mapped to the TDP-43 high bvFTD (putative FTLD-TDP) and the ALS group, and were detected among all individuals with a genetically or neuropathologically proven diagnosis with a sensitivity of 88.00%, CI [76.13–95.67%] and a specificity of 100%, CI [75.29–100%] using the cut-off for at least mildly increased TDP-43 levels (>13.87 pg ml^−1^) (Fig. 4b).

ALS cases with symptoms overlapping with bvFTD showed elevated plasma sEV TDP-43 levels (Fig. 4c).

Together, our data suggest that a combination of plasma EV TDP-43 and 3R/4R tau may distinguish FTLD-tau from FTLD-TDP.

### Sant Pau validation cohort

We validated our findings in samples from the independent Sant Pau cohort^49^ (ALS (*n* = 65), ALS–FTD (*n* = 58), bvFTD (*n* = 50), FTD mutation carriers (*n* = 23), PSP (*n* = 41) and HC (*n* = 50); see Extended Data Table 3 for patient demographics).

### Plasma EV tau ratios are high in bvFTD and low in PSP

**Fig. 5:**
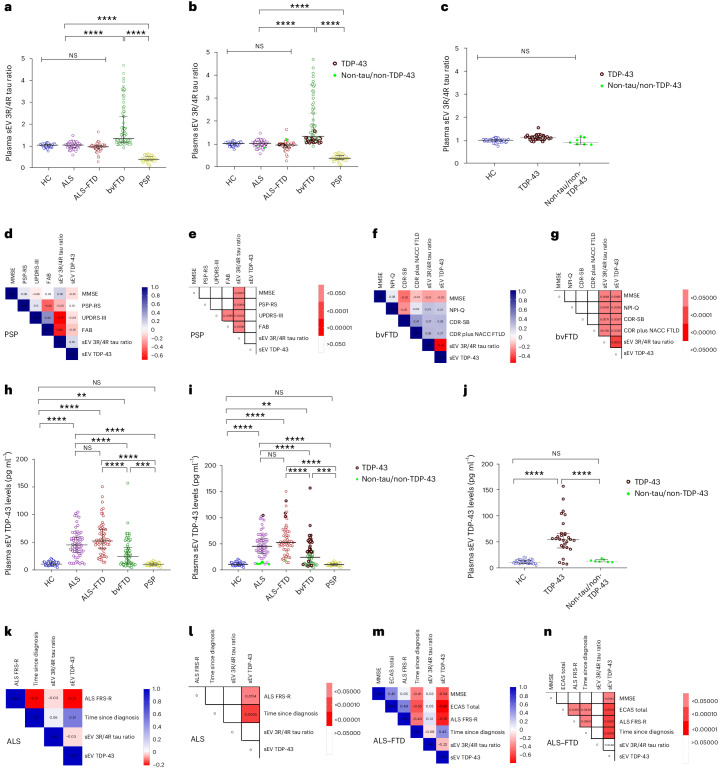
3R/4R tau ratio in plasma sEVs in the Sant Pau cohort. **a**, Stratified by clinical diagnosis. Horizontal lines indicate the median and IQR. Kruskal–Wallis test with Dunn’s correction for multiple comparisons. HC versus bvFTD *P* = 0.000009, HC versus PSP *P* = 0.000007, ALS versus bvFTD *P* = 0.0000091, ALS versus PSP *P* = 0.0000016, ALS–FTD versus bvFTD *P* = 0.0000074, ALS–FTD versus PSP *P* = 0.0000041, bvFTD versus PSP *P* = 0.000006; ****P < 0.00001. Biologically independent samples: HC *n* = 50, ALS *n* = 65, ALS–FTD *n* = 58, bvFTD *n* = 50 (+23 mutation carriers), PSP *n* = 41. **b**,**c**, sEV 3R/4R tau ratios in plasma-derived sEV in genetic cases from Sant Pau cohort (*n* = 34 genetic cases) stratified by clinical diagnosis (**b**), HC versus bvFTD *P* = 0.000009, HC versus PSP *P* = 0.000007, ALS versus bvFTD *P* = 0.0000091, ALS versus PSP *P* = 0.0000016, ALS–FTD versus bvFTD *P* = 0.0000074, ALS–FTD versus PSP *P* = 0.0000041, bvFTD versus PSP *P* = 0.000006; ****P < 0.00001; and stratified by associated molecular pathology and independent from clinical diagnosis (**c**). The long horizontal line represents the median and the short horizontal lines represent the IQR. Kruskal–Wallis test with Dunn’s correction for multiple comparisons. HC versus TDP-43 *P* = 0.758, HC versus non-tau/non-TDP-43 *P* = 0.632, TDP-43 versus non-tau/non-TDP-43 *P* = 0.425; NS, not significant. TDP-43 pathology group: bvFTD (*C9orf72* (*n* = 12), *GRN* (*n* = 6), *TARDP* (*n* = 1), *VCP* (*n* = 1), *TBK-1* (*n* = 3)); ALS (*C9orf72* (*n* = 3)); ALS–FTD (*C9orf72* (n = 1)). Non-tau/non-TDP-43 pathology group: ALS (*SOD-1* (*n* = 3)); FUS (*n* = 3); ALS–FTD (SOD-1 (*n* = 1)). **d**,**e**, Correlation matrix depicting results of two-sided Spearman correlations in the PSP group, visualized by plotting strength of correlation (*r*) as a heat map (**d**) along with *P* values (**e**). **f**,**g**, Correlation matrix depicting results of two-sided Spearman correlations in the bvFTD group, visualized by plotting strength of correlation (*r*) as a heat map (**f**) along with *P* values (**g**). **h**, TDP-43 levels in plasma sEV of the Sant Pau cohort stratified by clinical diagnosis. The long horizontal line represents the median and the short horizontal lines represent the IQR. Kruskal–Wallis test with Dunn’s correction for multiple comparisons. HC versus ALS *P* = 0.0000075, HC versus ALS–FTD *P* = 0.0000046, ALS versus bvFTD *P* = 0.00074, HC versus PSP *P* = 0.578, ALS versus bvFTD *P* = 0.000009, ALS versus PSP *P* = 0.000007, ALS–FTD versus bvFTD *P* = 0.0000043, ALS–FTD versus PSP *P* = 0.0000055, bvFTD versus PSP *P* = 0.0005; *P < 0.05, **P < 0.001, ***P < 0.0001, ****P < 0.00001. Biologically independent samples: HC *n* = 50, ALS *n* = 65, ALS–FTD *n* = 58, bvFTD *n* = 50 (+23 mutations), PSP *n* = 41. **i**,**j**, Plasma sEV TDP-43 concentrations in genetic cases of the Sant Pau cohort (*n* = 34 genetic cases) stratified by clinical diagnosis (**i**), HC versus ALS *P* = 0.0000075, HC versus ALS–FTD *P* = 0.0000046, ALS versus bvFTD *P* = 0.00074, HC versus PSP *P* = 0.578, ALS versus bvFTD *P* = 0.000009, ALS versus PSP *P* = 0.000007, ALS–FTD versus bvFTD *P* = 0.0000043, ALS–FTD versus PSP *P* = 0.0000055, bvFTD versus PSP *P* = 0.0005; and stratified by molecular pathology, independent of clinical diagnosis (**j**). The long horizontal line represents the median and the short horizontal lines represent the IQR. Kruskal–Wallis test with Dunn’s correction for multiple comparisons. HC versus TDP-43 *P* = 0.0000063, HC versus non-tau/non-TDP-43 *P* = 0.541, TDP-43 versus non-tau/non-TDP-43 *P* = 0.0000051; ****P < 0.00001. TDP-43 pathology group: bvFTD (*C9orf72* (*n* = 12), *GRN* (*n* = 6), *TARDP* (*n* = 1), *VCP* (*n* = 1), *TBK-1* (*n* = 3)); ALS (*C9orf72* (*n* = 3)); ALS–FTD *C9orf72* (*n* = 1). Non-tau/non-TDP-43 pathology group: ALS (*SOD-1* (*n* = 3); FUS (*n* = 3)); ALS–FTD (SOD-1 (*n* = 1)). **k**,**i**, Correlation matrix depicting results of two-sided Spearman correlations in the ALS group, visualized by plotting strength of correlation (*r*) as a heat map (**k**) along with *P* values (**i**). ALS-FRS (sEV: *r* = −0.212), *P* = 0.015 and time since diagnosis/disease duration (sEV: *r* = 0.514, *P* = 0.0004). **n**,**m**, Correlation matrix depicting results of two-sided Spearman correlations in the ALS−FTD group, visualized by plotting strength of correlation (*r*) as a heat map (**m**) along with *P* values (**n**). ALS-FRS (sEV: *r* = −0.702), *P* = 0.0002 and time since diagnosis/disease duration (sEV: *r* = 0.445, *P* = 0.0005); MMSE (sEV: *r* = −0.535, *P* = 0.018).

**Extended Data Fig. 5:**
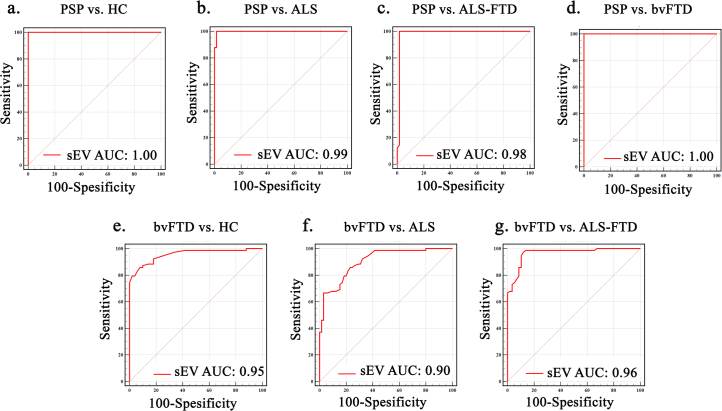
Sant Pau cohort, Receiver Operating Characteristic (ROC) curves for plasma sEV 3R/4R Tau ratios. (**a**) PSP vs. HC (**b**) PSP vs. ALS (**c**) PSP vs. ALS-FTD (**d**) PSP vs. bvFTD (**e**) bvFTD vs. HC (**f**) bvFTD vs. ALS (**g**) bvFTD vs. ALS-FTD.

Similar to our findings from DESCRIBE, tau ratios were lowest in PSP (median sEV 3R/4R tau ratio 0.38, IQR [0.33–0.50]), compared with all other groups (median sEV HC 1.02, IQR [0.96–1.06], PSP versus HC *P* < 0.00001; ALS 1.02, IQR [0.92–1.11], PSP versus ALS *P* < 0.00001; ALS–FTD 0.95, IQR [0.84–1.00], PSP versus ALS–FTD *P* < 0.00001; bvFTD 1.34, IQR [1.17–2.34], PSP versus bvFTD *P* < 0.00001) and highest in bvFTD, median sEV bvFTD versus all other diagnostic groups *P* < 0.00001) (Fig. 5a and Extended Data Table 3 (sEV), and Supplementary Fig. 16a and Extended Data Table 3 (mEV)). No correlations of EV tau ratios with age, sex and disease duration were found (Supplementary Table 13). Approximately half of the bvFTD samples were characterized by high EV tau ratios, indicating tau pathology, whereas the other half were in the range observed for HC, ALS and ALS–FTD. We confirmed the feasibility of using plasma EV tau ratio as a diagnostic marker for different tauopathies by ROC analysis (sEV 3R/4R tau ratio: PSP versus HC (AUC 1.00, CI [0.960–1.000]), PSP versus ALS (AUC 0.99, CI [0.962–1.000]), PSP versus ALS–FTD (AUC 0.98, CI [0.960–1.000]) and PSP versus bvFTD (AUC 1.00, CI [0.969–1.000]); bvFTD versus HC (AUC 0.95, CI [0.905–0.985]) and bvFTD versus ALS (AUC 0.90, CI [0.845–0.948])) (Extended Data Fig. 5a–g and Supplementary Table 14 (sEV), and Supplementary Fig. 16b–h and Supplementary Table 14 (mEV)).

Sant Pau cohort samples included 34 genetically confirmed cases, 27 with TDP-43 (*GRN* *n* = 6, *C9orf72* *n* = 16, *TARDBP* *n* = 1, *VCP* *n* = 1 and *TBK1* *n* = 3) and 7 with neither TDP-43 nor tau pathology (*SOD1 n* = 4 and FUS *n* = 3) (Supplementary Table 15). Consistent with the absence of tau pathology, plasma EV 3R/4R tau ratios of all genetically confirmed cases were in the range of HC, ALS and ALS–FTD (Fig. 5b,c and Supplementary Fig. 22a,b) (HC: median sEV 1.02, IQR [0.96–1.06]; TDP-43 pathology group: median sEV 1.15, IQR [1.07–1.23], versus HC *P* > 0.9999; non-TDP-43/non-tau group: median sEV 0.92, IQR [0.84–1.15], versus HC *P* > 0.9999).

### Plasma EV tau ratios correlate with disease severity

Plasma EV 3R/4R tau ratios correlated with plasma NfL and clinical measures of disease severity in bvFTD and inversely in PSP (Fig. 5d–g (sEV), and Supplementary Fig. 16k,n and Supplementary Tables 16 and 17 (mEV)).

### High plasma EV TDP-43 in ALS, ALS–FTD and a subset of bvFTD

As in DESCRIBE subcohort 2, plasma EV TDP-43 levels were increased in patients with ALS (median sEV TDP-43: 45.60 pg ml^−1^, IQR [31.55–64.45]) compared with HC (median sEV TDP-43: 10.41 pg ml^−1^, IQR [8.50–14.65], *P* < 0.00001), in ALS–FTD (median sEV TDP-43: 52.40 pg ml^−1^, IQR [39.18–73.43], *P* < 0.00001 compared with HC) and in bvFTD (median sEV TDP-43: 24.15 pg ml^−1^, IQR [11.13–40.55], *P* < 0.00001 compared with HC) (Fig. 5h (sEV), and Supplementary Fig. 19a and Extended Data Table 3 (mEV)). PSP EV TDP-43 levels were comparable with HC (median sEV TDP-43: 10.20 pg ml^−1^, IQR [8.30–12.35], *P* > 0.9999). Plasma sEV TDP-43 distinguished ALS from HC, PSP and bvFTD with AUC values of 0.94, CI [0.892–0.981], 0.96, CI [0.910–0.991] and 0.76, CI [0.687–0.832] (Supplementary Table 14), and ALS–FTD from HC, PSP and bvFTD groups (AUC 0.98, CI [0.946–0.999], 0.99, CI [0.955–1.000] and 0.82, CI [0.745–0.881]) (Supplementary Fig. 19a–g (sEV), Supplementary Fig. 18b–h) (mEV) and Supplementary Table 14).

Genetic cases linked to TDP-43 pathology were characterized by high EV TDP-43 levels (median sEV TDP-43: 55.0 pg ml^−1^, IQR [35.0–66.4]), with the exception of *VCP* and *TBK1* mutations similar to what we observed in the DESCRIBE cohort. Genetically confirmed cases, neither linked to TDP-43 nor tau, displayed low plasma EV TDP-43 levels (median sEV TDP-43: 12.7 pg ml^−1^, IQR [11.3–15.7]), comparable to HC and PSP (HC median sEV TDP-43: 10.41 pg ml^−1^, IQR [8.50–14.65]; PSP median sEV TDP-43: 10.20 pg ml^−1^, IQR [8.30–12.35]) (Fig. 5i,j and Supplementary Fig. 19a,b).

**Extended Data Fig. 6:**
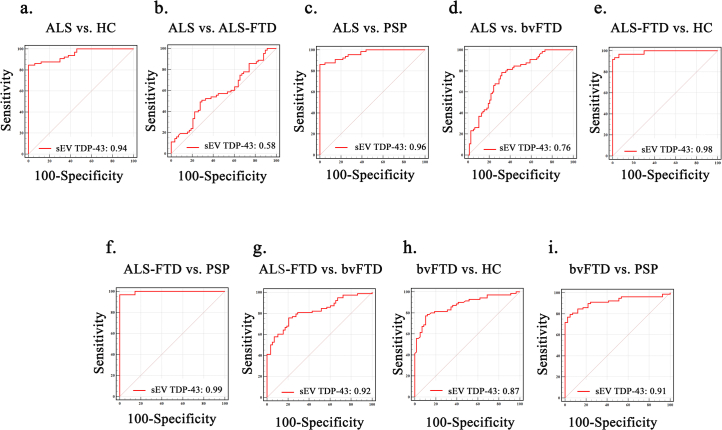
Receiver Operating Characteristic (ROC) curve with AUC values for plasma sEV TDP-43 in Sant Pau cohort. (**a**) ALS vs. HC (**b**) ALS vs. ALS-FTD (**c**) ALS vs. PSP (**d**) ALS vs. bvFTD (**e**) ALS-FTD vs. HC (**f**) ALS-FTD vs. PSP (**g**) ALS-FTD vs. bvFTD (**h**) bvFTD vs. HC (**i**) bvFTD vs. PSP.

**Extended Data Fig. 7:**
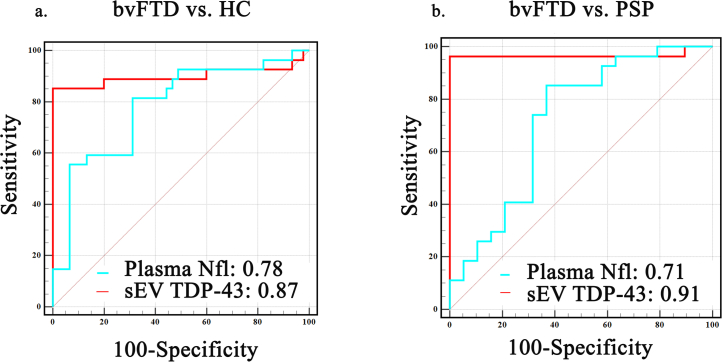
Receiver Operating Characteristic (ROC) curve with AUC values for plasma sEV TDP-43 (red) and plasma NfL (blue) in the Sant Pau cohort. (**a**) bvFTD vs. HC and (**b**) bvFTD vs. PSP. Plasma NfL levels were not available for ALS and ALS-FTD groups.

Plasma EV TDP-43 discriminated bvFTD cases from HC, PSP, ALS and ALS–FTD (AUC sEV: bvFTD versus HC 0.87, CI [0.803–0.926]; versus PSP 0.91, CI [0.851–0.959]; versus ALS 0.76, CI [0.687–0.832]; versus ALS–FTD 0.82, CI [0.745–0.881]) (Extended Data Fig. 6i and Supplementary Table 14 (sEV), and Supplementary Fig. 18h–j and Supplementary Table 14 (mEV)). Comparable with our results from DESCRIBE, plasma EV TDP-43-based AUCs performed superior to NfL for bvFTD versus HC and PSP (plasma NfL AUCs: bvFTD versus HC 0.78, CI [0.734–0.842]; versus PSP 0.71, CI [0.689–0.789]; *P* < 0.0001 for all AUC comparisons) (Extended Data Fig. 7a,b (sEV), and Supplementary Fig. 23i,j and Supplementary Table 14 (mEV)). NfL measurements were not available for ALS and ALS–FTD in the Sant Pau cohort.

### Plasma EV TDP-43 correlates with disease severity

Plasma EV TDP-43 levels correlated highly with plasma NfL concentrations in bvFTD (sEV: *r* = 0.513, *P* < 0.0001; mEV: *r* = 0.465, *P* < 0.0001) (Extended Data Fig. 2e–g (sEV) and Supplementary Fig. 20k (mEV)).

In ALS and ALS–FTD, higher plasma EV TDP-43 levels were associated with increased disease severity (ALS-FRS, time since diagnosis, MMSE; Fig. 5k–n (sEV), Supplementary Fig. 18l–o and Supplementary Tables 18 and 19a (mEV)).

In bvFTD, plasma EV TDP-43 concentrations correlated with cognitive impairment, increased symptom severity and more severe psychiatric symptoms (Fig. 5f,g (sEV), Supplementary Fig. 23p,r (mEV) and Supplementary Table 17).

### Determination of cut-off levels

**Fig. 6:**
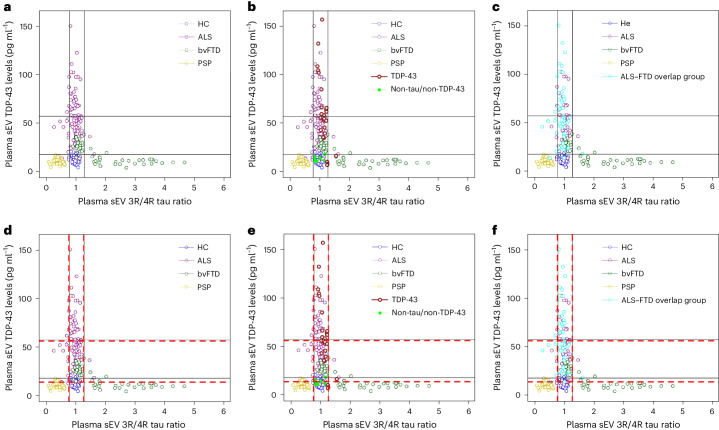
Distribution of plasma sEV 3R/4R tau ratio versus plasma EV TDP-43 levels stratified by diagnosis in the Sant Pau cohort. **a**, Sant Pau cohort without pathology-confirmed cases. Color codes indicate the different clinical diagnostic groups (ALS, bvFTD, PSP, HC). Cut-off values as determined by Gaussian mixture modeling. EV 3R/4R tau ratio cut-offs: 0.78 and 1.28; EV TDP-43 cut-offs: 17.85 pg ml^−1^ and 57.34 pg ml^−1^. **b**, Genetically confirmed cases were also plotted in the graph. **c**, The ALS–FTD overlap group is indicated in light blue. **d**–**f**, Sant Pau cohort without pathology-confirmed cases (**d**), Sant Pau cohort including pathology-confirmed cases (**e**) and Sant Pau cohort, ALS–FTD group indicated in light blue (**f**). Similar to **a**–**c** but superimposed with Sant Pau and DESCRIBE cut-offs. The black solid line indicates Sant Pau cut-offs (EV 3R/4R tau ratio cut-offs: 0.78 and 1.28; EV TDP-43 cut-offs: 17.85 pg ml^−1^ and 57.34 pg ml^−1^). The red dashed line indicates DESCIRBE subcohort 2 cut-offs (EV 3R/4R tau ratio cut-offs: 0.77 and 1.28; EV TDP-43 cut-offs: 13.87 pg ml^−1^ and 56.18 pg ml^−1^).

Plasma EV TDP-43 concentrations in bvFTD were inversely correlated to EV 3R/4R tau ratios (sEV: *r* = −0.714, *P* < 0.0001; mEV: *r* = −0.617, *P* < 0.0001; Supplementary Fig. 20a,b). A plot of plasma sEV TDP-43 concentrations versus sEV 3R/4R tau ratios without genetic cases showed a similar distribution of the diagnostic groups as observed for DESCRIBE subcohort 2 and a separation of putative bvFTD TDP and tau subgroups (Fig. 6a). The TDP-43 high bvFTD subgroup overlapped with the ALS group (Fig. 6a), cases with confirmed TDP-43 pathology (Fig. 6b) and the ALS–FTD group (Fig. 6c). Cases with confirmed non-tau/non-TDP-43 pathology overlapped with the HC group (Fig. 6b).

Cut-offs were defined by mixture modeling (Supplementary Fig. 21), excluding genetically confirmed cases for subsequent testing of cut-offs (sEV 3R/4R tau ratio: 0.78 and 1.28; sEV TDP-43: 17.85 pg ml^−1^ and 57.34 pg ml^−1^; Fig. 6d–f, black lines). Cut-offs were very close to those in DESCRIBE subcohort 2. sEV 3R/4R tau ratio: 0.77 and 1.28; sEV TDP-43 cut-offs: 13.87 pg ml^−1^ and 56.18 pg ml^−1^.

The Sant Pau TDP-43 cut-off (>17.85 pg ml^−1^) detected confirmed TDP-43 pathology cases among all individuals with a genetically proven diagnosis with a sensitivity of 88.89%, CI [70.84–97.65%] and a specificity of 85.71%, CI [42.13–99.64%].

Low plasma sEV 3R/4R tau ratios (<0.78, Sant Pau cohort cut-off) discriminated PSP cases form all other diagnoses (sensitivity: 100%, CI [91.40–100%]; specificity: 94.00%, CI [90.30–96.60%]). Similar results were obtained when applying the DESCRIBE cohort cut-off to Sant Pau data (sensitivity: 100%, CI [91.40–100%]; specificity: 94.94%, CI [91.77–97.50%]).

Fifty-eight percent of sporadic bvFTD patients in the Sant Pau cohort showed high plasma sEV 3R/4R tau ratios (>1.28), indicative of tau pathology. Of those with tau ratios below this cut-off (42%), all but one showed EV TDP-43 levels above the TDP cut-off, further supporting that sEV tau ratio and TDP-43 measurements can distinguish two separate bvFTD subgroups.

The Sant Pau EV TDP-43 cut-off identified patients with sporadic ALS and ALS–FTD with high sensitivity and specificity versus HC and PSP (ALS sensitivity: 86.15%, CI [75.34–93.47%]; specificity: 100%, CI [96.03–100%]; ALS–FTD sensitivity: 96.55%, CI [88.90–99.58%]; specificity: 100%, CI [96.03–100%]). Applying the DESCRIBE subcohort 2 cut-off values to Sant Pau resulted in a sensitivity of 89.23% (CI [79.06–95.56%]) and a specificity of 90.11% (CI [82.05–95.38%]) for ALS, and a sensitivity of 100% (CI [93.84–100%]) and specificity of 90.11% (CI [82.05–95.38%]) for ALS–FTD.

Together, these data indicate that cut-offs are nearly interchangeable between the two cohorts.

## Discussion

Currently, there are no biomarkers available for ALS, FTD and FTD spectrum disorders that define the underlying proteinopathies. A low-invasive fluid biomarker for the identification of molecular pathology would allow pathology-based stratification of patients to clinical trials and strongly advance the development of new disease-modifying therapies. In light of novel therapeutic approaches in ALS and FTD, such biomarkers are strongly desired.

Our study in a large cohort of 704 patients, including 37 genetically and 31 pathologically confirmed samples as well as in an independent validation cohort of 287 patients with 34 genetically confirmed cases, shows that plasma EVs inform about tau and TDP-43 pathology in bvFTD, and can additionally discriminate patients with ALS and PSP from healthy and neurodegenerative disease controls with high diagnostic accuracy (AUC > 0.91).

High plasma EV 3R/4R tau ratios in bvFTD were characterized by low EV TDP-43 levels and both markers separated bvFTD into two distinct groups, which can be discriminated based on cut-off values derived from mixture modeling. Plasma EV 3R/4R tau ratios in confirmed cases with TDP-43 pathology displayed low 3R/4R tau ratios, comparable with values in the HC group, but high TDP-43 levels. By contrast, *MAPT* mutation carriers showed high EV 3R/4R tau ratios and mapped to the bvFTD tau subgroup. Clinically diagnosed PSP, including neuropathologically proven cases of PSP-type tauopathy, segregated into a third group, characterized by a decreased EV 3R/4R tau ratio and EV TDP-43 levels in the range of HC.

It is unclear why the EV 3R/4R tau ratio is low in PSP but high in bvFTD cases with FTLD-tau. 4R tau predominance may explain low 3R/4R tau ratios in PSP; however, all three *MAPT* mutation carriers in DESCRIBE showed high 3R/4R tau ratios, despite being associated with 4R tau pathology. It is possible that different ‘strains’, posttranslational modifications or regional and cellular distribution of pathology may result in differential sorting of 3R and 4R tau isoforms to EVs in different tauopathies^50^.

Plasma EV TDP-43 levels distinguished ALS from all other groups with high diagnostic accuracy (AUC ≥ 0.91 versus HC, PSP, bvFTD in DESCRIBE; AUC ≥ 0.94 versus HC, PSP; and AUC ≥ 0.76 versus bvFTD in the Sant Pau cohort). Cases with confirmed TDP-43 pathology were characterized by high EV TDP-43 levels, whereas ALS cases with mutations that are not linked to TDP-43 pathology displayed low EV TDP-43 concentrations, comparable with HC.

Unexpectedly, cases with *VCP* and *TBK1* mutations failed to show increased EV TDP-43 levels, although mutations in both genes have been linked to TDP-43 pathology^47,48^. One potential explanation could be the predominance of intranuclear lentiform inclusions and impaired endolysosomal protein sorting described in *VCP* mutation carriers, which may prevent release of TDP-43 with EVs^51^. Overactivation of TBK-1 can increase neuronal EV release^52^. Thus, *TBK-1* loss-of-function mutations may impair EV secretion. Indeed, plasma EV concentrations in *TBK-1* mutation carriers were approximately three times lower than the cohorts’ mean plasma EV concentration (Supplementary Fig. 22).

In PSP and bvFTD, plasma EV tau ratios correlated with NfL, clinical, cognitive and behavior scales reflecting disease pathology, similar to plasma EV TDP-43 in ALS and bvFTD, including trial-relevant scales such as ALS-FRS-revised and CDR-SB. Plasma EV 3R/4R tau and TDP-43 may have the potential to mirror disease progression and could, after positive evaluation in longitudinal cohort samples and therapeutic intervention trials, potentially be used as a progression and surrogate marker for clinical studies.

We focused on plasma EV measurements due to several reasons:

1. Plasma albumin and immunoglobulins can interfere with antibody binding^53^ but are largely absent in plasma EV preparations^26^, which may facilitate TDP-43 detection.
2. Full-length tau concentrations in blood are extremely low because of tau fragmentation. By contrast, EV cargo is protected from degradation^54^, which allows reliable quantification of 3R and 4R tau isoforms in plasma EVs.
3. Increased EV levels of tau or TDP-43 in extracellular fluids may reflect disease pathology. Both tau and TDP-43 can be transported by EVs to other cells where they can induce protein aggregation. Cytoplasmic (mis)localization of TDP-43 is likely required for its secretion with EVs, and its release with EVs may mirror the nuclear to cytoplasmatic shift of TDP-43 observed in ALS and FTD.

Although our cohort included a high number of confirmed TDP-43 cases, future studies with additional confirmed FTLD-tau cases are needed, as well as measurements from preclinical disease stages, to determine how early these biomarkers increase before symptom onset. Cross-sectional and longitudinal samples need to be analyzed in additional, independent and ethnically more diverse cohorts (imbalanced group sizes in DESCRIBE subcohort 2: Supplementary Data).

EVs can pass the blood–brain barrier^55^ but it is not known to what extent plasma EV TDP-43 or tau stem from the CNS because both are expressed in peripheral tissue^27,56,57^. The correlation of CSF with plasma EV TDP-43 in ALS supports the notion that plasma EV TDP-43 may reflect CNS pathology. Plasma EV tau and TDP-43 are almost exclusively found in L1CAM-positive EVs, which could further support a potential brain origin, although the neuronal specificity of L1CAM and its association with EVs are controversially discussed^30^.

Strengths of our study are the large numbers of patient samples, 991 in total, and the independent validation in another cohort, containing altogether 97 genetically and/or neuropathologically confirmed samples. Our work is the first study, to our knowledge, demonstrating a blood-based, and therefore low invasive and easily accessible, fluid biomarker, which has great potential as a diagnostic marker to distinguish FTLD-TDP from FTLD-tau, and to detect ALS and PSP. Cut-off values determined by Gaussian mixture modeling were remarkably transferable between the different cohorts, particularly tau ratio cut-offs. Plasma mEVs may even offer faster and easier preparation than sEVs, although in our study, sEVs performed slightly better.

Our work may also have important implications for AD, where limbic predominant TDP-43 co-pathology has been described in up to 55% of cases^58,59^ and is associated with a more aggressive clinical course^60^. A combination of ‘classical’ AD biomarkers with plasma EV TDP-43 may help to stratify AD cases with and without limbic predominant TDP-43 co-pathology pathology.

In summary, with EV 3R/4R tau and EV TDP-43 we describe the first marker that specifically detects underlying molecular pathology in patients with ALS, FTD and FTD spectrum disorders, whereas previously suggested biomarkers reflect downstream effects such as neurodegeneration (NfL)^61,62^ or inflammation (glial fibrillary acidic protein)^63,64^.

## Methods

### Patient samples

The DZNE Clinical Registry Study of Neurodegenerative Diseases (DESCRIBE) cohort is a multicentric, longitudinal observational study conducted by the German Center for Neurodegenerative Diseases (DZNE) and its clinical sites. It recruits patients with different neurodegenerative conditions, including ALS, bvFTD and PSP. Recruitment of these patients is described in more detail below. The multicenter, longitudinal Degeneration Controls and Relatives cohort (DANCER) serves to provide HCs for all DESCRIBE subcohorts. After written informed consent (University of Bonn Ethics Board statement 311/14) all participants undergo baseline and annual follow-up visits with clinical and neurological examination, cognitive assessments, 3T magnetic resonance imaging (MRI), blood and CSF sampling following identical standard operating procedures. Patients with AD dementia were recruited as part of the DESCRIBE cohort, following the National Institutes of Aging–Alzheimer’s Association diagnosis criteria^65^ and confirmed by positive CSF amyloid-beta, total tau and p-tau181 status.

### The DESCRIBE cohort

#### The DESCRIBE ALS cohort

ALS patients were diagnosed according to the revised El Escorial Criteria^66^. Different motor phenotypes of ALS were classified as classical ALS, progressive bulbar paresis, flail arm, flail leg, progressive muscular atrophy, primary lateral sclerosis or genetic ALS. Participants were clinically characterized using the Amyotrophic Lateral Sclerosis Functional Rating Scale-revised^67^. The Edinburgh Cognitive and Behavioral ALS Screen^68^ served as an additional test to identify cognitive and behavioral impairment. ALS patients with cognitive impairment, ALS with behavioral impairment, ALS with cognitive and behavioral impairment, ALS–FTD following the Strong criteria^69^ and genetic ALS with a pathogenic FTD mutation also underwent the assessments of the DESCRIBE FTD cohort.

#### The DESCRIBE FTD cohort

Patients with bvFTD were diagnosed according to the revised Rascovsky criteria^70^ by an experienced multidisciplinary team of neurologists, psychiatrists and neuropsychologists, and under consideration of MRI and CSF data, when available. Neuropsychological assessments included MMSE, the MoCA^58^, Free and Cued Selective Reminding Test^71^, the Neuropsychological battery of the Consortium to Establish a Registry for Alzheimer’s Disease Plus test^72^ including Trail Making Tests A and B and the mini-Social cognition & Emotional Assessment test^73^. Psychiatric scales included Geriatric Depression Scale^74^, the brief questionnaire of the NPI-Q^45^, and the functional scales CDR-SB, CDR plus NACC FTLD, FAQ^42^ and a modification of the revised Cambridge Behavior Inventory^46^, the CBI-M.

Patients with svPPA were diagnosed according to Gordon-Tempini criteria^75^. Baseline assessment of patients with PPA additionally included a modified version of the Camel and Cactus test^76^, the visual form of the Sentence Comprehension Test^77^, the Sentence Repetition Test from the Aachen Aphasia Test^78^, hierarchical word lists^79^ and the Repeat and Point Test^80^.

#### The DESCRIBE PSP cohort

The cohort design is summarized in ref. ^81^. Diagnosis of PSP was based on the National Institute of Neurological Disorders and Stroke and the Society for PSP criteria^82^ for participants recruited before 2017, and on the Movement Disorder Society (MDS-PSP) diagnostic criteria^83^ for participants recruited after 2017. Participants were clinically phenotyped by the PSP-RS^35^, PSP-SS^35^, PSP-QoL^41^, PSP-CDS^36^, SEADL^37^, MDS-UPDRS Part III^39^, SAS^40^, CGI-s^38^, Geriatric Depression Scale^74^ and MoCA^34^.

#### The healthy control cohort DANCER

HC samples were obtained from DANCER and included 71 participants who, based on neuropsychological testing, neurological and psychiatric examination, do not suffer from a neurodegenerative disease. Participants additionally underwent MRI. The neuropsychological test battery follows the same protocol and includes all assessments as the one used for participants of the DESCRIBE FTD cohort. Participants undergo an annual follow-up as well as genetic testing at baseline (see below). Relatives with a known pathogenic FTD–ALS mutation were excluded as controls.

#### Genetics

All patients with a diagnosis of bvFTD, FTD–ALS, ALS with cognitive and or behavior impairment, and all control subjects were tested for pathogenic *C9orf72* hexanucleotide repeat expansions, for insertions or deletions in *MAPT* and *GRN* genes by multiplex ligation-dependent probe amplification and for other protein-coding variants by whole-exome sequencing. Specifically, expansions of the *C9orf72* GGGGCC hexanucleotide repeat were detected by the AmplideX PCR/CE C9orf72 kit (Asuragen) with a cut-off value of 30 repeats defining pathologically expanded repeats. For detection of deletions or duplications in *GRN* and *MAPT* genes we employed the SALSA multiplex ligation-dependent probe amplification kit (MRC-Holland). Participants with ALS and PSP were not systematically screened for mutations as part of the DESCRIBE study protocol. Our study sample contained 37 mutation carriers, including 18 *C9orf*, 4 *GRN*, 3 *MAPT*, 4 *VCP*, 2 *TBK1*, 2 *CHCHD10*, 2 *FUS* and 2 *SOD-1* cases (Supplementary Table 10).

#### DZNE Brain Bank postmortem cohort and neuropathological diagnosis

In the DZNE Brain Bank, autopsies and sampling of tissues for diagnostics and research is performed after written informed consent in accordance with local ethics review boards. Brain autopsies and neuropathological diagnosis were available for 31 participants from subcohort 2, consisting of 24 cases with a TDP-43 proteinopathy (ALS-TDP and FTLD-TDP, including 2 cases with *TBK1* mutation), 5 cases with a tau proteinopathy (PSP and FTLD-tau including 1 case with *MAPT* mutation), as well as 1 ALS with a mutation in *SOD-1* and 1 ALS case with a *CHCHD10* mutation (Supplementary Table 11).

Neuropathological evaluation was performed for all cases on formalin-fixed paraffin-embedded tissue sections from 20 standardized neuroanatomical regions following guidelines for the assessment and diagnosis of neurodegenerative diseases including immunohistochemistry with antibodies against phosphorylated TDP-43 (clone 1D3)^84^, phosphorylated tau (clone AT8, Thermo Fisher), α-synuclein (clone 4D6, Origene) and beta-amyloid (clone 4G8, Covance). For all cases, assessment included reporting of AD neuropathological changes^85^ and presence/regional distribution of Lewy pathology^86^. Cases with FTLD-TDP were subclassified according to current criteria^87^.

### The Sant Pau cohort

Patients with ALS were prospectively recruited from the Motor Neuron Disease Clinic at Hospital de la Santa Creu i Sant Pau. We included patients categorized as probable laboratory-supported or definite ALS according to El Escorial revised criteria^88^. ALSFRS-R in its Spanish version^89^ was systematically assessed at the time of sample acquisition. Unimpaired HCs, bvFTD and PSP patients were recruited at the Sant Pau Memory Unit and include individuals from the Sant Pau Initiative on Neurodegeneration multimodal biomarker cohort. ALS–FTD patients were recruited by Sant Apu Memory Unit and Motor Neuron Disease Clinic. Information about clinical and neuropsychological assessments and sample processing have been previously described in detail^49^. Plasma samples were obtained using the same standard operating procedure. All patient samples (ALS, ALS–FTD, bvFTD and PSP) were screened for the presence of a pathogenic repeat expansion mutation in *C9orf72*. In addition, patients with ALS were tested for mutations in genes causing ALS, FTD and AD using a gene panel. bvFTD and PSP patients underwent whole-exome sequencing. In total, pathogenic mutations were found in *C9orf72* (*n* = 16), *GRN* (*n* = 6), *SOD1* (*n* = 4), *TBK1* (*n* = 3), *FUS* (*n* = 3), *TARDBP* (*n* = 1) and *VCP* (*n* = 1). This study was approved by the Hospital de la Santa Creu i Sant Pau Ethics Committee. Written informed consent was obtained from all participants.

### EV isolation from plasma and CSF

EVs were prepared from EDTA plasma as described in ref. ^26^ by a blinded experimentator. Briefly, 500 μl of plasma was thawed on ice and subjected to serial centrifugation to isolate sEVs and mEVs. To remove cellular debris, plasma was centrifuged for 10 min at 4 °C and 3,500*g*, and twice at 4,500*g*. The supernatant was subsequently centrifuged for 30 min at 10,000*g* and 4 °C. The resulting pellet (mEV fraction) was resuspended in 100 μl of PBS, 1% CHAPS, whereas the supernatant was applied to size-exclusion columns equilibrated with 10 ml of 20 mM HEPES buffer (pH 7.4) to isolate sEVs (qEVoriginal, 70 nm+; Izon Science Limited). Using the Izon Automatic Fraction Collector and by adding 20 mM HEPES buffer (pH 7.4), we eluted 24 fractions with a volume of 500 μl. As shown previously^26^, fractions 7–10 contain the highest EV concentrations without contamination by nonvesicular plasma proteins. We therefore pooled fractions 7–10 as the sEV fraction and subjected them to 4,000g centrifugation at 4 °C in an Amicon Ultra centrifugal filter with a 3-kDa cut-off (Merck Millipore) for 40 min at 20 °C to concentrate the sample. Subsequently, the volume was filled up with 10% CHAPS in a 20-mM HEPES to a final concentration of 1% CHAPS (300 µl). Samples were divided in three, stored at −20 °C until further analysis of tau and TDP-43 content. CSF EV for correlation analysis of CSF and plasma EV tau levels were prepared from all DESCRIBE subcohort 1 cases, following the same protocol as for plasma EVs, with a starting volume of 1.5 ml of CSF. We prepared CSF EV for correlation analysis with corresponding plasma EV TDP-43 levels for all ALS cases in DESCRIBE subcohort 2 for which CSF was available (*n* = 41). A starting volume of 1 ml of CSF was used. Of note, in all cases CSF was drawn at the same visit as plasma samples.

In all cohorts, we aimed for sex-balanced and age-balanced diagnostic groups. The sex of participants was determined based on self-report.

### L1CAM immunocapture assay

Plasma (500 µl) was thawed on ice and subjected to serial centrifugation to isolate mEVs and sEVs as described above. mEVs were resuspended in 300 µl of PBS. sEV preparations were concentrated to a final volume of 300 µl, which was divided into three aliquots of 100 µl each. L1CAM immunoisolation from EV preparations was performed as described previously^90^. In brief, 100 μl of sEVs were diluted in 400 μl of double-distilled H_2_O supplemented with protease and phosphatase inhibitors and 3% bovine serum albumin. Dilutions were incubated for 60 min with 2.7 µg of biotinylated mouse anti-human CD171 (L1CAM neural adhesion protein) antibody (clone 5G3; eBiosciences) at room temperature and under constant shaking at 800 rpm. For the IgG control condition, 2.7 µg of biotinylated mouse immunoglobulin G2 (IgG2) antibody (clone eBM2a; cat. no. 13-4724-85, Thermo Fisher Scientific) was added instead of anti-human CD171 antibody. Subsequently, 26 µl of streptavidin agarose Ultralink resin (Thermo Fisher Scientific) was added followed by 60 min of incubation at room temperature and shaking at 800 rpm. Solutions were centrifuged for 10 min at 800*g* at 4 °C and pellets were resuspended for 10 s in 50 µl of cold 0.1 M glycine–HCl (pH 3.0) followed by centrifugation at 4 °C and 4,500*g* for 5 min. The supernatant was transferred to tubes containing 50 µl of 3% bovine serum albumin in 1 M Tris–HCl (pH 8.0). Aliquots of 5, 15 and 80 µl were used for nanoparticle tracking analyzer (NTA), western blot and TDP-43 or tau analysis by SIMOA or electrochemiluminescence/Meso Scale discovery (MSD)/ELISA, respectively. Before TDP-43 and tau analysis, CHAPS was added at a final concentration of 1%.

### Western blotting

Western blotting was performed according to standard protocols using 10% or 12% sodium dodecyl sulfate polyacrylamide gels, followed by transfer to polyvinylidene fluoride (PVDF) membranes (Millipore). PVDF membranes were blocked for 30 min in 4% w/v nonfat dried milk in TBS-Tween 0.5% v/v (TBS-T). Primary antibodies were incubated with the PVDF membrane overnight at 4 °C, and secondary antibodies for 1 h at room temperature. Protein bands were visualized using an ECL western blotting detection kit (GE Healthcare). The following antibodies were used. (1) Primary antibodies: anti-Calnexin (1:2,000 dilution; cat. no. C4731, Sigma-Aldrich), anti-Flotillin-2 (1:500 dilution; cat. no. 610384, BD Biosciences); anti-3R tau (RD3 anti 3R tau antibody; 1:500 dilution; cat. no. 05-803, Merck), anti-4R tau (anti-4R tau antibody; dilution 1:500; cat. no. ab218314, Abcam), and anti-TDP-43 antibody (dilution 1:500; cat. no. ab305694, Abcam). (2) Secondary antibodies: HRP antimouse IgG (1:5,000 dilution; Dako), HRP antirabbit IgG (1:5,000 dilution; Dako).

### Nanoparticle tracking analyzer

NTA was performed with a NanoSight LM10 instrument and a LM14 viewing unit equipped with a 532 nm laser (NanoSight, Malvern Instruments) by a blinded experimentator. Samples were recorded in quadruplicates for 30 s and analyzed with the NTA 2.3 software.

### Development of 3R and 4R isoform-specific tau immunoassays

We developed two sandwich immunoassays for the specific detection of 3R and 4R tau isoforms, using antibody pairs of isoform-specific tau antibodies with HT7, an antibody raised against an N-terminal, isoform-independent epitope (amino acids 159–163).

Detection of 3R and 4R tau in plasma EVs and the specificity of the antibodies was demonstrated by western blot analysis with recombinant 3R and 4R tau proteins (Supplementary Fig. 3a–e).

Optimal dilutions of capture and detection antibodies were standardized, using the checkerboard method with serially increasing dilutions of capture and detection antibodies, different dilution buffers, incubation times, temperatures and EV lysis methods. The reproducibility of each assay was tested by performing them at least three times with technical replicates. Immunoassay performance parameters such as precision, intra-assay and interassay variability, matrix effect, linearity and parallelism were determined for both 3R and 4R tau assays in plasma-derived sEVs and mEVs using three biological replicates (Supplementary Table 1).

### 3R tau immunoassay

Plasma sEV and mEV 3R tau were measured in duplicate, 50 μl per well, by a blinded experimentator. Briefly, 96-well multiarray plates (Meso Scale Discovery) were coated with RD3 anti-3R tau antibody (cat. no. 05-803, Merck) after 1:600 dilution in Dulbecco´s Phosphate Buffered Saline overnight at 4 °C. After washing three times with 0.05% Tween-20 in Dulbecco´s Phosphate Buffered Saline (PBST), plates were blocked at room temperature with 150 μl of blocking buffer per well for 1 h under shaking at 350 rpm. Protein standards were prepared from 3R recombinant tau (htau23) by serial 2× dilution in blocking buffer (7,000 pg ml^−1^ highest standard to 109.38 pg ml^−1^ lowest standard). Standards and samples were incubated at room temperature under shaking at 350 rpm for 2 h, followed by washing three times with PBST. Plates were then incubated for 1 h at room temperature with the detection antibody, biotinylated anti-total tau HT7 (product no. MN1000B, Thermo Fisher Scientific, epitope residues 159–163), at a 1:300 dilution in blocking buffer and under shaking at 350 rpm. After washing three times in PBST, 50 μl of sulfo-tagged streptavidin (Meso Scale Discovery) was added in a 1:300 dilution per well and incubated for 1 h at room temperature in the dark and under shaking at 350 rpm. Plates were then washed three times and each well was incubated with 150 µl of 2× MSD Reading Buffer T (Meso Scale Discovery). Plates were then measured using a Sector Imager 6000 and the MSD Discovery Workbench 3.0 Data Analysis Toolbox (Meso Scale Discovery).

### 4R tau immunoassay

Plasma sEV and mEV 4R tau were measured in duplicate, 50 μl per well, by a blinded experimentator. Plates (cat. no. DY008, Biotechne) were incubated with capture antibody directed against 4R tau (Abcam 4R antibody; [EPR21725], cat. no. ab218314) in 100 μl of a 1:300 dilution in plate-coating buffer (R&D DY008 kit) for 18 hours at room temperature and under constant shaking at 150 rpm. After washing three times washing in 1× washing buffer (R&D DY008 kit), blocking buffer (10× diluted in dPBS, R&D blocking buffer, containing 0.1× HAMA blocker (cat. no. ab193969, Abcam)) was added to each well and the mixture was incubated at room temperature for 1 h under shaking at 350 rpm. Wells were subsequently washed three times in washing buffer. 4R tau standard was prepared from recombinant 4R tau (htau40) by serial dilution in blocking buffer (standard 1 (7,000 pg ml^−1^) to standard 7 (109.48 pg ml^−1^)). Standard and samples were incubated for 2 h 20 min at room temperature under shaking at 350 rpm. After washing three times, wells were incubated under shaking at 350 rpm for 2 h at room temperature with detection antibody (biotinylated total tau HT7, 1:300 dilution in blocking buffer; product no. MN1000B, Thermo Fisher Scientific), followed by washing three times and incubation with streptavidin HRP (R&D Systems) for 30 min at room temperature in the dark with shaking at 350 rpm. Next, wells were washed three times and incubated for 15 min with substrate solution (R&D DY008 kit) and subsequently with stop solution. The plates were subsequently measured immediately using a BMG Fluostar ELISA reader.

### TDP-43 SIMOA assay

TDP-43 levels were determined from plasma, plasma sEV and mEV fractions using the human TDP-43 Advantage kit on a SIMOA HD-X analyzer, software v.3.1 (Quanterix) by a blinded experimentator following the manufacturer’s instructions. As per the product information, the assay was developed against TDP-43 amino acids 203–209 and the C-terminal region. Samples were thawed on ice and randomized on plates. Plasma samples were measured in duplicate, and sEV and mEV samples as singlets, 50 μl per well.

Plasma EV TDP-43 levels were sometimes low in the HC and PSP groups, with 28 sample measurements below the lower limit of quantification, most likely reflecting the absence of TDP-43 alterations in these groups. Such floor effects could be a limiting factor; however, they can be easily overcome by using larger plasma volumes (>500 μl) for sample preparation, if necessary.

### Plasma NfL SIMOA assay

Plasma NfL concentrations were determined in duplicate, as previously described^91^, using the SIMOA NF-light Advantage kit on a Quanterix HD1 analyzer (Quanterix) by a blinded experimenter according to the manufacturer’s instructions.

### Statistical analysis

Statistical analysis and data visualization were performed using Prism 7 (GraphPad Software), SPSS Statistics 21 (IBM) and R (R Foundation for Statistical Computing) software programs. The statistical tests were two-tailed and values with *P* < 0.05 were considered significant.

Comparisons of marker levels were performed using Kruskal–Wallis tests followed by Dunn’s correction for multiple comparisons because of non-Gaussian distributions. Normal distribution assumption was assessed based on visual inspection of histograms and Kolmogorov–Smirnov tests.

To assess the link between EV marker and clinical scales as well as plasma NfL, Spearman correlations were used. To illustrate associations between plasma NfL and plasma EV 3R/4R tau ratio, plasma NfL and plasma EV /TDP-43, as well as plasma EV 3R/4R tau and plasma EV TDP-43 (Figs. 1, 2 and 4 and Supplementary Figs. 5, 7, 11 and 14), monotonic regression splines (using the ‘cgam’ function from R package ‘splines’) were modeled. Notably, potential confounders (age, sex and disease duration) showed no influence on plasma biomarker levels (Supplementary Tables 3 and 7). We therefore used the nonparametric tests described above with covariate adjustment to account for violations of normal distribution assumptions and nonlinear relationships.

MedCalc software was used for computation and comparison of ROC curves, using the method of Hanley and McNeil^92^ (standard error, 95% CI for the difference and *P* value), as well as for calculation of sensitivity and specificity. Precision recall curves, area under the precision recall curve and CIs were calculated using the R code from ref. ^93^ and published prevalence estimates for the different diagnoses (PSP^94^, ALS^95^, bvFTD^96^).

The cut-off values of 3R/4R tau ratio and TDP-43 levels were defined with Gaussian mixture modeling using the R statistical software program v.3.2.1 mix tools package as previously described in ref. ^42^. First, the R boot.comp function was used to determine the number of distributions that fitted best to the data. Next, we defined data-driven cut-offs as the point at which the lines of fitted normal distributions crossed each other. Specifically, we derived three normal distributions (as suggested by bootstrapping) and determined the intersection of the middle normal distribution with the two more extreme distributions. We computed sensitivity and specificity based on the cut-offs of plasma sEV 3R/4R tau ratio and TDP-43 levels as determined by mixture modeling.

A description of the CBI-M, transmission electron microscopy, cell culture and small interfering RNA transfection, immunoprecipitation–mass spectrometry of tau, preparation of recombinant tau protein and assay validation are given in the Supplementary Methods.

### Reporting summary

Further information on research design is available in the Nature Portfolio Reporting Summary linked to this article.

## Online content

Any methods, additional references, Nature Portfolio reporting summaries, source data, extended data, supplementary information, acknowledgements, peer review information; details of author contributions and competing interests; and statements of data and code availability are available at 10.1038/s41591-024-02937-4.

### Supplementary information

Supplementary Information Supplementary Figs. 1–23.

Reporting Summary

Supplementary Tables Supplementary Tables 1–22.

Supplementary Data for Extended Data Tables 1–3. Exact *P* values for comparisons of Extended Data Tables 1–3.

## Figures and Tables

**Extended Data Table 1:**
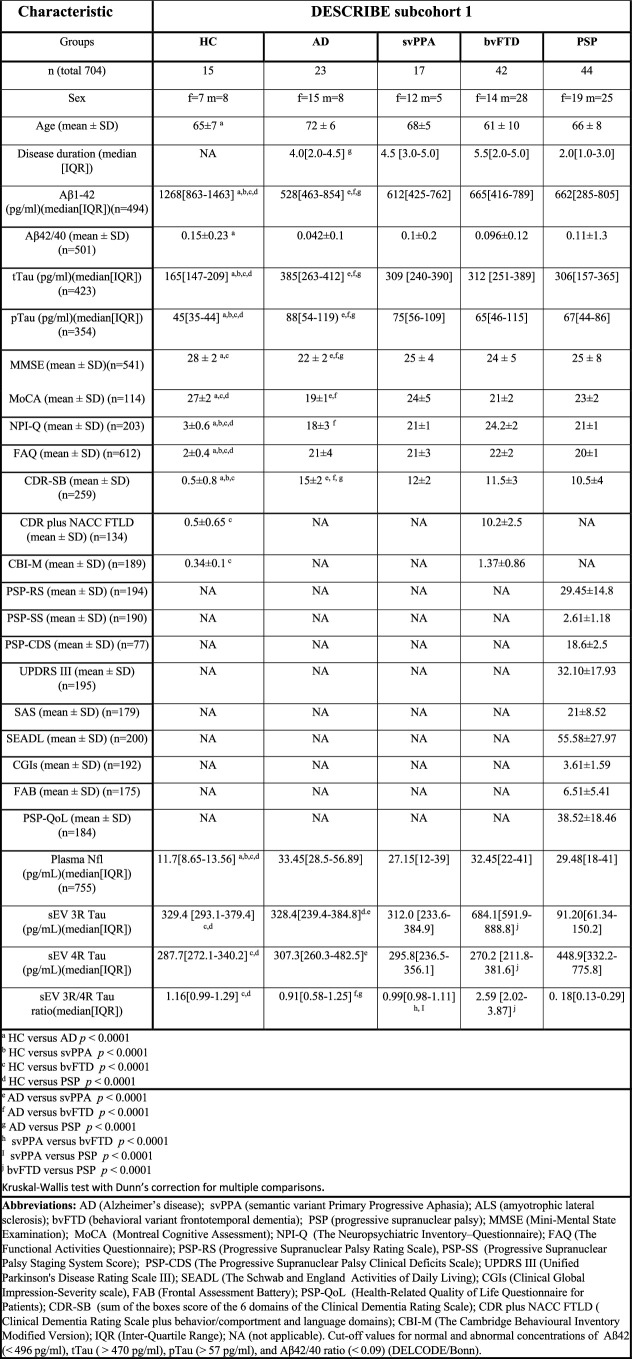
Demographic and Clinical Characteristics of All Patients in DESCRIBE subcohort 1

Demographic and Clinical Characteristics of All Patients in DESCRIBE subcohort 1.

**Extended Data Table 2:**
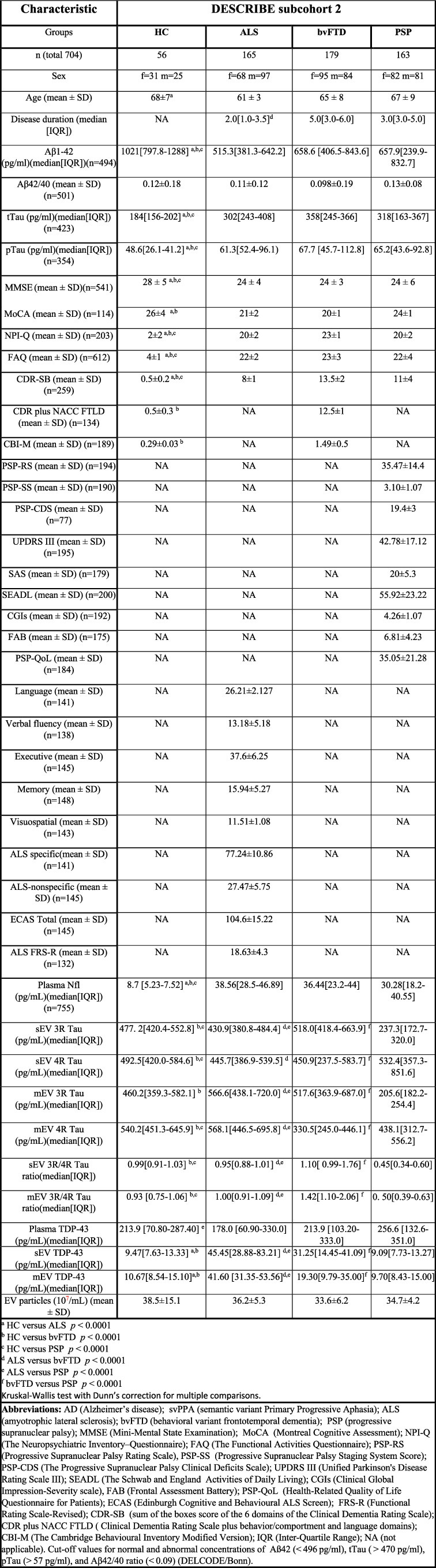
Demographic and Clinical Characteristics of All Patients in DESCRIBE subcohort 2

Demographic and Clinical Characteristics of All Patients in DESCRIBE subcohort 2.

**Extended Data Table 3:**
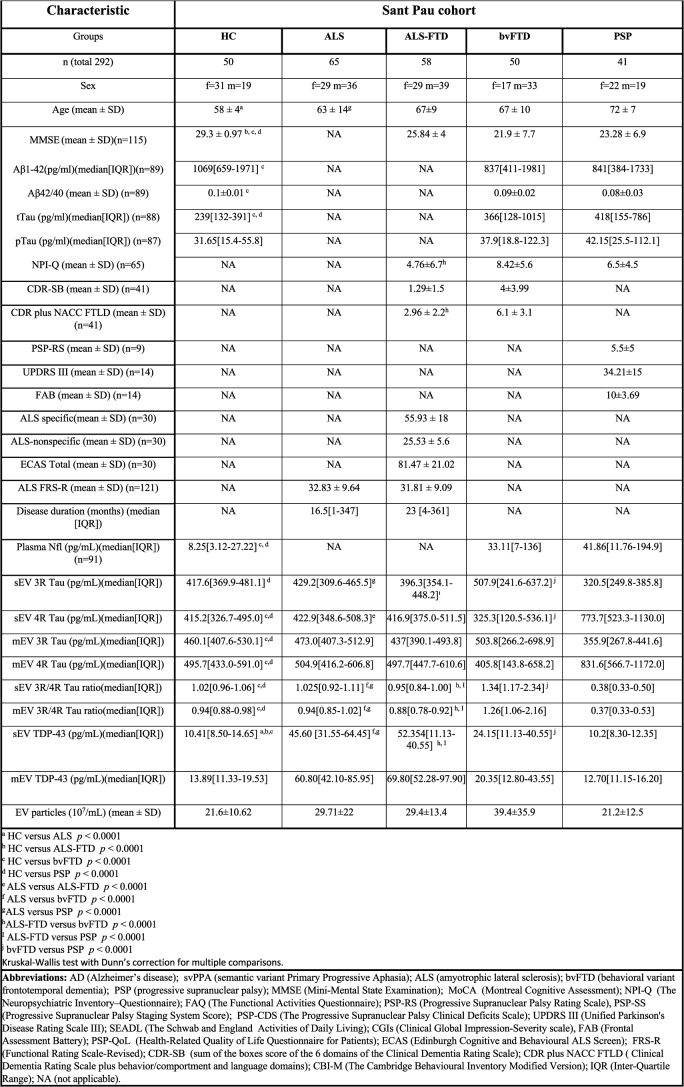
Demographic and Clinical Characteristics of All Patients in Sant Pau cohort

Demographic and Clinical Characteristics of All Patients in Sant Pau cohort.

## Data Availability

The data that support the findings of this study are available from the authors but restrictions apply to the availability of these data, and so they are not publicly available. Data are, however, available from the authors upon reasonable request and with permission from the cohorts’ steering committees (contact for and information on data access: anja.schneider@dzne.de). Expected turnover times for data applications is 3 months.

## Code availability

The code used to conduct analyses is described in Methods.
