# Supplementary material for: Plasma extracellular vesicle tau and TDP-43 as diagnostic biomarkers in FTD and ALS
Source: Nat Med. 2024 Jun 18;30(6):1771–83. doi: 10.1038/s41591-024-02937-4 (PMC11186765; doi:10.1038/s41591-024-02937-4)
Supplement: Supplementary file 1 — Supplementary Figs. 1–23. [file 41591_2024_2937_MOESM1_ESM.pdf]

nature medicine

Supplementary information https://doi.org/10.1038/s41591-024-02937-4

Plasma extracellular vesicle tau and TDP-43 as diagnostic biomarkers in FTD and ALS

In the format provided by the authors and unedited

Supplementary Files

Supplementary Results

Characterization of EV preparations

EV preparations were characterized by Western Blot for the presence of the typical EV marker protein Flotillin-2 and the absence of Calnexin to rule out contamination of the preparations with microsomal fractions (Suppl. Fig.1a, b). Nanoparticle tracking analysis (NTA) revealed a size range of 80 to 150 nm for the sEV preparation and of 100 to 400 nm for the mEV fraction (Suppl. Fig.1c, d). Transmission electron microscopy (TEM) showed the typical cup-shaped morphology of EVs (Suppl. Fig.1e, f).

Characterization of EV Tau by LC-MS/HRMS

Since the lipid bilayer membrane can protect EV cargo from degradation, we hypothesized that EVs may contain full-length Tau and thus allow the quantification of Tau isoforms, which is otherwise hampered by Tau fragmentation. We solubilized plasma and CSF EVs prepared from 10 ml of pooled samples with 0.5% NP40 and 2.5 mM guanidine prior to Tau immunopurification. To determine the abundance of 3 and 4R Tau isoforms (Suppl. Fig.2a) and Tau fragmentation status, we performed liquid chromatography and tandem high resolution mass spectrometry (LC-MS/HRMS) (Suppl. Fig.2b). Eleven Tau peptides were detected, including peptides specific to the repeat region (peptides 7-10). 4R Tau isoforms are characterized by the presence of the second repeat and were identified by detection of peptides 8 (residues 282-290 in the second repeat) and 9 (residues 299-321 spanning the transition from the second to third repeat) (Suppl.Fig.2b). In neat CSF, extremely low levels were detected for peptides 7-10 which cover the repeat region, compared to mid domain specific peptides 1-5 (Suppl.Fig. 2c). This data is consistent with the previously described fragmentation of Tau's microtubule binding repeat region in extracellular fluids, including plasma[1]. In contrast, in CSF sEV and mEV fractions, 4R Tau specific peptides 8 and 9 were highly abundant, suggesting that CSF EVs mainly contain the full microtubule-binding repeat region of Tau (Suppl. Fig.2b).

Plasma EVs contain TDP-43

We first tested the specificity of the SIMOA assay in SY5Y cell lysates upon RNAi-mediated down-regulation of endogenous TDP-43 with scrambled siRNA as a control. We found RNAi-mediated downregulation of TDP-43 as quantified by Western blot analysis (86.12% compared to scrambled control, $p<0.0001$) and SIMOA assay (81.29% downregulation, $p<0.0001$) (Suppl. Fig.10a,c). In contrast, spiking of SY5Y cell lysates with recombinant TDP-43 protein resulted in appropriate increase of TDP-43 protein concentrations as determined by SIMOA assay (96.33% recovery) (Suppl. Fig.12d). We then tested the assay performance specifically with plasma sEV and mEV preparations, as the manufacturer's assay performance parameters were determined for plasma, not EVs (Suppl. Table 1).

Gaussian Mixture Modelling

Mixture modeling has been successfully used in research on Alzheimer's disease to derive cut-offs for amyloid pathology in a setting where a gold standard neuropathological outcome has been unavailable[2]. Cut-offs were derived in a sample excluding neuropathologically or genetically confirmed cases which was subsequently used for validating cut-offs. In line with visual inspection (Suppl. Fig.15), bootstrapping suggested that the distributions of sEV 3R/4R Tau ratios and sEV TDP-43 were best approximated by three normal distributions since it showed a significantly better fit to the data compared to a single Gaussian distribution (plasma sEV 3R/4R tau ratio: p=0.001; sEV TDP-43: p=0.01) or two Gaussians (plasma sEV 3R/4R tau ratio: p=0.001; sEV TDP-43: p=0.01) but was not

further improved by modeling four Gaussian distributions (plasma sEV 3R/4R tau ratio: p=0.06; sEV TDP-43: p=0.2). Cut-offs were derived based on the intersection of the middle Gaussian distribution with the two more extreme distributions

Suppl. Discussion

Differences in group sizes (DESCRIBE cohort)

Group sizes in DESCRIBE subcohorts 1 and 2 were imbalanced which could have impacted the precision of the AUC estimates. We compared AUC results and 95% CI intervals between DESCRIBE subcohorts and the Sant Pau cohort which has more balanced diagnostic group sample sizes. As shown in Suppl. Table 20 even based on the lower bounds of the 95% CI intervals, plasma EV Tau ratio and plasma EV TDP-43 levels showed a good discriminative performance. Furthermore, in the Sant Pau cohort, we obtained AUCs and CIs comparable to DESCRIBE subcohort 2. In addition, we calculated precision recall curves and area under the precision recall curve (AUPRC) for all cohorts, since AUPRC reflect imbalanced group sizes (Suppl. Tables 21 and 22). AUPRC values were above 0.8 for plasma sEV Tau ratios and TDP-43 levels in both DESCRIBE subcohorts, further supporting the very good diagnostic performance of both markers.

Supplementary Methods

Modified version of the Cambridge Behavioural Inventory (CBI-M)

In the DESCRIBE-FTD cohort, we used a modified 50-item version of the CBI-R. Before including this new version into the analyses, we conducted a principal component analysis (PCA) with varimax rotation to confirm the theoretical factor structure. Participants with over 20% missing rate over all items were removed. In addition, one item with over 20% missing rate across all participants was excluded. Four further items were excluded due to the low factor loadings and cross-loadings. Therefore, the modified version (CBI-M) resulted in 45 items with 12 new items and 33 items from the CBI-R. The total CBI-M score was calculated by the mean of all available item scores for each bvFTD participant.

Transmission electron Microscopy (TEM)

TEM was performed as described previously[3]. Formvar coated copper grids (150 hexagonal mesh, Science Services, Munich, Germany) were incubated for 10 min on top of 10 µl droplets containing EV preparations. After 5 times PBS washing, grids were incubated first on water droplets and then 5 min on uranyl acetate-oxalate droplets (1:9 dilution of 4% uranyl acetate in 2% methylcellulose). Samples were imaged with a LEO912 transmission electron microscope (Carl Zeiss Microscopy, Oberkochen, Germany) equipped with an on-axis 2k CCD camera (TRS, Moorenweis, Germany).

Cell Culture&siRNA transfection

Cells of the human neuroblastoma-derived SH-SY5Y cell line were maintained in DMEM, supplemented with 10% fetal bovine serum (FBS), 1mM glutamine, 100U/mL penicillin and 100 µg/mL streptomycin in a humidified incubator with 5% CO_2 at 37°C.

For siRNA-mediated knock-down of TDP-43, the following siRNA sequences were used[4]:

siTDP-43 sense: GCGGGAAAAGUAAAAGAUGUU

siTDP-43 antisense: AACAUCUUUUACUUUUCCCGC)

scrambled siRNA sense: AUCCCGCUAGGCCAUUCAAGUU

scrambled siRNA antisense: AACUUGAAUGGCCUAGGGGAU.

Scrambled siRNA was used as a negative control. siRNAs were transfected into $1x10^6$ SH-SY5Y cells using RNAiMax transfection reagent (Invitrogen, catalog# 13778150) according to the manufacturer's instructions. The cells were lysed with Tris-HCl, pH 8.0, 250 mM NaCl, 0.5% NP-40, 1 mM EDTA, 1 mM

DTT, and 1× protease inhibitors (Thermo Scientific, catalog#A32965) 72 hours after transfection. The siRNA knockdown efficiency was evaluated by Western Blotting using BioRad Image lab software (Image Lab 6.1 (BioRad, USA).

Immunoprecipitation-Mass Spectrometry (IP-MS)

Tau was immunopurified, then digested as previously described and multiple tau peptides were quantified using high resolution mass spectrometry (MS)[5] from plasma and CSF sEV and mEV preparations (pooled samples, corresponding to 10 ml starting volume of plasma and CSF, respectively). Prior to immunopurification, 2.5 ng of fully 15 N-labeled 441(2N4R) tau internal standard was mixed with CSF or plasma fractions and diluted in 0.5% NP40, 2.5 mM guanidine and protease inhibitors. Tau was immunopurified by incubating the samples with Tau1 (provided by Drs. Nicholas Kanaan and Lester Binder) and HJ8.5 (provided by Dr. David Holtzman) antibodies at room temperature for 4 hours (3 mg antibody per g of beads)[6]. Immunopurified tau was digested for 16 hours at 37°C with 400 ng of trypsin (Promega). The peptide mixture was purified by solid phase extraction on C18 TopTip (Glygen Corp, Columbia, MD). Eluates were dried, resuspended and transferred in MS vials. Samples were subjected to liquid chromatography and tandem high resolution mass spectrometry (LC-MS/HRMS) analysis on a nanoAcquity UPLC system (Waters, Mildford, Massachusetts) coupled to an Orbitrap Tribrid Fusion MS (Thermo Scientific, San Jose, California) operating in PRM mode. MS/HRMS transitions were extracted using Skyline version 22.2.2.278 (MacCoss lab, University of Washington). Tau peptides concentrations were calculated using measured ratios between MS/HRMS transitions of endogenous non-phosphorylated peptides and 15N labeled peptides from the protein internal standard on peptides 151-155, 156-163, 181-190, 195-209, 212-221, 226-230, 260-267, 282-290, 299-321, 354-369 and 396-406.

Preparation of recombinant Tau protein

hTau23 (0N3R, 352 residues) is the shortest Tau ((UniProt ID 10636) isoform in human CNS containing 3 repeats, whereas hTau40 (2N4R, 441 residues) is the longest Tau isoform with 4 repeats and two N-terminal inserts (Suppl. Fig. 2). Recombinant Tau protein was prepared from Escherichia coli BL21(DE) strains expressing either hTau23 or hTau40 as described[7]. Purity of recombinant Tau and isoform specificity of 3R and 4R Tau antibodies were analyzed by Western Blotting.

Assay Validation

Assay validation was performed according to the guidelines published in Andreasson *et* al.[8]. **Sensitivity:** For the determination of the lower limit of quantification (LLOQ) of each assay, 16 blank samples were measured on one plate. The calibration curves were calculated using a four-parameter logistic curve fit for all assays, which gave the optimal fit. LLOQ was calculated as the concentration corresponding to the 2.5 signal standard deviation above the background (zero calibrator **Precision:** Intra-assay variation (repeatability) was determined by analysis of samples (n=5) in four replicates on one plate. Inter-assay variation (intermediate precision) was measured to determine the variation of analyses between 5 different days. **Dilutional linearity:** Three different EV samples were used in duplicates to perform the dilution linearity experiments. The dilutional linearity (dilutions 2x, 4x and 8x) was calculated as follows: %Linearity = [observed C *dilution factor/ previous observed C*previous dilution factor] *100; C = concentration(pg/mL). **Recovery:** Three different plasma EV samples, measured in duplicates, were spiked with recombinant 3R Tau, 4R Tau, and TDP43 calibrator at three different concentrations (low: 3R and 4R Tau 1,750 pg/mL, TDP-43 1,250 pg/mL; medium: 3R and 4R Tau 3,500 pg/mL, TDP-43 2,500 pg/mL; high: 3R and 4R Tau 7,000 pg/mL, TDP-43 5,000 pg/mL). For neat samples, the buffer was spiked instead of the calibrator. Spike recoveries were calculated according to the formula: % Recovery = [C spike sample-C neat sample /theoretical C spike] *100; C = concentration (pg/mL). **Parallelism:** Three different EV samples with high endogenous protein concentrations were serially diluted (2x, 4x and 8x). Both reciprocal relative dilution factor and OD450 absorbance signals of the samples and calibrator were log-transformed and linear regression was performed to calculate the slopes of the sample and calibrator curves. The

slope of the linear parts of the log–log transformed calibrator and sample dilution series were compared to determine the degree of parallelism by calculating the "in range%" using the following formula: in range% = [slope of sample dilution /series slope of calibration curve] *100.

Supplementary References

1 Barthelemy, N. R., Horie, K., Sato, C. & Bateman, R. J. Blood plasma phosphorylated-tau isoforms track CNS change in Alzheimer's disease. *J Exp Med* **217** (2020). https://doi.org:10.1084/jem.20200861

2 Bertens, D., Tijms, B. M., Scheltens, P., Teunissen, C. E. & Visser, P. J. Unbiased estimates of cerebrospinal fluid beta-amyloid 1-42 cutoffs in a large memory clinic population. *Alzheimers Res Ther* **9**, 8 (2017). https://doi.org:10.1186/s13195-016-0233-7

3 Stuendl, A. *et al.* alpha-Synuclein in Plasma-Derived Extracellular Vesicles Is a Potential Biomarker of Parkinson's Disease. *Mov Disord* **36**, 2508-2518 (2021). https://doi.org:10.1002/mds.28639

4 Kawaguchi, T. *et al.* Changes to the TDP-43 and FUS Interactomes Induced by DNA Damage. *J Proteome Res* **19**, 360-370 (2020). https://doi.org:10.1021/acs.jproteome.9b00575

5 Barthelemy, N. R. *et al.* Site-Specific Cerebrospinal Fluid Tau Hyperphosphorylation in Response to Alzheimer's Disease Brain Pathology: Not All Tau Phospho-Sites are Hyperphosphorylated. *J Alzheimers Dis* **85**, 415-429 (2022). https://doi.org:10.3233/JAD-210677

6 Sato, C. *et al.* Tau Kinetics in Neurons and the Human Central Nervous System. *Neuron* **97**, 1284-1298 e1287 (2018). https://doi.org:10.1016/j.neuron.2018.02.015

7 Friedhoff, P., Schneider, A., Mandelkow, E. M. & Mandelkow, E. Rapid assembly of Alzheimer-like paired helical filaments from microtubule-associated protein tau monitored by fluorescence in solution. *Biochemistry* **37**, 10223-10230 (1998). https://doi.org:10.1021/bi980537d

8 Andreasson, U. *et al.* A Practical Guide to Immunoassay Method Validation. *Front Neurol* **6**, 179 (2015). https://doi.org:10.3389/fneur.2015.00179

9 Cummings, J. L. *et al.* The Neuropsychiatric Inventory: comprehensive assessment of psychopathology in dementia. *Neurology* **44**, 2308-2314 (1994). https://doi.org:10.1212/wnl.44.12.2308

10 Pfeffer, R. I., Kurosaki, T. T., Harrah, C. H., Jr., Chance, J. M. & Filos, S. Measurement of functional activities in older adults in the community. *J Gerontol* **37**, 323-329 (1982). https://doi.org:10.1093/geronj/37.3.323

11 Golbe, L. I. The Medical Advisory Board of the Society for Progressive Supranuclear Palsy. A clinical rating scale and staging system for progressive supranuclear palsy. . *Neurology* **48:A326.** (1997).

12 Goetz, C. G. *et al.* Movement Disorder Society-sponsored revision of the Unified Parkinson's Disease Rating Scale (MDS-UPDRS): Process, format, and clinimetric testing plan. *Mov Disord* **22**, 41-47 (2007). https://doi.org:10.1002/mds.21198

13 Starkstein, S. E. *et al.* Reliability, validity, and clinical correlates of apathy in Parkinson's disease. *J Neuropsychiatry Clin Neurosci* **4**, 134-139 (1992). https://doi.org:10.1176/jnp.4.2.134

14 Schrag, A. *et al.* Measuring quality of life in PSP: the PSP-QoL. *Neurology* **67**, 39-44 (2006). https://doi.org:10.1212/01.wnl.0000223826.84080.97

15 Folstein, M. F., Folstein, S. E. & McHugh, P. R. "Mini-mental state". A practical method for grading the cognitive state of patients for the clinician. *J Psychiatr Res* **12**, 189-198 (1975). https://doi.org:10.1016/0022-3956(75)90026-6

16 Nasreddine, Z. S. *et al.* The Montreal Cognitive Assessment, MoCA: a brief screening tool for mild cognitive impairment. *J Am Geriatr Soc* **53**, 695-699 (2005). https://doi.org:10.1111/j.1532-5415.2005.53221.x

17 Golbe, L. I. & Ohman-Strickland, P. A. A clinical rating scale for progressive supranuclear palsy. *Brain* **130**, 1552-1565 (2007). https://doi.org:10.1093/brain/awm032

18 Piot, I. *et al.* The Progressive Supranuclear Palsy Clinical Deficits Scale. *Mov Disord* **35**, 650-661 (2020). https://doi.org:10.1002/mds.27964

19 Schwab RS, E. A. Projection technique for evaluating surgery in Parkinson's disease. . *In: Billingham FH, Donaldson MC, editors. Third Symposium on Parkinson's Disease. Edinburgh: Churchill Livingstone*, 152–157 (1969).

20 Guy, W. The clinical global impression scale. *In: ECDEU Assessment Manual for Psychopharmacology - Revised (DHEW Publ No ADM 76-338).* (1976).

21 Hughes, C. P., Berg, L., Danziger, W. L., Coben, L. A. & Martin, R. L. A new clinical scale for the staging of dementia. *Br J Psychiatry* **140**, 566-572 (1982). https://doi.org:10.1192/bjp.140.6.566

22 Knopman, D. S., Weintraub, S. & Pankratz, V. S. Language and behavior domains enhance the value of the clinical dementia rating scale. *Alzheimers Dement* **7**, 293-299 (2011). https://doi.org:10.1016/j.jalz.2010.12.006

23 Wear, H. J. *et al.* The Cambridge Behavioural Inventory revised. *Dement Neuropsychol* **2**, 102-107 (2008). https://doi.org:10.1590/S1980-57642009DN20200005

Supplementary Figures and figure legends

Supplementary Figure 1

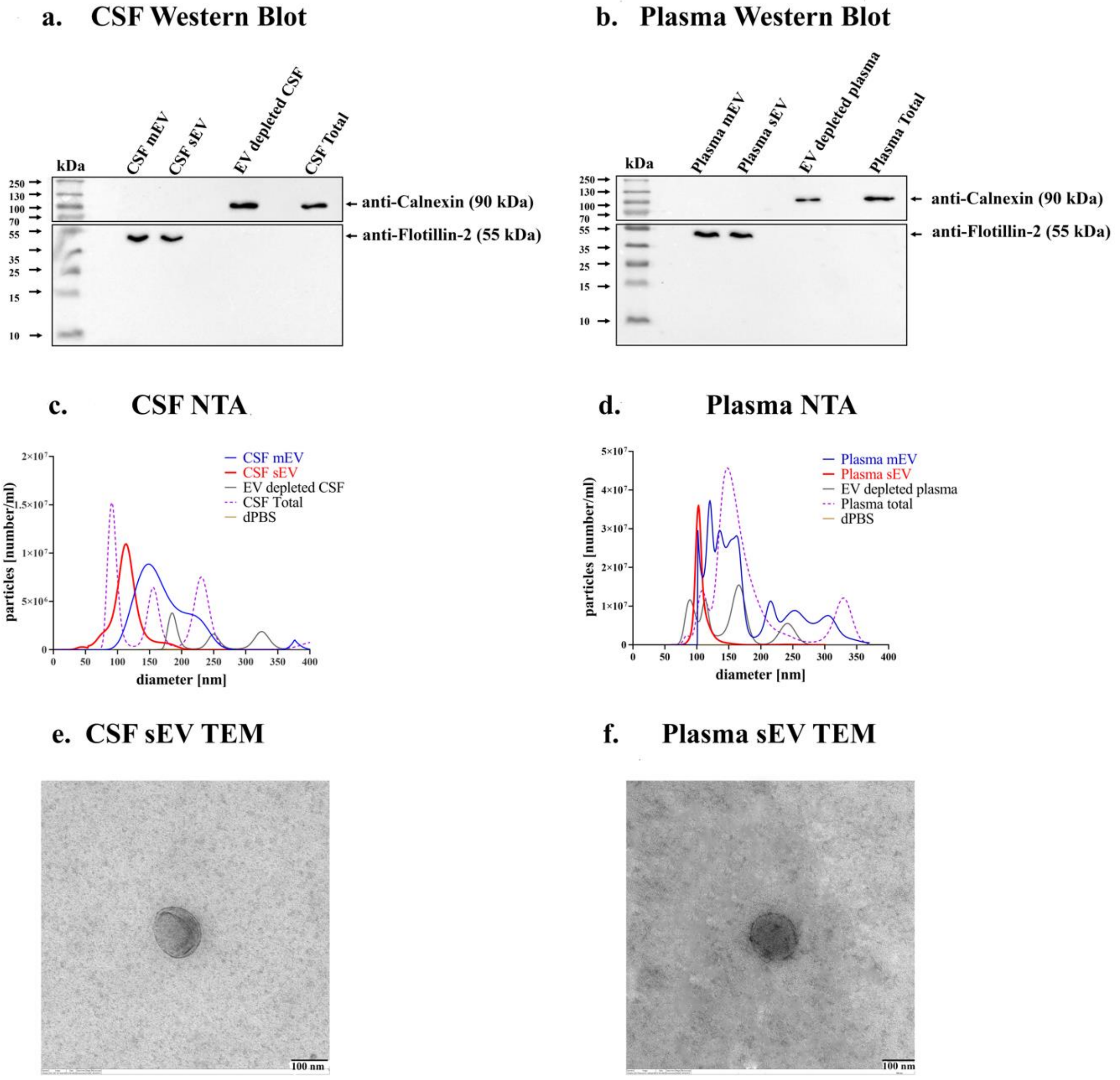

Suppl. Figure 1. Characterization of CSF and plasma EVs (CSF left column); plasma (right column)), using **(a,b)** Western blotting (WB) (n=3 independent experiments), **(c,d)** nanoparticle tracking analysis (NTA)(n=3 independent experiments), and **(e,f)** transmission electron microscopy (TEM) (n=2 independent experiments).

Supplementary Figure 2

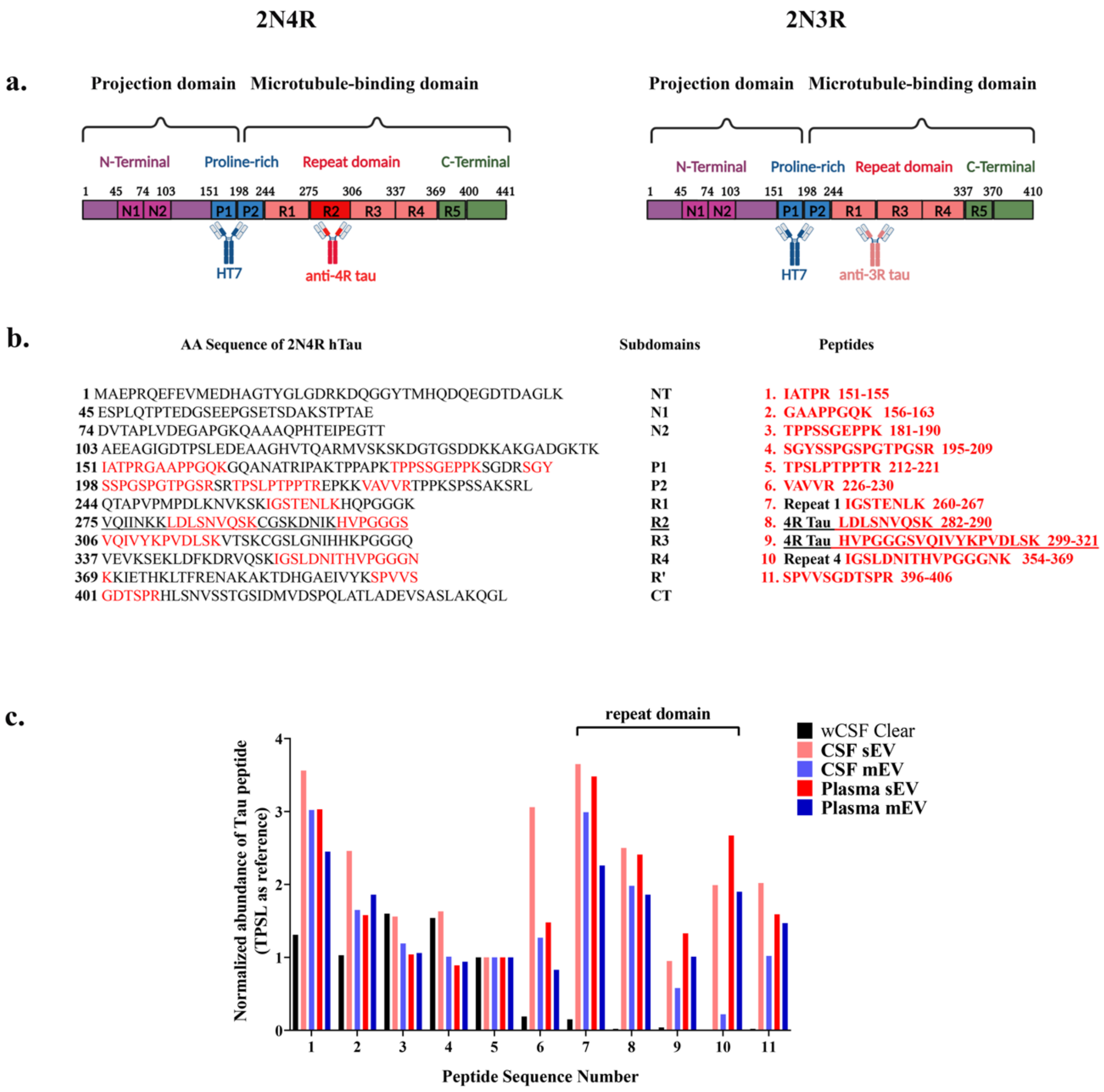

Suppl. Figure 2. EVs contain full-length Tau Amino acid sequence, subdomains and tryptic peptides (red) of 4 Repeat and 3 Repeat isoform human Tau. **(a)** NT: N-terminus; N1, N2: N-terminal inserts; P1, P2: proline rich regions; R1-4: repetitive amino acids; R' pseudo-repeat; CT: C-terminus. 3R Tau isoforms are characterized by R1,R3,R4; 4R Tau by an additional repeat domain, R2. Anti-3R Tau antibody binds to an epitope spanning R1 and R3; anti-4R Tau is specific to R2. **(b)** Left: amino acid sequence of 2N4R human Tau, the longest Tau isoform. Right: Tryptic peptides. Peptide 7 is localized in R1, peptide 8 and 9 are specific to 4R Tau, peptide 10 is localized in repeat 4. **(c)** IP-MS results from neat CSF, CSF-derived mEV, sEV, plasma-derived mEV, sEV. Abundance of Tau peptides normalized to Tau mid-domain (peptide 5).

Supplementary Figure 3

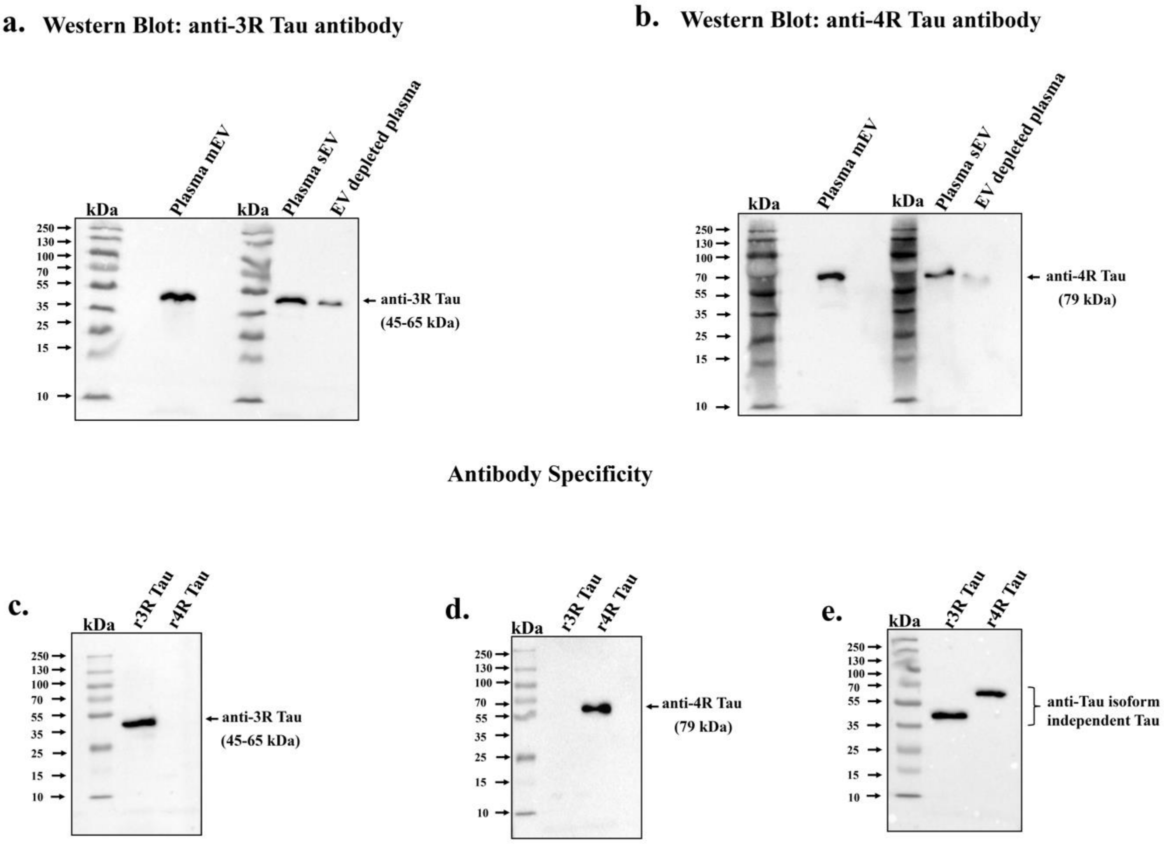

Suppl. Figure 3. 3R and 4R Tau in plasma mEV and sEV. **(a)** Western blot analysis of (lanes from left to right) plasma mEVs, sEVs, and EV-depleted plasma with antibodies directed against the 3R Tau isoform (n=3 independent experiments). **(b)** Western blot analysis of (lanes from left to right) plasma mEVs, sEVs, and EV-depleted plasma with antibodies directed against the 4R Tau isoform (n=3 independent experiments). **(c)** 3R Tau antibody specificity tested by Western blot analysis with recombinant 3R and 4R Tau proteins (n=3 independent experiments). **(d)** 4R Tau antibody specificity tested by Western blot analysis with recombinant 3R and 4R Tau proteins (n=3 independent experiments). **(e)** Western blot of recombinant 3R and 4R Tau with isoform-independent antibody HT7 (n=3 independent experiments).

Supplementary Figure 4

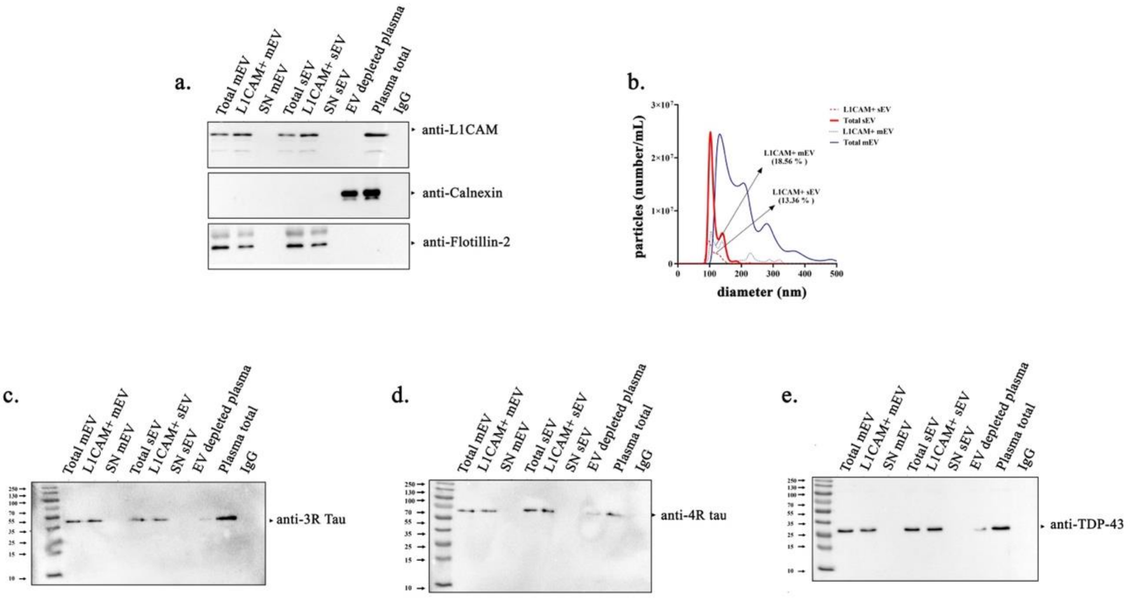

Suppl. Figure 4. Detection of 3R, 4R Tau and TDP-43 in L1CAM immunoisolated plasma EVs (a) Preparation of total plasma EVs, L1CAM-immuno-isolated EVs from plasma EV preparations, supernatant (SN) of L1CAM beads after incubation with plasma EV preparations and centrifugation (=L1CAM EV cleared fraction) (Left to right: Total plasma mEV, L1CAM positive mEV, SN mEV (supernatant of L1CAM beads incubated with plasma mEV preparations after centrifugation, corresponding to L1CAM EV cleared supernatant), total plasma sEV, L1CAM positive sEV, SN sEV (supernatant of L1CAM beads incubated with plasma sEV preparations, after centrifugation), EV depleted plasma (after SEC preparation of total plasma EVs), plasma total, IgG bead immuno-isolation of total plasma sEVs as negative control for L1CAM bead immuno-isolation). WB analysis of L1CAM, Calnexin, and Flotillin-2. WB analysis revealed, that L1CAM was 2.40 fold enriched in the L1CAM-EV preparation obtained from mEVs (2.4±0.16, n=3 independent experiments) and 3.2 fold in L1CAM EVs prepared from sEVs (3.2±0.23, n=3 independent experiments; representative WB). **(b)** NTA analysis of L1CAM immuno-isolated plasma sEVs and mEVs, total plasma sEVs and mEVs. L1CAM EVs represent a subpopulation of total plasma EVs, based on NTA analysis of EV numbers in the different preparations (sEV: mean particle concentrations in L1CAM EV preparations: 3.77E+08±5.86E+03 SD particles/ml and total plasma EV 2.82E+09±1.53E+05 particles/ml; mEV: mean particle concentrations in L1CAM EV preparations: 5.31E+08±3.87e+03 particles/ml and total plasma EV 2.86E+09±4.82+06 particles/ml). **(c)** WB analysis of 3R-Tau and **(d)** 4R Tau (Left to right: Total plasma mEV, L1CAM positive mEV, SN mEV (supernatant of L1CAM beads incubated with plasma mEV preparations after centrifugation), total plasma sEV, L1CAM positive sEV, SN sEV (supernatant of L1CAM beads incubated with plasma sEV preparations after centrifugation), EV depleted plasma (after SEC preparation of total plasma EVs), plasma total, IgG bead immuno-isolation of total plasma sEVs as negative control for L1CAM bead immuno-isolation). **(e)** WB analysis of TDP-43 (Left to right: Total plasma mEV, L1CAM positive mEV, SN mEV (supernatant of L1CAM beads incubated with plasma mEV preparations after centrifugation), total plasma sEV, L1CAM positive sEV, SN sEV (supernatant of L1CAM beads incubated with plasma sEV preparations after centrifugation), EV depleted plasma (after SEC preparation of total plasma EVs), plasma total, IgG

bead immuno-isolation of total plasma sEVs as negative control for L1CAM bead immuno-isolation). Unprocessed WB: Suppl. Fig. 23.

Supplementary Figure 5: DESCRIBE subcohort 1

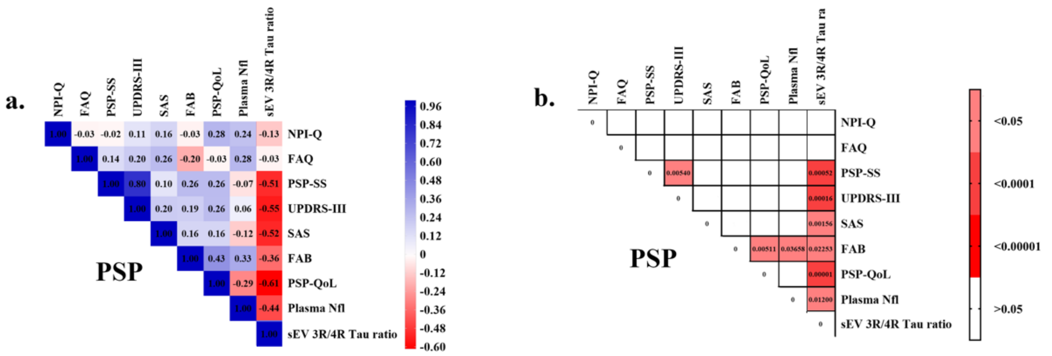

Suppl. Figure 5. Correlation matrix Results of two-sided Spearman correlations between different clinical measures and sEV 3R/4R ratio, visualized by plotting strength of correlation (*r*) as a heat map along with *p* values (right). PSP: Neuropsychiatric Inventory Questionnaire (NPI-Q)[9], Functional Activities Questionnaire score (FAQ)[10], PSP staging system (PSP-SS)[11], MDS-Unified Parkinson's Disability Rating Scale (MDS-UPDRS) Part III[12], Starkstein Apathy Scale (SAS)[13], PSP quality of life scale (PSP-QoL)[14].

Supplementary Figure 6: DESCRIBE subcohort 1

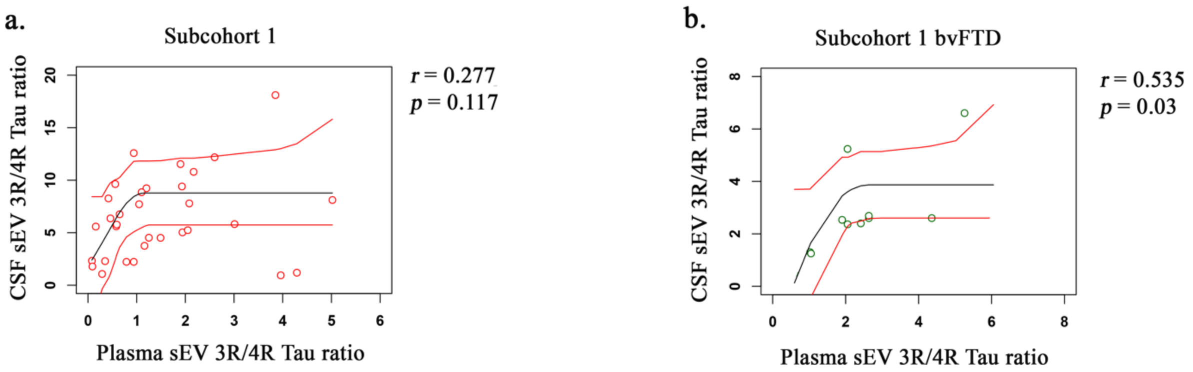

Suppl. Figure 6. CSF and plasma EV Tau ratio correlations Two-sided Spearman correlation analysis of associations and monotonic regression splines between CSF sEV 3R/4R Tau ratio and plasma sEV 3R/4R Tau ratio in **DESCRIBE subcohort 1** where CSF had been obtained in parallel to blood sampling (total number of samples n=141). The majority of CSF sEV 4R Tau levels were below the assay's detection limit and EV 3R/4R Tau ratios could not be calculated (CSF sEV 3R Tau detectable cases n=100, CSF sEV 4R Tau detectable cases n=34; bvFTD number of total cases in subcohort 1 n=42, CSF sEV 4R Tau detectable cases n=10. **(a)** CSF to plasma sEV 3R/4R Tau correlation, all diagnostic groups (n=34), **(b)** CSF to plasma sEV 3R/4R Tau correlation in the bvFTD group only (n=10). No significant correlation was found between CSF and plasma sEV Tau ratios across all diagnostic groups of subcohort 1 in samples which had detectable CSF EV 4R Tau levels (sEV: r=0.277, p=0.117). In the bvFTD group. CSF sEV Tau ratios correlated significantly with the corresponding plasma sEV Tau ratios (sEV: r=0.535, p=0.034). CSF sEV 3R Tau levels correlated with plasma EV sEV 3R Tau (r=0.24, p=0.016).

Supplementary Figure 7: DESCRIBE subcohort 2

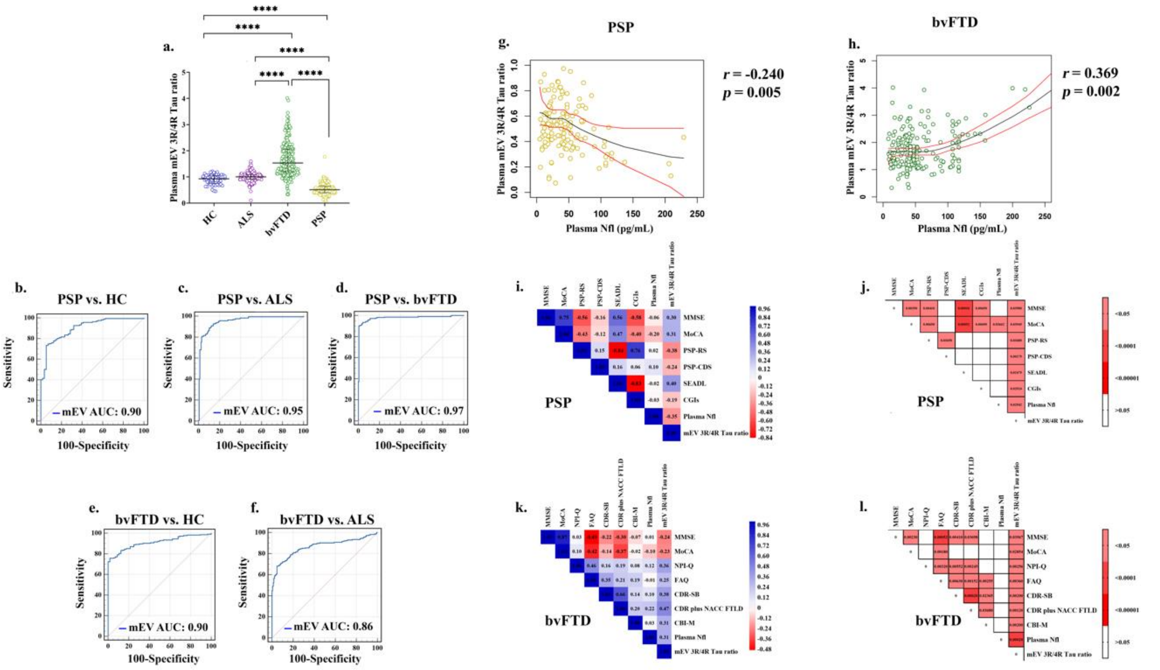

Suppl. Figure 7. 3R/4R Tau ratio in plasma mEV in **DESCRIBE subcohort 2 (a)** Biologically independent samples: HC n= 56, ALS n=165, bvFTD n= 179, PSP n=163. Horizontal lines: median and inter-quartile range (IQR). Kruskal-Wallis test with Dunn's correction for multiple comparisons (HC vs. bvFTD *p*=0.0000052, HC vs. PSP *p*=0.0000012, ALS vs. bvFTD *p*=0.0000097, ALS vs. PSP *p*=0.0000056, bvFTD vs. PSP *p*=0.0000041; **** *p*<0.00001). **(b-f)** Receiver Operating Characteristic (ROC) curve for mEV 3R/4R Tau ratio: **(b)** PSP vs. HC **(c)** PSP vs. ALS **(d)** PSP vs. bvFTD **(e)** bvFTD vs. HC**(f)** bvFTD vs. ALS **(g-h)** Two-sided Spearman correlation analysis and monotonic regression splines were performed between mEV 3R/4R ratio and plasma Nfl levels within **(g)** PSP and **(h)** bvFTD diagnostic groups. **(i-l)** Correlation matrix depicting results of two-sided Spearman correlations, visualized by plotting strength of correlation (*r*) as a heat map along with *p* values (right). **(i, j)** PSP and **(k, l)** bvFTD. PSP: Mini Mental State Examination (MMSE)[15], Montreal Cognitive Assessment (MoCA)[16], PSP rating scale (PSP-RS)[17], PSP-clinical deficits scale (PSP-CDS)[18], Schwab and England disability scale (SEADL)[19], the Clinical Global Impression Severity Scale (CGI-s)[20]; bvFTD: MMSE, MoCa, Neuropsychiatric Inventory Questionnaire (NPI-Q)[9], Functional Activities Questionnaire score (FAQ)[10], Clinical Dementia Rating-Sum of Boxes score (CDR-SB)[21], CDR plus NACC FTLD, previously termed CDR-SB FTD[22], modified version of the Cambridge Behavior Inventory-Revised Version[23] (CBI-M).

Supplementary Figure 8: DESCRIBE subcohort 2

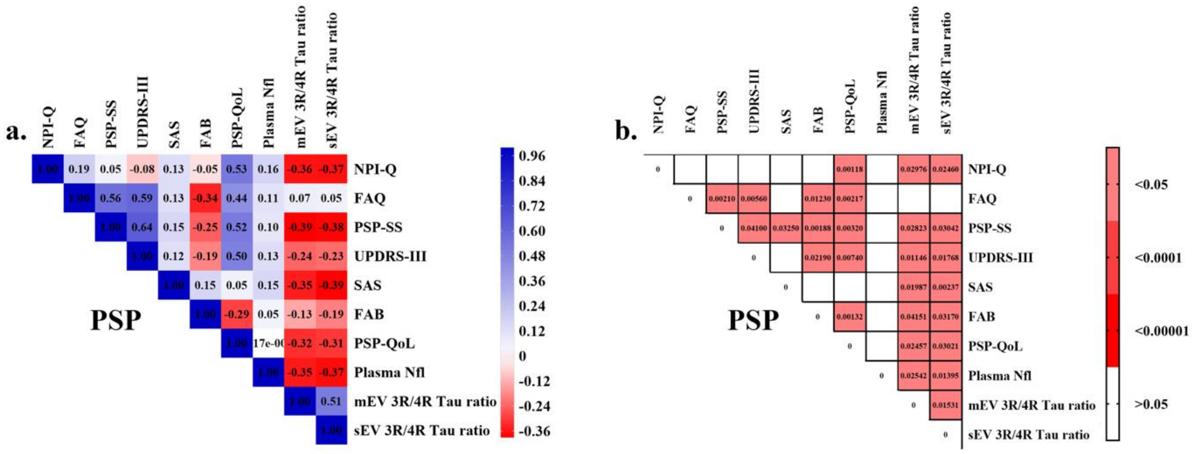

Suppl. Figure 8. Correlation matrix. Results of two-sided Spearman correlations between different clinical and neuropsychological measures of disease severity and sEV and mEV 3R/4R Tau ratio, visualized by plotting strength of correlation (*r*) as a heat map along with *p* values (right).

Supplementary Figure 9: DESCRIBE subcohort 2

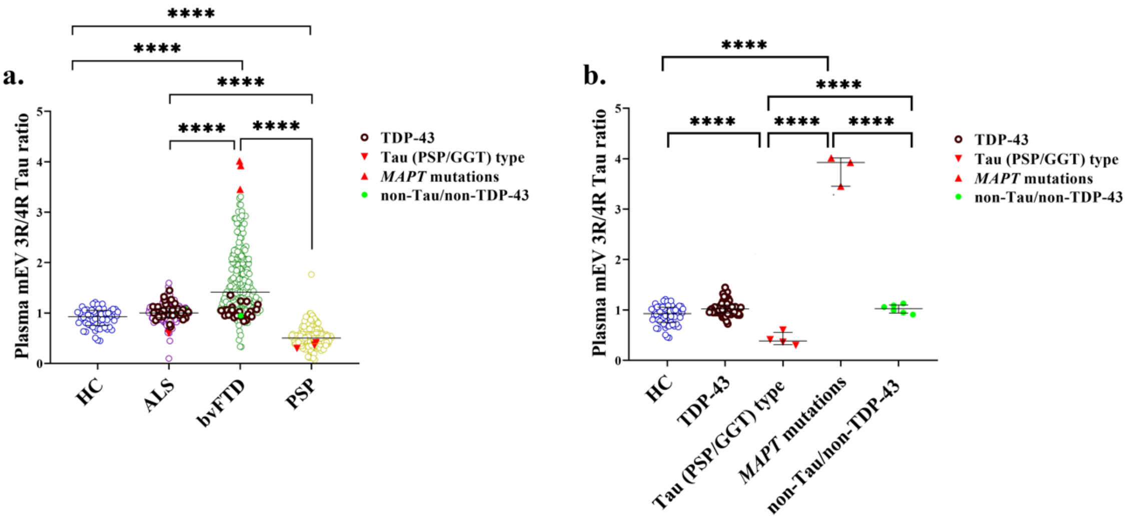

Suppl. Figure 9. Plasma mEV 3R/4R Tau ratio in genetic (n=37) or autopsy confirmed (n=31) cases from DESCRIBE subcohort 2 Total number of individual cases n= 63, 5 of these cases had both, genetic and neuropathological diagnosis. **TDP-43 pathology group**: bvFTD [*C9orf72* (n=13), *GRN* (n=4), *VCP* (n=4), *TBK1* (n=2)]; ALS [*C9orf72* (n=5)]; neuropathological diagnosis [FTLD-TDP (n=1)]; ALS-TDP (n=17), ALS-FTLD TDP (n=6)]. **PSP/GGT-type Tau pathology group**: neuropathological diagnosis [(PSP-Tau (n=3); FTLD-Tau GGT type (n=1)]. **bvFTD *MAPT* mutations**: [*MAPT P301L* (n=1), *MAPT P364S* (n=1), *MAPT IVS10+16C>T* (n=1]. **Non-Tau/non-TDP-43 pathology group**: ALS [*SOD-1* (n=2); *FUS* (n=2); *CHCHD10* (n=1)]; bvFTD [*CHCHD10* (n=1)]. **(a)** mEV 3R/4R Tau ratios in the different pathology groups, stratified by clinical diagnosis (HC vs. bvFTD *p*=0.0000052, HC vs. PSP *p*=0.0000012, ALS vs. bvFTD *p*=0.0000097, ALS vs. PSP *p*=0.0000056, bvFTD vs. PSP *p*=0.0000041; ****p<0.00001). **(b)** mEV 3R/4R Tau ratios of the different pathology groups, independent from clinical diagnostic group. The long horizontal line represents the median and the short horizontal lines represent the inter-quartile range (IQR). Kruskal-Wallis test with Dunn's correction for multiple comparisons. HC vs. Tau (PSP/GGT) type *p*=0.0000058, HC vs. *MAPT* mutations *p*=0.0000069, Tau (PSP/GGT) type vs. *MAPT* mutations *p*=0.0000084, Tau(PSP/GGT) type vs. non-Tau/non-TDP-43 *p*=0.0000099, *MAPT* mutations vs. non-Tau/non-TDP-43 *p*=0.0000032;**** *p*<0.00001). mEV 3R/4R Tau ratios in the different groups. HC: median mEV 0.90, IQR[0.75-1.05]; TDP-43 group: median mEV 1.03, IQR[0.93-1.09]; non-TDP-43/non-Tau group: median mEV 1.03, IQR[0.95-1.11]. Neuropathologically confirmed PSP/GGT-type 4R Tau pathology group: median mEV 0.41, IQR[0.31-0.55]. Patients with Tau pathology in the bvFTD group (*MAPT* mutation carriers): median mEV 3.79, IQR[3.45-4.01].

Supplementary Figure 10

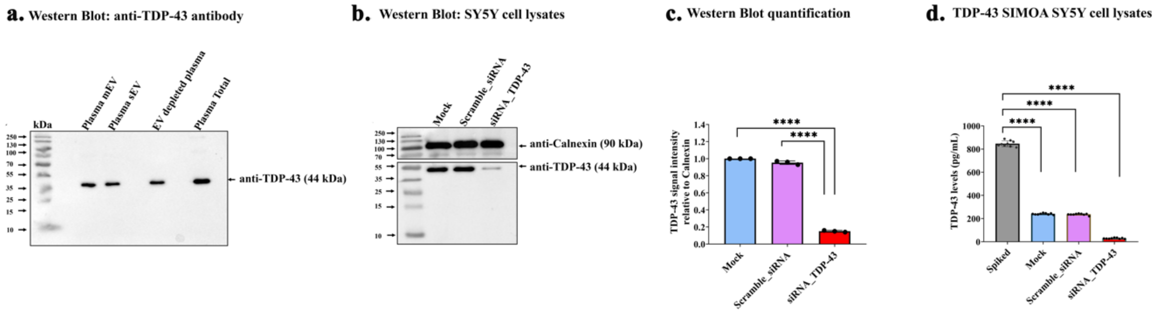

Suppl. Figure 10. Presence of TDP-43 in plasma EVs (a) Western blot of (lanes from left to right) plasma mEVs, sEVs, EV-depleted plasma, and total plasma with an antibody directed against TDP-43. **(b)** Western blot of SY5Y cell lysates (lanes from left to right) after mock transfection, scrambled siRNA, and TDP-43 siRNA transfection. Upper panel probed with anti-Calnexin antibody as a loading control, lower panel probed with anti-TDP-43 antibody (n= 3 independent experiments). **(c)** Western blot quantification: TDP-43 signal (optical density) normalized to Calnexin. Mock, scrambled, and TDP-43 siRNA transfected SY5Y cell lysates. (n= 3 independent experiments). Kruskal-Wallis test with Dunn's correction for multiple comparisons. ns=non-significant, mock vs. TDP-43 siRNA *p*=0.0000063, scrambled vs. TDP-43 siRNA; *p*=0.0000033; **** *p*<0.00001). **(d)** SIMOA assay quantification of SY5Y cell lysates (from left to right): spiked with recombinant TDP-43, mock, scrambled, and TDP-43 siRNA transfected. (n= 9 independent experiments). Kruskal-Wallis test with Dunn's correction for multiple comparisons. ns=non-significant, TDP-43 spike vs. mock *p*=0.0000027, TDP-43 spike vs. scrambled siRNA *p*=0.0000048, TDP-43 spike vs. scrambled siRNA *p*=0.0000015; **** *p*<0.00001.

Supplementary Figure 11: DESCRIBE subcohort 2

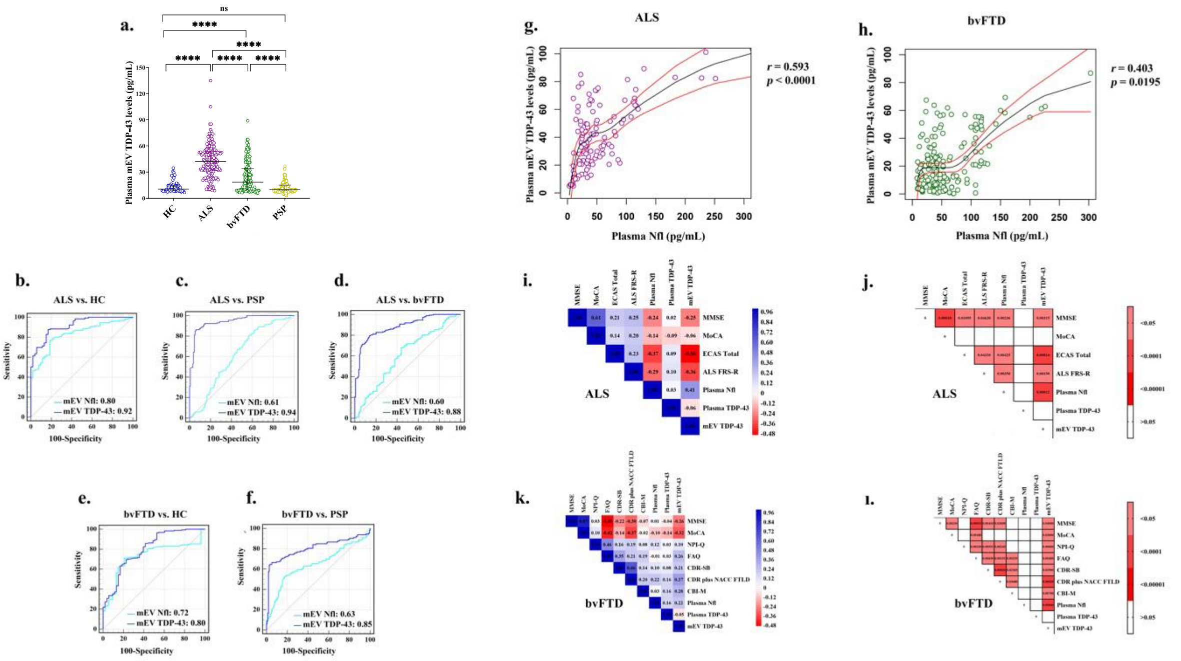

Suppl. Figure 11. Plasma mEV TDP-43 levels in DESCRIBE subcohort 2. Biologically independent samples: HC n= 56, ALS n=165, bvFTD n= 179, PSP n=163. **(a)** Plasma mEV TDP-43 in the different diagnostic groups. Horizontal lines: median and inter-quartile range (IQR). Kruskal-Wallis test with Dunn's correction for multiple comparisons (HC vs. ALS *p*=0.0000028, HC vs. bvFTD *p*=0.0000054, ALS vs. bvFTD *p*=0.0000043, ALS vs. PSP *p*=0.0000012, bvFTD vs. PSP *p*=0.0000093; **** *p*<0.00001). **(b-f)** Receiver Operating Characteristic (ROC) curve for mEV TDP-43 (red) and plasma NfL (blue): **(b)** ALS vs. HC **(c)** ALS vs. PSP **(d)** ALS vs. bvFTD **(e)** bvFTD vs. HC **(f)** bvFTD vs. PSP. The long horizontal line represents the median and the short horizontal lines represent the inter-quartile range (IQR). Kruskal-Wallis test with Dunn's correction for multiple comparisons. **** *p*<0.00001. **(g-h)** Two-sided Spearman correlation analysis of associations between mEV 3R/4R ratio and plasma Nfl levels and monotonic regression splines within **(g)** ALS (*p*=0.000016) and **(h)** bvFTD diagnostic groups. **(i-l)** Correlation matrix depicting results of Spearman correlations, visualized by plotting strength of correlation (*r*) as a heat map along with *p*-values (right). **(i, j)** ALS and **(k, l)** bvFTD.

Supplementary Figure 12: DESCRIBE subcohort 2

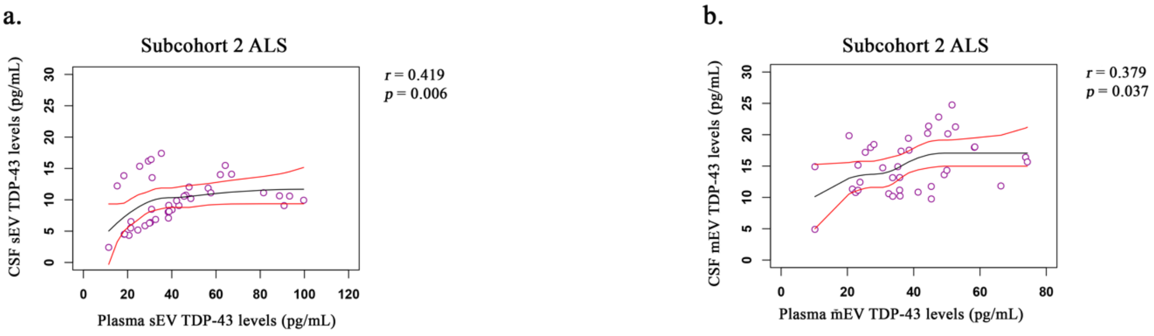

Suppl. Figure 12. Correlation of plasma and CSF TDP-43 in DESCRIBE subcohort 2, ALS group. EVs prepared from 1.5 ml of pooled AD patients CSF revealed TDP-43 levels around the LLOQ and below LLOQ when a CSF volume of 1 ml was used for EV preparation. In contrast, pooled ALS CSF samples showed higher CSF EV TDP-43 concentrations and allowed starting volumes down to 1 ml. We therefore focused on available and corresponding ALS group CSF samples from DESCRIBE subcohort 2. Two-tailed Spearman correlation analysis of associations and monotonic regression splines between **(a)** CSF sEV TDP-43 and plasma sEV TDP-43 (n=30), **(b)** CSF mEV TDP-43 and plasma mEV TDP-43 (n=40). CSF volume for EV preparation: 1 ml. 75.6% (n=41) of the tested CSF samples had EV TDP-43 levels above the detection limit. n=41 biologically independent samples.

Supplementary Figure 13: DESCRIBE subcohort 2

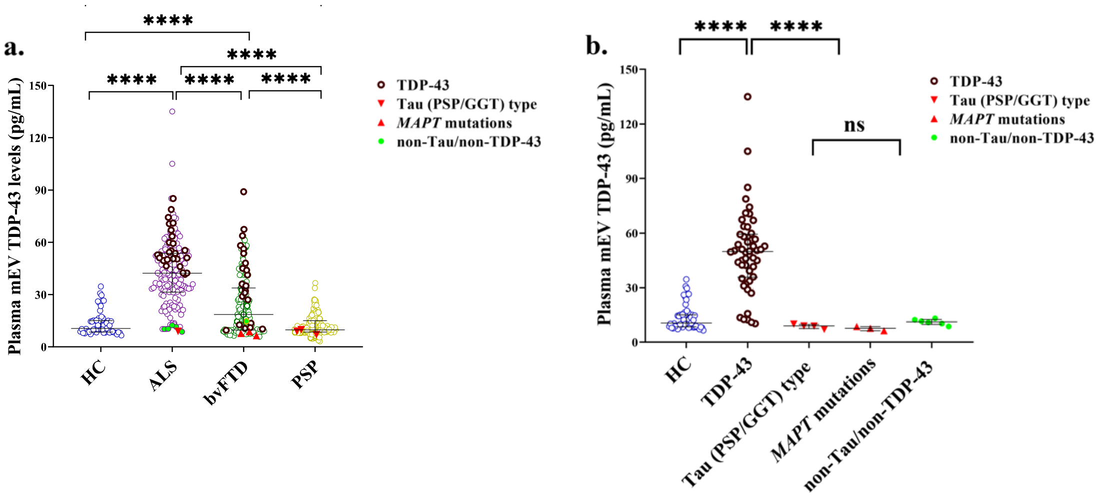

Suppl. Figure 13. TDP-43 in plasma mEV in genetic (n=37) or autopsy confirmed (n=31) cases from DESCRIBE subcohort 2 Total number of individual cases n= 63, 5 of these cases had both, genetic and neuropathological diagnosis. **(a)** plasma mEV TDP-43 in the different pathology groups, stratified by clinical diagnosis (HC vs. ALS *p*=0.0000028, HC vs. bvFTD *p*=0.0000054, ALS vs. bvFTD *p*=0.0000043, ALS vs. PSP *p*=0.0000012, bvFTD vs. PSP *p*=0.0000093; **** *p*<0.00001). **(b)** plasma mEV TDP-43 concentrations in the different pathology groups, independent from clinical diagnostic group. The long horizontal line represents the median and the short horizontal lines represent the inter-quartile range (IQR) (HC vs. TDP-43 *p*=0.0000052, TDP-43 vs. Tau (PSP/GGT) *p*=0.0000091, TDP-43 vs. *MAPT* mutations *p*=0.0000026, TDP-43 vs. non-Tau/non-TDP-43 *p*=0.0000057; **** *p*<0.00001). Kruskal-Wallis test with Dunn's correction for multiple comparisons. **** *p*<0.00001. **TDP-43 pathology group**: bvFTD [*C9orf72* (n=13), *GRN* (n=4), *VCP* (n=4), *TBK1* (n=2)]; ALS [*C9orf72* (n=5)]; neuropathological diagnosis [FTLD-TDP (n=1)]; ALS-TDP (n=17), ALS-FTLD TDP (n=6)]. **PSP/GGT-type Tau pathology group**: neuropathological diagnosis [(PSP-Tau (n=3); FTLD-Tau GGT type (n=1)]. **bvFTD *MAPT* mutations**: [*MAPT P301L* (n=1), *MAPT P364S* (n=1), *MAPT IVS10+16C>T* (n=1]. **Non-Tau/non-TDP-43 pathology group**: ALS [*SOD-1* (n=2); *FUS* (n=2); *CHCHD10* (n=1)]; bvFTD [*CHCHD10* (n=1)]. Median plasma mEV TDP-43 in the different groups. TDP-43 pathology group: 48.74 pg/ml, IQR[34.32-58.70]; PSP/GGT-type Tau pathology group: 2.73 pg/ml, IQR[2.53-3.72]; genetic *MAPT* group: 2.30 pg/ml, IQR[2.23-2.35; non-TDP-43/non-Tau pathology group: 10.74 pg/ml, IQR[9.45-12.36].

Supplementary Figure 14: DESCRIBE subcohort 2

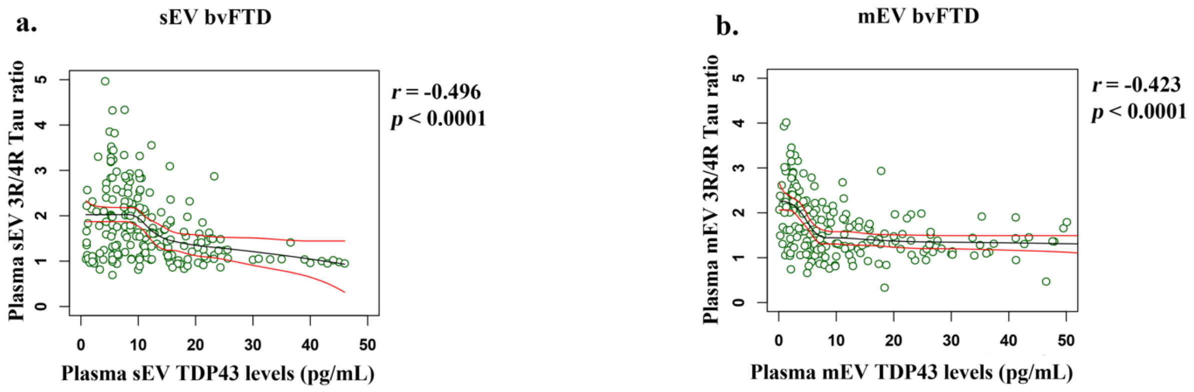

Suppl. Figure 14. Correlation analysis of plasma EV Tau ratio and TDP-43 in DESCRIBE subcohort 2, bvFTD group (a) Two-sided Spearman correlation analysis and monotonic regression splines of associations between plasma sEV 3R/4R Tau ratio and plasma sEV TDP-43 levels (*p*=0.000019). **(b)** Two-sided Spearman correlation analysis and monotonic regression splines of associations between plasma mEV 3R/4R Tau ratio and plasma mEV TDP-43 levels (*p*=0.000049); n=179 biologically independent samples.

Supplementary Figure 15: DESCRIBE subcohort 2

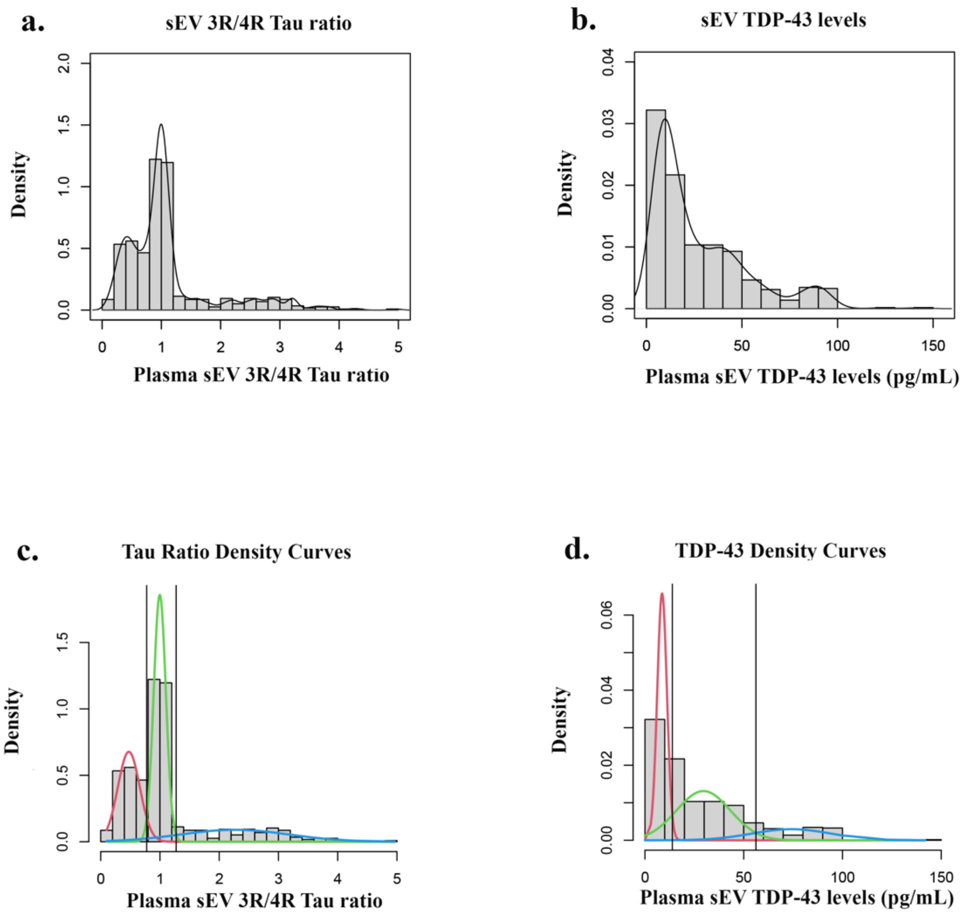

Suppl. Figure 15. Definition of cut-offs by Gaussian mixture modelling. (a,b) Distributions of raw data, plasma sEV 3R/4R Tau, and plasma sEV TDP-43 in **DESCRIBE subcohort 2**. **(c,d)** Data with an estimated mixture of normals. Vertical lines indicate the intersections of the normal mixture components which were defined as cut-off values.

Supplementary Figure 16: Sant Pau cohort

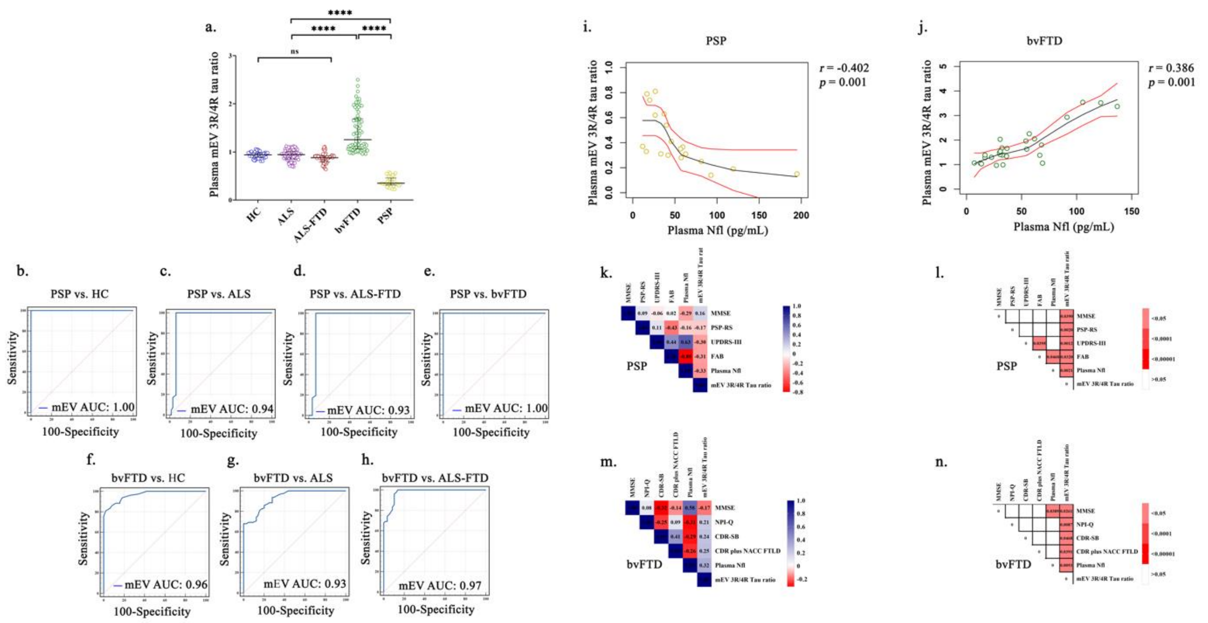

Suppl. Figure 16. Plasma mEV 3R/4R Tau ratios in the Sant Pau cohort (a) Biologically independent samples: HC n= 50, ALS n=65, ALS-FTD n=58, bvFTD n=50 (+23 mutation carriers), PSP n=41). The long horizontal line represents the median and the short horizontal lines represent the inter-quartile range (IQR). Kruskal-Wallis test with Dunn's correction for multiple comparisons (HC vs. bvFTD *p*=0.0000056, HC vs. PSP *p*=0.0000027, ALS vs. bvFTD *p*=0.0000067, ALS vs. PSP *p*=0.0000018, ALS-FTD vs. bvFTD *p*=0.0000061, ALS-FTD vs. PSP *p*=0.0000039, bvFTD vs. PSP *p*= 0.0000057; **** *p*<0.00001). **(b-h)** Receiver Operating Characteristic (ROC) curve for plasma mEV 3R/4R Tau ratio **(b)** PSP vs. HC **(c)** PSP vs. ALS **(d)** PSP vs. ALS-FTD **(e)** PSP vs. bvFTD **(f)** bvFTD vs. HC **(g)** bvFTD vs. ALS **(h)** bvFTD vs. ALS-FTD. **(i-j)** Two-sided Spearman correlation analysis of associations and monotonic regression splines between plasma mEV 3R/4R Tau ratios and plasma Nfl levels within **(i)** PSP and **(j)** bvFTD diagnostic groups. **(k-n)** Correlation matrix depicting results of two-sided Spearman correlations, visualized by plotting strength of correlation (*r*) as a heat map (left) along with *p*-values (right). **(k, l)** PSP and **(m, n)** bvFTD.

Supplementary Figure 17: Sant Pau cohort

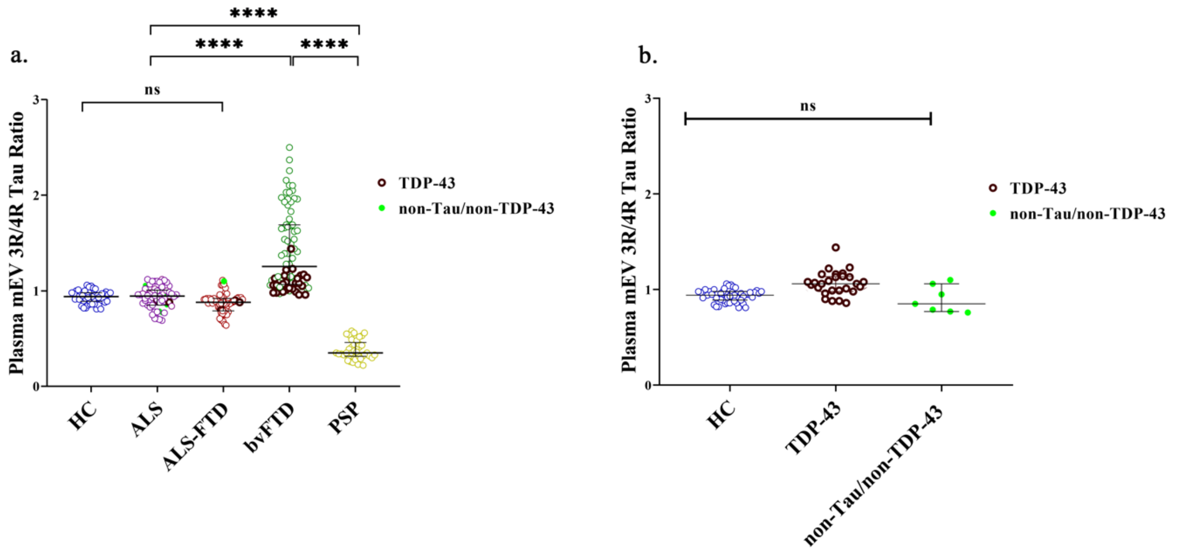

Suppl. Figure 17. Sant Pau cohort, plasma mEV 3R/4R Tau ratios in genetic cases. TDP-43 pathology (brown circles), non-Tau/non-TDP-43 pathology (filled green circles) **(a)** stratified by the different diagnostic groups (HC vs. bvFTD *p*=0.0000056, HC vs. PSP *p*=0.0000027, ALS vs. bvFTD *p*=0.0000067, ALS vs. PSP *p*=0.0000018, ALS-FTD vs. bvFTD *p*=0.0000061, ALS-FTD vs. PSP *p*=0.0000039, bvFTD vs. PSP *p*=0.0000057; **** *p*<0.00001) **(b)** independent from diagnostic groups. The long horizontal line represents the median and the short horizontal lines represent the inter-quartile range (IQR). Kruskal-Wallis test with Dunn's correction for multiple comparisons (HC vs. TDP-43 *p*=0.632, HC vs. non-Tau/non-TDP-43 *p*=0.412, TDP-43 vs. non-Tau/non-TDP-43 *p*=0.256; n.s. not significant). **TDP-43 pathology group**: bvFTD [*C9orf72* (n=12), *GRN* (n=6), *TARDP* (n=1), *VCP* (n=1), *TBK-1* (n=3)]; ALS [*C9orf72* (n=3)]; ALS-FTD *C9orf72* (n=1)]. **Non-Tau/non-TDP-43 pathology group**: ALS [*SOD-1* (n=3); *FUS* (n=3)]; ALS-FTD [*SOD-1* (n=1)]. Plasma mEV 3R/4R Tau ratios. HC: median 0.94, IQR[0.81-1.06]; TDP-43 associated genetic cases group: median 1.03, IQR[0.86-1.44]; non-TDP-43/non-Tau associated genetic cases group: median 0.9, IQR[0.76-1.15]).

Supplementary Figure 18: Sant Pau cohort

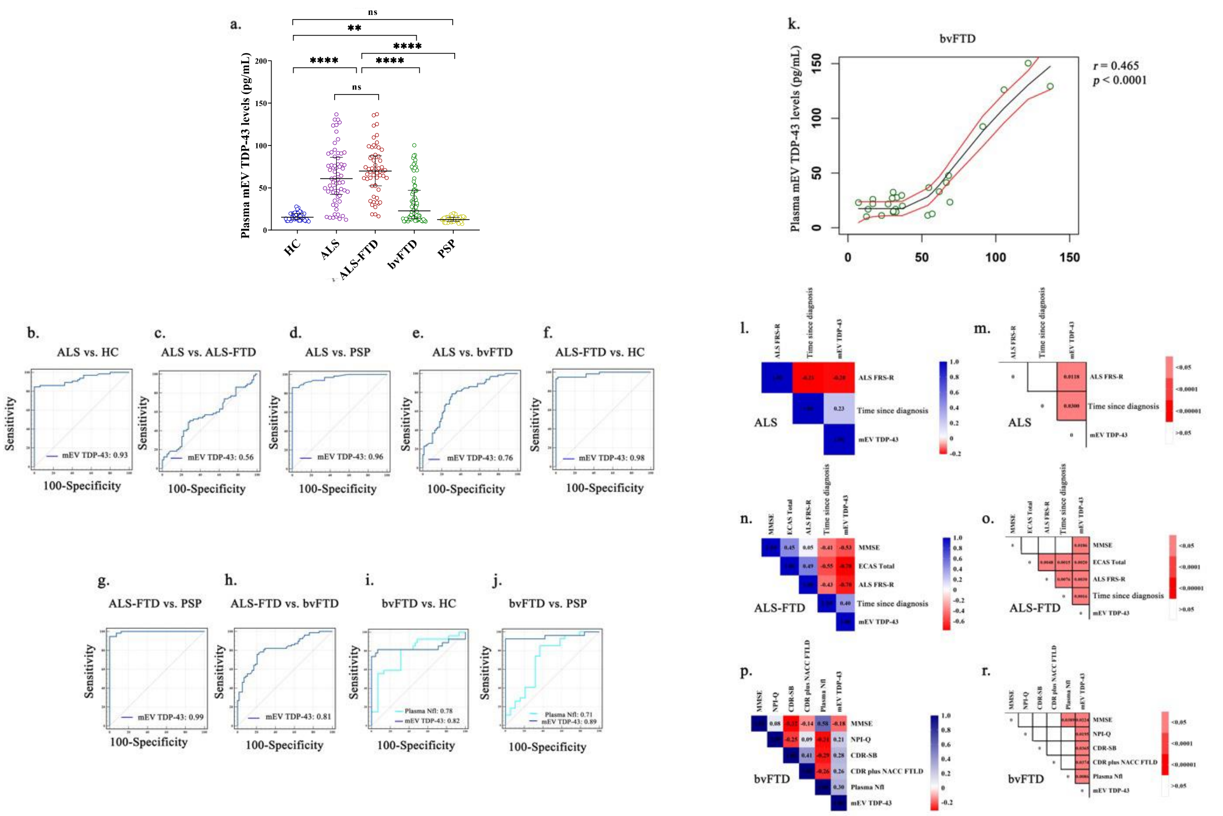

Suppl. Figure 18. **Plasma mEV TDP-43 levels in the different diagnostic groups of Sant Pau cohort**. **(a)** Biologically independent samples: HC n= 50, ALS n=65, ALS-FTD n=58, bvFTD n=50(+23 mutations), PSP n=41). The long horizontal line represents the median and the short horizontal lines represent the inter-quartile range (IQR). Kruskal-Wallis test with Dunn's correction for multiple comparisons (HC vs. ALS *p*=0.0000014, HC vs. ALS-FTD *p*=0.0000033, ALS vs. bvFTD *p*=0.00059, HC vs. PSP *p*=0.763, ALS vs. bvFTD *p*=0.000005, ALS vs. PSP *p*=0.0000062, ALS-FTD vs. bvFTD *p*=0.0000053, ALS-FTD vs. PSP *p*=0.0000042, bvFTD vs. PSP *p*=0.00054; ***p*<0.001, *****p*<0.00001). **(b-j)** Receiver Operating Characteristic (ROC) curve with AUC values for plasma mEV TDP-43: **(b)** ALS vs. HC **(c)** ALS vs. ALS-FTD **(d)** ALS vs. PSP **(e)** ALS vs. bvFTD **(f)** ALS-FTD vs. HC **(g)** ALS-FTD vs. PSP **(h)** ALS-FTD vs. bvFTD **(i)** bvFTD vs. HC **(j)** bvFTD vs. PSP. **(k)** Two-sided Spearman correlation analysis between plasma mEV TDP-43 and plasma Nfl levels and monotonic regression splines in patients with bvFTD (*p*=0.00007). **(l-r)** Correlation matrix depicting results of two-sided Spearman correlations, visualized by plotting strength of correlation (*r*) as a heat map (left) along with *p*-values (right). **(l, m)** ALS, (ALS Functional Rating scale (ALS-FRS) mEV: *r*=-0.200, *p*=0.011) and time since diagnosis/disease duration (mEV: *r*=0.233, *p*=0.029) **(n, o)** ALS-FTD (ALS Functional Rating scale (ALS-FRS) mEV: *r*=-0.699, *p*=0.003) and time since diagnosis/disease duration (mEV: *r*=0.404, *p*=0.001); MMSE (mEV: *r*=-0.533, *p*=0.017) and **(p, r)** bvFTD.

Supplementary Figure 19: Sant Pau cohort

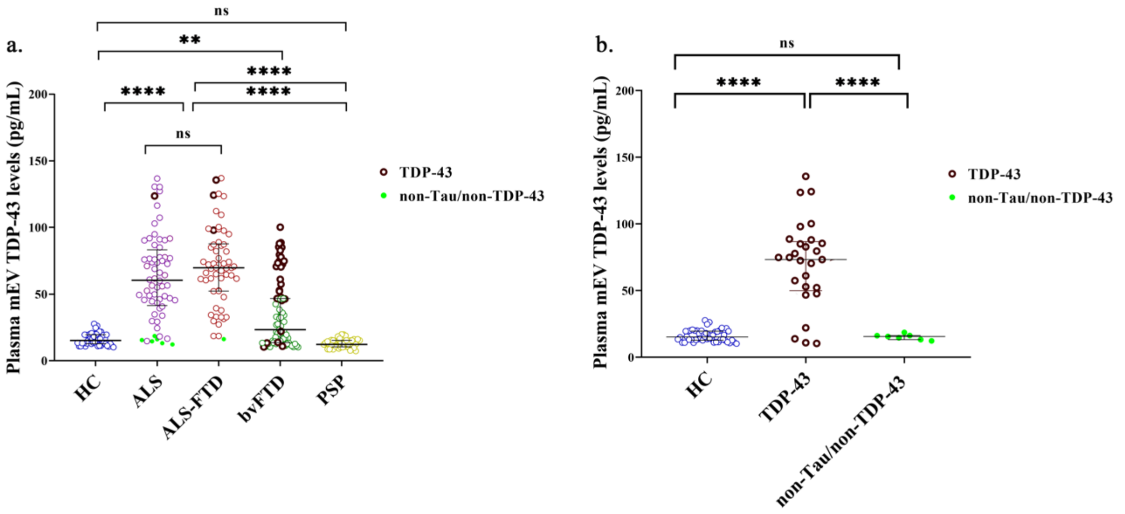

Suppl. Figure 19. Plasma mEV TDP-43 levels in genetic cases of Sant Pau cohort. Cases associated with TDP-43 pathology (brown circles) or non-Tau/non-TDP-43 pathology (filled green circles) **(a)** stratified by the different diagnostic groups (HC vs. ALS *p*=0.0000014, HC vs. ALS-FTD *p*=0.0000033, ALS vs. bvFTD *p*=0.00059, HC vs. PSP *p*=0.763, ALS vs. bvFTD *p*=0.000005, ALS vs. PSP *p*=0.0000062, ALS-FTD vs. bvFTD *p*=0.0000053, ALS-FTD vs. PSP *p*=0.0000042, bvFTD vs. PSP *p*=0.00054; ** p < 0.001, **** *p*<0.00001). **(b)** independent from diagnostic groups. The long horizontal line represents the median and the short horizontal lines represent the inter-quartile range (IQR). Kruskal-Wallis test with Dunn's correction for multiple comparisons (HC vs. TDP-43 *p*=0.0000063, HC vs. non-Tau/non-TDP-43 *p*=0.541, TDP-43 vs. non-Tau/non-TDP-43 *p*=0.0000051; ***p*<0.001, ****p*<0.0001, *****p*<0.00001); n.s. not significant. **TDP-43 pathology group**: bvFTD [*C9orf72* (n=12), *GRN* (n=6), *TARDP* (n=1), *VCP* (n=1), *TBK-1* (n=3)]; ALS [*C9orf72* (n=3)]; ALS-FTD *C9orf72* (n=1)]. **Non-Tau/non-TDP-43 pathology group**: ALS [*SOD-1* (n=3); *FUS* (n=3)]; ALS-FTD [*SOD-1* (n=1)]. Plasma mEV TDP-43 levels. TDP-43 pathology group: median mEV: 72.16 pg/ml, IQR[10.35-135.6]), non-Tau/non-TDP-43 group: median mEV: 15.17 pg/ml, IQR[12.30-18.63]), HC: median mEV: 15.20 pg/ml, IQR[10.23-27.73].

Supplementary Figure 20: Sant Pau cohort

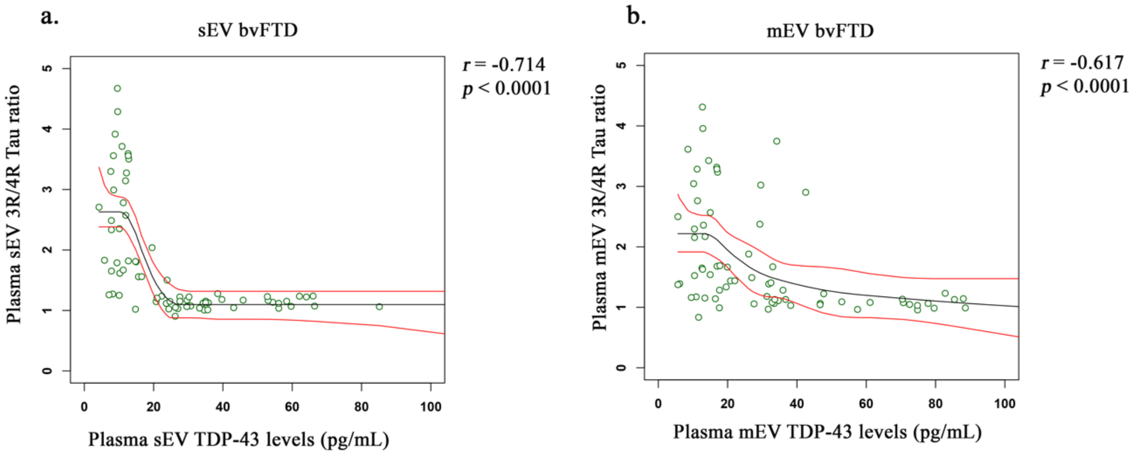

Suppl. Figure 20. Correlation analysis of plasma EV Tau ratio and TDP-43 in the Sant Pau cohort bvFTD group (sporadic and genetic cases) (a) Two-sided Spearman correlation analysis and monotonic regression splines of associations between plasma sEV 3R/4R Tau ratio and plasma sEV TDP-43 levels (*p*=0.000021). **(b)** Two-sided Spearman correlation analysis and monotonic regression splines of associations between plasma mEV 3R/4R Tau ratio and plasma mEV TDP-43 levels (*p*=0.000036); n=73 biologically independent samples.

Supplementary Figure 21: Sant Pau cohort

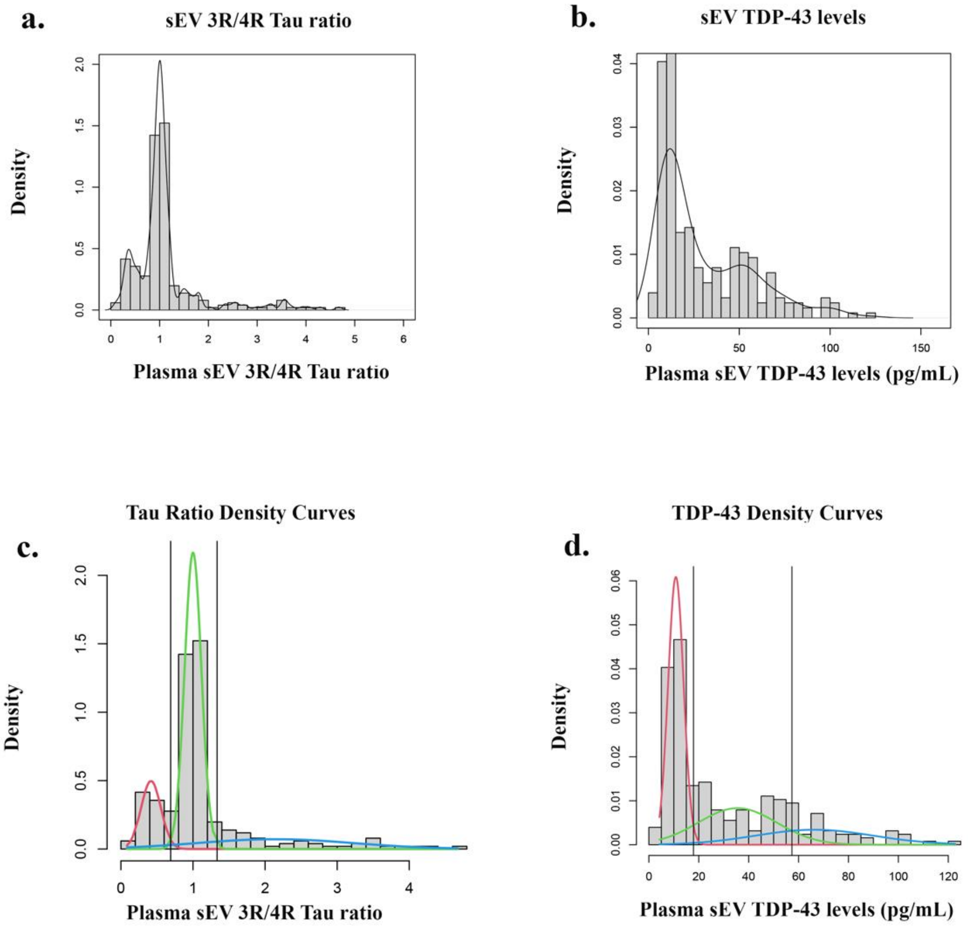

Suppl. Figure 21. Determination of cut-off values in the Sant Pau cohort (a,b) distributions of raw data, **(a)** plasma sEV 3R/4R Tau ratio, and **(b)** plasma sEV TDP-43. **(c,d)** data with an estimated mixture of normal. **(c)** sEV Tau ratio, **(d)** sEV TDP-43. Vertical lines indicate the intersections of the normal mixture components which were defined as cut-off values. Distribution of sEV Tau ratios and TDP-43 was best approximated by three normal Gaussian distributions. Plasma sEV 3R/4R Tau ratio: *p*=0.001; sEV TDP-43: *p*=0.001 compared to one (plasma sEV 3R/4R Tau ratio: *p*=0.001; sEV TDP-43: *p*=0.01), two (plasma sEV 3R/4R Tau ratio: *p*=0.001; sEV TDP-43: *p*=0.01) or four Gaussian distributions (plasma sEV 3R/4R Tau ratio: *p*=0.1; sEV TDP-43: *p*=0.1).

Supplementary Figure 22

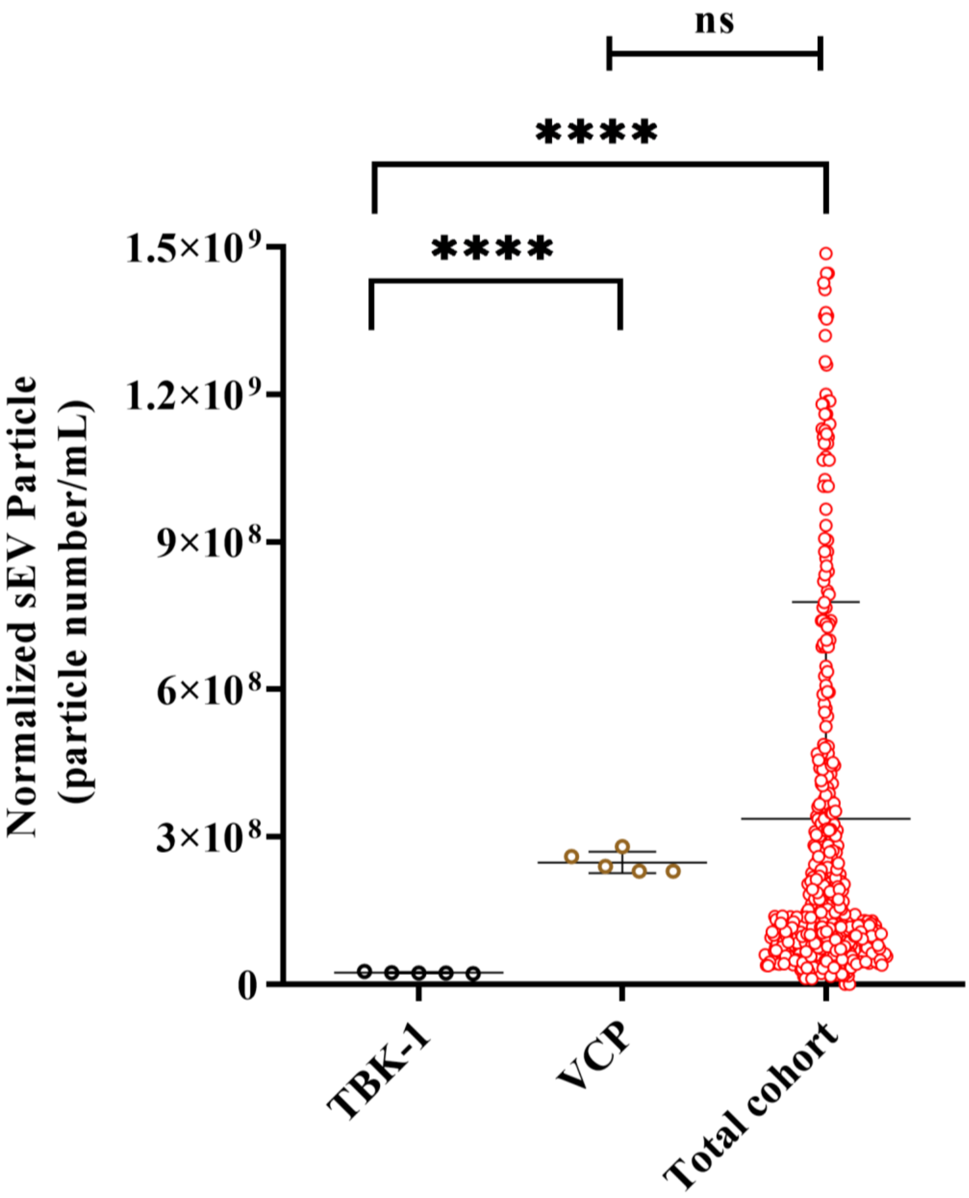

Suppl. Fig. 22. Plasma sEV particle concentrations in *TBK-1* and *VCP* mutation carriers. Plasma sEV concentrations are given as particle numbers/mL in *TBK-1* mutation carriers (black), *VCP* mutation carriers (brown) and all other samples (red). Data were normalized and pooled from DESCRIBE subcohort 2 and Sant Pau cohort. Of note, *VCP* mutation carriers showed plasma EV concentrations which were comparable to the cohorts' mean value. (*TBK-1,* n=5 in DESCRIBE subcohort 2 and Sant Pau cohort) and *VCP* (n=5 in DESCRIBE subcohort 2 and Sant Pau cohort). Total cohort: n= 850 biologically independent samples, sEV particle numbers of DESCRIBE subcohort 2 (n=563) and Sant Pau cohort (n=287). Kruskal-Wallis test followed by Dunn's correction for multiple comparisons. Data

are presented as mean +/- SEM. n.s: non-significant; *TBK-1* vs. *VCP* p=0.0000085, *TBK-1* vs. total cohort p=0.0000065, *VCP* vs. total cohort p = 0.733, ****p < 0.00001.

Supplementary Figure 23: Sant Pau cohort

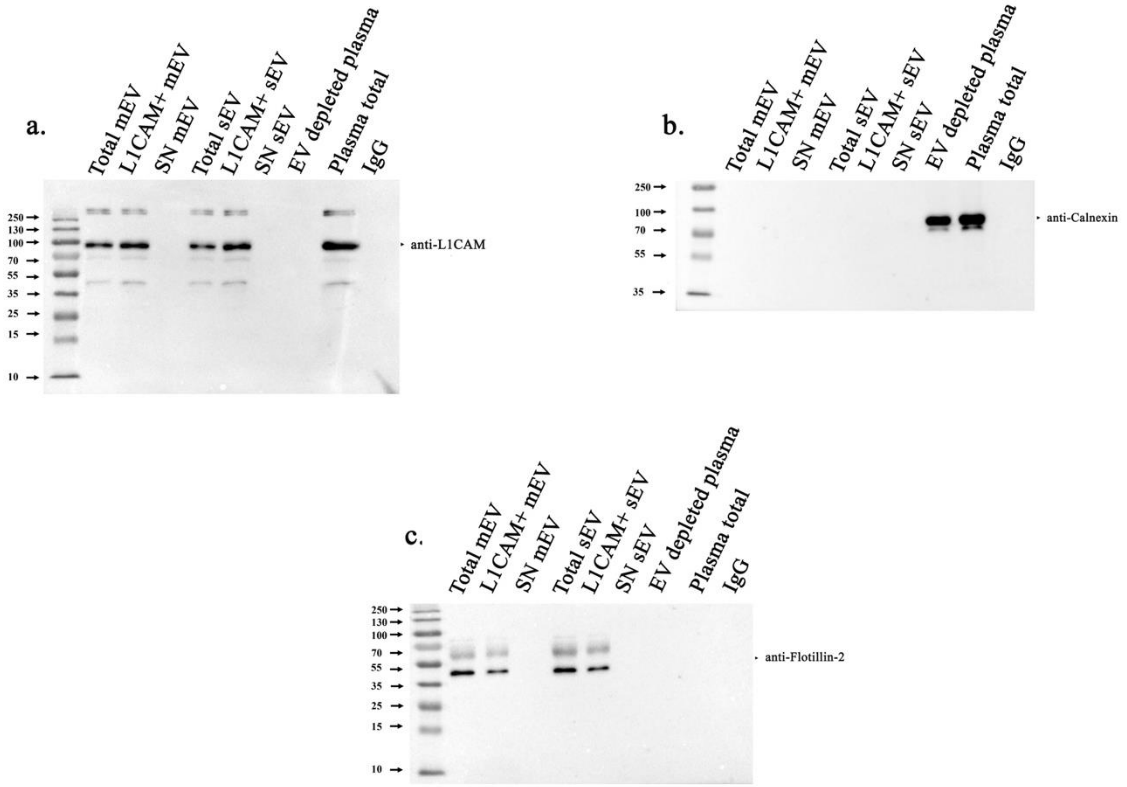

Suppl. Fig. 23. Uncropped Western Blots from Suppl. Fig. 4 a. (a) anti-L1CAM, **(b)** anti-Calnexin**, (c)** anti-Flotillin-2.
