# Supplementary material for: Plasma extracellular vesicle tau and TDP-43 as diagnostic biomarkers in FTD and ALS
Source: Nat Med. 2024 Jun 18;30(6):1771–83. doi: 10.1038/s41591-024-02937-4 (PMC11186765; doi:10.1038/s41591-024-02937-4)
Supplement: Supplementary file 2 — Reporting Summary [file 41591_2024_2937_MOESM2_ESM.pdf]

Corresponding author(s): Anja Schneider
Last updated by author(s): 14.03.2024

Reporting Summary

Nature Portfolio wishes to improve the reproducibility of the work that we publish. This form provides structure for consistency and transparency in reporting. For further information on Nature Portfolio policies, see our Editorial Policies and the Editorial Policy Checklist.

Statistics

For all statistical analyses, confirm that the following items are present in the figure legend, table legend, main text, or Methods section.

n/a	Confirmed	
☐	☒	The exact sample size (*n*) for each experimental group/condition, given as a discrete number and unit of measurement
☐	☒	A statement on whether measurements were taken from distinct samples or whether the same sample was measured repeatedly
☐	☒	The statistical test(s) used AND whether they are one- or two-sided<br>*Only common tests should be described solely by name; describe more complex techniques in the Methods section.*
☐	☒	A description of all covariates tested
☐	☒	A description of any assumptions or corrections, such as tests of normality and adjustment for multiple comparisons
☐	☒	A full description of the statistical parameters including central tendency (e.g. means) or other basic estimates (e.g. regression coefficient) AND variation (e.g. standard deviation) or associated estimates of uncertainty (e.g. confidence intervals)
☐	☒	For null hypothesis testing, the test statistic (e.g. *F*, *t*, *r*) with confidence intervals, effect sizes, degrees of freedom and *P* value noted<br>*Give P values as exact values whenever suitable.*
☒	☐	For Bayesian analysis, information on the choice of priors and Markov chain Monte Carlo settings
☒	☐	For hierarchical and complex designs, identification of the appropriate level for tests and full reporting of outcomes
☐	☒	Estimates of effect sizes (e.g. Cohen's *d*, Pearson's *r*), indicating how they were calculated

Our web collection on statistics for biologists contains articles on many of the points above.

Software and code

Policy information about availability of computer code

Data collection	No code involved in data collection
Data analysis	No unpublished software.<br><br>Statistical analysis<br>Statistical analysis and data visualization were performed using Prism 7 (GraphPad Software Inc., La Jolla, CA, USA), IBM SPSS Statistics 21 (IBM Corporation, Armonk, NY, USA), R studio 3.2.1 (R Foundation for Statistical Computing, Institute for Statistics and Mathematics, Wirtschaftsuniversität Wien, Vienna, Austria), MedCalc software version 22.021 and HD-X analyzer, software version 3.1 (Quanterix, Billerica, MA, USA). The statistical tests were two-tailed and values with p <0.05 were considered significant. Comparisons of marker levels were performed using Kruskal-Wallis tests followed by Dunn's correction for multiple comparisons due to non- Gaussian distributions. Normal distribution assumption was assessed based on visual inspection of histograms and Kolmogorov-Smirnov tests. To assess the link between EV marker and clinical scales as well as plasma NfL, Spearman correlations were used. To illustrate associations between plasma Nfl and plasma EV 3R/4R Tau ratio, plasma NfL and plasma EV /TDP-43, as well as plasma EV 3R/4R Tau and plasma EV TDP-43 (Fig. 1,2,4, Suppl. Fig.S,7,11,14), monotonic regression splines (using the "cgam" function from R package "splines") were modeled. Notably, potential confounders (i.e. age, sex and disease duration) showed no influence on plasma biomarker levels (Suppl. Tables 3, 7). We therefore used the non-parametric tests described above with covariate adjustment to account for violations of normal distribution assumptions and non-linear relationships.<br>MedCalc was used for computation and comparison of ROC curves (with the method of Hanley&McNeil[122])(standard error (SE), 95% confidence interval (CI) for the difference, and p-value) as well as for calculation of sensitivity and specificity. Precision recall curves, area under the precision recall curve (AUPRC) and confidence intervals were calculated using the R code from Boyd et al. (2013)[123] and published prevalence estimates for the different diagnoses (PSP[124], ALS[125], bvFTD[126]).

The cut-off values of 3R/4R Tau ratio and TDP-43 levels were defined with Gaussian mixture modeling using the R 3.2.1 mix tools package as previously described by Bertens et al. [61]. First, the R boot.comp function was used to determine the number of distributions that fitted best to the data. Next, we defined data-driven cut-offs as the point where the lines of fitted normal distributions crossed each other. Specifically, we derived three normal distributions (as suggested by bootstrapping) and determined the intersection of the middle normal distribution with the two more extreme distributions. We computed sensitivity and specificity based on the cut-offs of plasma sEV 3R/4R Tau ratio and TDP-43 levels as determined by mixture modelling.

For manuscripts utilizing custom algorithms or software that are central to the research but not yet described in published literature, software must be made available to editors and reviewers. We strongly encourage code deposition in a community repository (e.g. GitHub). See the Nature Portfolio guidelines for submitting code & software for further information.

Data

Policy information about availability of data

All manuscripts must include a data availability statement. This statement should provide the following information, where applicable:

- Accession codes, unique identifiers, or web links for publicly available datasets
- A description of any restrictions on data availability
- For clinical datasets or third party data, please ensure that the statement adheres to our policy

The data that support the findings of this study are available from the authors but restrictions apply, and hence not available publicly due to ethics approval regulations/data protection. Data are, however, available from the authors upon reasonable request and with permission from the cohorts' steering committees (contact for and information on data access: anja.schneider@dzne.de). Expected turnover times for data applications is 3 months.

Research involving human participants, their data, or biological material

Policy information about studies with human participants or human data. See also policy information about sex, gender (identity/presentation), and sexual orientation and race, ethnicity and racism.

Reporting on sex and gender

We report on sex in a demographics table. We tested for correlation of our data with sex and no correlation was found.

Reporting on race, ethnicity, or other socially relevant groupings

We do not report on race, ethnicity or socially relevant groupings. Information on sex was obtained by the study physicians (database entry). Study physicians obtained this information from patients, patients charts, name or in case of ambiguity by asking patients and or caregivers.

Population characteristics

Detailed information is found in Extended Data Tables 1&2

Recruitment

Patient samples
The DZNE Clinical Registry Study of Neurodegenerative Diseases (DESCRIBE) cohort is a multicentric, longitudinal observational study conducted by the German Center for Neurodegenerative Diseases (DZNE) and its clinical sites. It recruits patients with different neurodegenerative conditions, including ALS, bvFTD and PSP. Recruitment of these patients is described in more detail below. The multicenter, longitudinal Degeneration Controls and Relatives cohort (DANCER) serves to provide healthy controls for all DESCRIBE subcohorts. After written informed consent (University of Bonn Ethics Board statement 311/14) all participants undergo baseline and annual follow-up visits with clinical and neurological examination, cognitive assessments, 3T magnetic resonance imaging (MRI), blood and CSF sampling following identical standard operating procedures (SOPs). Patients with Alzheimer's Disease (AD) dementia were recruited as part of the DESCRIBE cohort, following the National Institutes of Aging- Alzheimer's Association (NIA-AA) diagnosis criteria90 and confirmed by positive CSF amyloid-beta, total Tau and p-Tau181 status.

Patients were recruited at the different DZNE clinical cooperation units from specialized outpatient clinics. Patients were referred to these clinics by general physicians, neurologists or psychiatrists. DANCER participants were recruited by advertisement (DZNE website, flyers) from the general population. Although we included patients at all disease stages at all levels of cognitive or motor impairment, there could be a selection bias since patients with more severe impairment or their caregivers may not be willing to participate. Thus, our cohort may underrepresent more progressed disease stages. Since the study focuses on early diagnostic markers and since we also correlated results with clinical scales of disease severity, this should not have affected our findings.

The DESCRIBE ALS cohort
ALS patients were diagnosed according to the revised El-Escorial-Criteria91. Different motor phenotypes of ALS were classified as classical ALS, progressive bulbar paresis, flail arm, flail leg, progressive muscular atrophy (PMA), primary lateral sclerosis (PLS) or genetic ALS. Participants were clinically characterized using the Amyotrophic Lateral Sclerosis Functional Rating Scale-revised (ALS-FRS-R)92. The Edinburgh Cognitive and Behavioral ALS Screen (ECAS)93 served as an additional test to identify cognitive and behavioral impairment. ALS patients with cognitive impairment (ALSci), ALS with behavioral impairment (ALSbi), ALS with cognitive and behavioral impairment (ALScbi) and ALS with frontotemporal dementia (ALS-FTD) following the Strong criteria94 and genetic ALS with a pathogenic FTD mutation, additionally underwent the assessments of the DESCRIBE FTD cohort (see below).

The DESCRIBE FTD cohort
Patients with bvFTD were diagnosed according to the revised Rascovsky criteria9S by an experienced multidisciplinary team of neurologists, psychiatrists and neuropsychologists and under consideration of MRI images and CSF data, when available. Neuropsychological assessments included Mini Mental State Examination Test (MMSE), the Montreal Cognitive Assessment

(MoCA)41, Free and Cued Selective Reminding Test (FCSRT)96, the Neuropsychological battery of the Consortium to Establish a Registry for Alzheimer's Disease (CERAD) Plus test97 including Trail Making Tests A and B and the mini-Social cognition & Emotional Assessment (Mini-SEA)98 test. Psychiatric scales included Geriatric Depression Scale (GDS)99, the brief questionnaire of the Neuropsychiatric Interview (NPI-Q)S3, and the functional scales CDR-SB, CDR plus NACC FTLD, Functional Activities Questionnaire (FAQ)S0, and a modification of the revised Cambridge Behavior Inventory (CBI-R)54, the CBI-M.
Patients with semantic variant PPA (svPPA) were diagnosed according to Gordon-Tempini criteria100. Baseline assessment of patients with PPA additionally included a modified version of the Camel and Cactus test101, the visual form of the Sentence Comprehension Test (SECT-V)102, the Sentence Repetition Test from the Aachen Aphasia Test103, hierarchical word lists104 and the Repeat and Point Test105.

The DESCRIBE PSP cohort
The cohort design is summarized in106. Diagnosis of PSP was based on the National Institute of Neurological Disorders and Stroke and the Society for PSP (NIND-SPSP) criteria107 for participants recruited before 2017, and on the Movement Disorder Society (MDS-PSP) diagnostic criteria108 for participants recruited after 2017. Participants were clinically phenotyped by PSP rating scale (PSP-RS)42, PSP staging system (PSP-SS)109, PSP quality of life scale (PSP-QoL)48, PSP-clinical deficits scale (PSP-CDS)43, Schwab and England disability scale (SEADL)44, MDS-Unified Parkinson's Disability Rating Scale (MDS-UPDRS) Part III46, Starkstein Apathy Scale (SAS)47, Clinical Global Impression Severity Scale (CGI-s)45, GDS99, and MoCA41.

The Healthy Control cohort DANCER
Healthy controls samples were obtained from the Degeneration Controls and Relatives cohort (DANCER) and included 71 participants who, based on neuropsychological testing, neurological and psychiatric examination, do not suffer from a neurodegenerative disease. Participants additionally underwent MR imaging. The neuropsychological test battery follows the same protocol and includes all assessments as the one used for participants of the DESCRIBE FTD cohort. Participants undergo an annual follow-up as well as genetic testing at baseline (see below). Relatives with a known pathogenic FTD-ALS mutation were excluded as controls.

The Sant Pau cohort
Patients with ALS were prospectively recruited from the Motor Neuron Disease Clinic at Hospital de la Santa Creu i Sant Pau. We included patients categorized as probable laboratory-supported, or definite ALS according to El Escorial revised criteria119. ALSFRS-R in its Spanish version120 was systematically assessed at the time of sample acquisition. Unimpaired healthy controls, bvFTD and PSP patients were recruited at the Sant Pau Memory Unit and include individuals from the Sant Pau Initiative on Neurodegeneration multimodal biomarker cohort. ALS-FTD patients were recruited by Sant Apu Memory Unit and Motor Neuron Disease Clinic. Information about clinical and neuropsychological assessments and sample processing have been previously described in detail64. Plasma samples were obtained using the same SOP. All patient samples (ALS, ALS-FTD, bvFTD and PSP) were screened for the presence of a pathogenic repeat expansion mutation in C9orf72. In addition, patients with ALS were tested for mutations in ALS, FTD and AD causing genes using a gene panel. bvFTD and PSP patients underwent whole exome sequencing. In total, pathogenic mutations were found in C9orf72 (n=16), GRN (n=6), SOD1 (n=4), TBK1 (n=3), FUS (n=3), TARDBP (n=1), VCP (n=1). This study was approved by the Hospital de la Santa Creu i Sant Pau Ethics Committee).

Ethics oversight

University of Bonn Ethics Board statement 311/14
Sant Pau Ethics Committee
The DESCRIBE and DANCER cohort studies were approved by University of Bonn Ethics Board, statement 311/14. The Sant Pau cohort was approved by the Sant Pau Ethics Committee.

Note that full information on the approval of the study protocol must also be provided in the manuscript.

Field-specific reporting

Please select the one below that is the best fit for your research. If you are not sure, read the appropriate sections before making your selection.

☒ Life sciences ☐ Behavioural & social sciences ☐ Ecological, evolutionary & environmental sciences

For a reference copy of the document with all sections, see nature.com/documents/nr-reporting-summary-flat.pdf

Life sciences study design

All studies must disclose on these points even when the disclosure is negative.

Sample size

DESCRIBE: subcohort 1 was a pilot study. Subcohort 2: we asked for all available samples and received the given numbers, resulting in unequal group sizes. No power calculation was performed. Sant Pau: sample sizes were chosen based on available sample numbers and with the aim of similar numbers per diagostic groups.

DESCRIBE: We applied for all available samples from the DZNE DESCRIBE cohort with a diagnosis of bvFTD, for 100 samples with ALS and for 125 with PSP and 75 healthy controls. All bvFTD samples were asked for because of the scope of this study to detect TDP-43 and Tau pathology in this group. PSP was applied for as a 4R Tau control cohort, ALS as a TDP-43 control cohort. Sample numbers were determined by availability of samples.
Sant Pau: we applied for n=50 with diagnosis of ALS, ALS-FTD, bvFTD, PSP, healthy controls and as many as possible genetically confirmed cases.

Data exclusions	Modified version of the Cambridge Behavioural Inventory (CBI-M)<br>In the DESCRIBE-FTD cohort, we used a modified 50-item version of the CBI-R. Before including this new version into the analyses, we conducted a principal component analysis (PCA) with varimax rotation to confirm the theoretical factor structure. Participants with over 20% missing rate over all items were removed. In addition, one item with over 20% missing rate across all participants was excluded. Four further items were excluded due to the low factor loadings and cross-loadings. Therefore, the modified version (CBI-M) resulted in 45 items with 12 new items and 33 items from the CBI-R. The total CBI-M score was calculated by the mean of all available item scores for each bvFTD participant.<br>Plasma: 1 case was excluded from subcohort 1 as 4R Tau was not measurable. No other values/samples were excluded.<br>CSF: Several cases had to be excluded from subcohort 1 CSF as 4R Tau was not measurable. The number of excluded cases is given in the manuscript.
Replication	We replicated our findings on Tau ratio in a second subset of the DZNE DESCRIBE cohort (subcohort 2). Measurements of the DESCRIBE cohort samples were done once (singletons) due to the limited availability of sample volume. Findings were reproduced in the independent Sant Pau cohort for both tau and TDP-43.<br>For EV TDP-43 findings in subcohort 2, we determined cut-off values using mixture modelling and tested their sensitivity and specificity in a hold-out data set of confirmed pathology.
Randomization	Samples were randomized on the 96-well plates using sample randomization tool https://olink.com/faq/sample-randomization/
Blinding	Experimenters were blinded to diagnosis

Reporting for specific materials, systems and methods

We require information from authors about some types of materials, experimental systems and methods used in many studies. Here, indicate whether each material, system or method listed is relevant to your study. If you are not sure if a list item applies to your research, read the appropriate section before selecting a response.

Materials & experimental systems

n/a	Involved in the study
☐	☒ Antibodies
☐	☒ Eukaryotic cell lines
☒	☐ Palaeontology and archaeology
☒	☐ Animals and other organisms
☐	☒ Clinical data
☒	☐ Dual use research of concern
☒	☐ Plants

Methods

n/a	Involved in the study
☒	☐ ChIP-seq
☒	☐ Flow cytometry
☒	☐ MRI-based neuroimaging

Antibodies

Antibodies used	Pathology: Tau1 (provided by Drs. Nicholas Kanaan and Lester Binder) and HJ8.5 (provided by Dr. David Holtzman) antibodies against phosphorylated TDP-43 (clone 1D3110), phosphorylated tau (clone AT8, cat no. MN1020,Thermo Fisher), α-synuclein (clone 4D6, cat no. AM05094PU-N, Origene), and beta-Amyloid (clone 4G8, cat no. SIG-39220, Covance).<br><br>Western Blotting (WB), ELISA and MSD: Primary antibodies: Anti-Calnexin (cat no. C4731, Sigma-Aldrich, Darmstadt, Germany, 1:2000 dilution for WB), anti-Flotillin-2 (cat no. 610384, BD Biosciences, San Jose, CA, USA, 1:500 for WB), anti-3R Tau (RD3, cat no. 05-803, Merck,1:600 for MSD, 1:500 for WB), anti-4R Tau (cat no. ab218314, Abcam, 1:300 for ELISA, 1:500 for WB), anti-TDP-43 antibody (cat no. ab305694, Abcam, 1:500 for WB), HT7 (cat no. MN1000B, Thermo Fisher Scientific, epitope residues 159 – 163, 1:300 for MSD/ELISA, 1:500 for WB), biotinylated mouse anti-human CD171 (L1CAM neural adhesion protein) antibody (clone 5G3, eBiosciences, San Diego, CA, USA, cat no. 13171982, Thermo Fisher Scientific,1:500 for WB and IP), biotinylated mouse IgG2 antibody (clone eBM2a, cat no.13472485, Thermo Fisher Scientific),1:500 for WB and IP.<br>Secondary antibodies: HRP anti mouse IgG (Dako, cat number P044701-2, 1:5000), HRP anti rabbit IgG (Dako, cat number P044801-2, 1:5000).<br><br>TDP-43 Simoa advantage kit, cat no. 103293, lot no.503756, Nfl Simoa Advantage kit cat no. 103186, lot no. 503546.
Validation	This validation statement below is provided for 3R tau, 4R tau and TDP-43.<br>Suppl Table 1 and<br>Sensitivity: For the determination of the lower limit of quantification (LLOQ) of each assay, 16 blank samples were measured on one plate. The calibration curves were calculated using a four-parameter logistic curve fit for all assays, which gave the optimal fit. LLOQ was calculated as the concentration corresponding to the 2.S signal standard deviation above the background (zero calibrator) following the guidelines in Andreasson et al.6. Precision: Intra-assay variation (repeatability) was determined by analysis of samples (n=5) in four replicates on one plate. Inter-assay variation (intermediate precision) was measured to determine the variation of analyses between 5 different days. Dilutional linearity: Three different EV samples were used in duplicates to perform the dilution linearity experiments. The dilutional linearity (dilutions 2x, 4x and 8x) was calculated as follows: %Linearity = [observed C *dilution

factor/ previous observed C"previous dilution factor] "100; C = concentration(pg/mL). Recovery: Three different plasma EV samples, measured in duplicates, were spiked with recombinant 3R Tau, 4R Tau, and TDP43 calibrator at three different concentrations (low: 3R and 4R Tau 1,750 pg/mL, TDP-43 1,250 pg/mL; medium: 3R and 4R Tau 3,500 pg/mL, TDP-43 2,500 pg/mL; high: 3R and 4R Tau 7,000 pg/mL, TDP-43 5,000 pg/mL). For neat samples, the buffer was spiked instead of the calibrator. Spike recoveries were calculated according to the formula: % Recovery = [C spike sample-C neat sample /theoretical C spike] *100; C = concentration (pg/mL). Parallelism: Three different EV samples with high endogenous protein concentrations were serially diluted (2x, 4x and 8x). Both reciprocal relative dilution factor and OD450 absorbance signals of the samples and calibrator were log-transformed and linear regression was performed to calculate the slopes of the sample and calibrator curves. The slope of the linear parts of the log—log transformed calibrator and sample dilution series were compared to determine the degree of parallelism by calculating the "in range %" using the following formula: in range% = [slope of sample dilution /series slope of calibration curve] *100.

Other antibodies:
-Anti-Calnexin (cat no. C4731, Sigma-Aldrich, Darmstadt, Germany)
This antibody has been validated with an 'Enhanced Validity (EV)' certificate by the manufacturer. Manufacturer has tested this antibody on Western blot (WB)-Cell Line/Tissue Extract Hela or HepG2 Cells, WB Titer 1:2000-1:4000 and immuno-precipitation (IP) on Cell /Tissue Lysate Hela Cells. On 'CiteAb' antibody database (www.citeab.com), this antibody has been cited in 233 publications where around 55% of the assays were performed on human origin samples followed by mouse.
-Anti-Flotillin-2 (cat no. 610384, BD Biosciences, San Jose, CA, USA)
According to the manufacturer, this antibody has been QC tested on human samples. On 'CiteAb' antibody database (www.citeab.com), this antibody has been cited in 17 publications where almost 75% of the assays were performed on human origin samples followed by mouse. Reported application is Western Blot (WB) at 1:1000 dilution.
-Biotinylated mouse anti-human CD171 (L1CAM neural adhesion protein) antibody (clone 5G3
eBiosciences, San Diego, CA, USA (Cat no. 13171982, Thermo Fisher Scientific))
On 'CiteAb' antibody database (www.citeab.com), this antibody has been cited in 37 publications all assays were performed on human origin samples. Reported applications are Western Blot (WB), ELISA and FC/FACS. This antibody has been used to capture extracellular vesicle (EVs) from serum in Winston et al. 'Evaluation of blood-based, extracellular vesicles as biomarkers for aging-related TDP-43 pathology.' Alzheimers Dement (Amst). 2022 Dec 15;14(1):e12365. doi: 10.1002/dad2.12365. PMID: 36540894; PMCID: PMC9753157.
-HT7 (cat no. MN1000B, Thermo Fisher Scientific, epitope residues 159 – 163)
According to the manufacturer, this antibody has been QC tested on human samples. On 'CiteAb' antibody database (www.citeab.com), this antibody has been cited in over 100 publications where almost 60% of the assays were performed on human origin samples followed by mouse and rat. Reported application is Western Blot (WB), ELISA, IHC and ICC. This antibody is also used in Tau ELISA from Fujirebio which is widely used in the clinic for diagnosis of Alzheimer disease (AD) patients.
Pathology: Tau1 (provided by Drs. Nicholas Kanaan and Lester Binder) and HJ8.5 (provided by Dr. David Holtzman)
antibodies against phosphorylated TDP-43 (clone 1D3110), phosphorylated tau (clone AT8, cat no. MN1020,Thermo Fisher), α-synuclein (clone 4D6, cat no. AM05094PU-N, Origene), and beta-Amyloid (clone 4G8, cat no. SIG-39220, Covance).
These antibodies are widely used antibodies and regularly used in diagnosis confirmation post-mortem.

Eukaryotic cell lines

Policy information about cell lines and Sex and Gender in Research

Cell line source(s)	SH-SYSY cells
Authentication	SH-SY5Y cell line was purchased from ATCC (cat no. CRL-2266)
Mycoplasma contamination	cells were not tested for mycoplasma contamination
Commonly misidentified lines (See ICLAC register)	none

Clinical data

Policy information about clinical studies
All manuscripts should comply with the ICMJE guidelines for publication of clinical research and a completed CONSORT checklist must be included with all submissions.

Clinical trial registration	Cohort study, no clinical intervention trial.
Study protocol	available upon reasonable request
Data collection	Ongoing recruitment
Outcomes	No defined outcomes, since longitudinal cohort study

Plants

Seed stocks

Report on the source of all seed stocks or other plant material used. If applicable, state the seed stock centre and catalogue number. If plant specimens were collected from the field, describe the collection location, date and sampling procedures.

Novel plant genotypes

Describe the methods by which all novel plant genotypes were produced. This includes those generated by transgenic approaches, gene editing, chemical/radiation-based mutagenesis and hybridization. For transgenic lines, describe the transformation method, the number of independent lines analyzed and the generation upon which experiments were performed. For gene-edited lines, describe the editor used, the endogenous sequence targeted for editing, the targeting guide RNA sequence (if applicable) and how the editor was applied.

Authentication

Describe any authentication procedures for each seed stock used or novel genotype generated. Describe any experiments used to assess the effect of a mutation and, where applicable, how potential secondary effects (e.g. second site T-DNA insertions, mosiacism, off-target gene editing) were examined.
