# Supplementary material for: Plasma extracellular vesicle tau and TDP-43 as diagnostic biomarkers in FTD and ALS
Source: Nat Med. 2024 Jun 18;30(6):1771–83. doi: 10.1038/s41591-024-02937-4 (PMC11186765; doi:10.1038/s41591-024-02937-4)
Supplement: Supplementary file 3 — Supplementary Tables 1–22. [file 41591_2024_2937_MOESM3_ESM.pdf]

**Supplementary Table 1: 3R-4R Tau and TDP-43 assay vadalidation parameters in plasma sEV and mEV**

| 3R and 4R Tau Assay Validation | | | | | TDP-43 Assay Validation | |
|---|---|---|---|---|---|---|
| Parameters | sEV 3R Tau | mEV 3R Tau | sEV 4R Tau | mEV 4R Tau | sEV TDP-43 | mEV TDP-43 |
| LLOQ (conc) pg/mL | 102.85 | 119.08 | 122.54 | 132.89 | 8.56 | 7.78 |
| Avg % Linearity | 101.30 | 103.30 | 109.03 | 100.32 | 103.65 | 111.02 |
| Mean % Recovery | 101.24 | 95.69 | 99.84 | 96.25 | 97.95 | 104.40 |
| Avg % Parallelism | 110.18 | 112.71 | 106.55 | 104.08 | 99.38 | 109.64 |
| CV (%) for intra-assay precision | 6.59 | 6.54 | 3.38 | 3.21 | 4.78 | 5.32 |
| CV (%) for inter-assay precision | 7.95 | 7.63 | 4.54 | 4.39 | 5.21 | 6.60 |

**Supplementary Table 2: Two-sided Spearman correlation between EV 3R/4R Tau ratio, age, sex and disease duration in DESCRIBE subcohort 1**

| | Spearman *r* values | *p* values |
|---|---|---|
| Parameters | sEV 3R/4R Tau ratio | sEV 3R/4R Tau ratio |
| Age | 0.089 | 0.185 |
| Sex | 0.063 | 0.123 |
| Disease duration | 0.056 | 0.147 |

**Supplementary Table 3: The receiver operating characteristic area under the curve of diagnostic groups for sEV and mEV 3R/4R Tau ratio, sEV and mEV TDP-43 levels and plasma Nfl levels**

| **DESCRIBE subcohort 1** | AUC | SE [a] | 95% CI [b] | *p* value |
|---|---|---|---|---|
| **PSP vs. HC** | | | | |
| sEV 3R/4R Tau Ratio | 0.96 | 0.035 | 0.834 to 0.984 | 0.000023 |
| **PSP vs. AD** | | | | |
| sEV 3R/4R Tau Ratio | 0.99 | 0.004 | 0.90 to 1.000 | 0.000067 |
| **PSP vs. svPPA** | | | | |
| sEV 3R/4R Tau Ratio | 0.96 | 0.006 | 0.896 to 0.975 | 0.00009 |
| **PSP vs bvFTD** | | | | |
| sEV 3R/4R Tau Ratio | 0.99 | 0.007 | 0.937 to 1.000 | 0.00007 |
| **bvFTD vs. HC** | | | | |
| sEV 3R/4R Tau Ratio | 0.93 | 0.023 | 0.884 to 0.996 | 0.00007 |

| **DESCRIBE subcohort 2** | AUC | SE [a] | 95% CI [b] | *p* value |
|---|---|---|---|---|
| **PSP vs. HC** | | | | |
| sEV 3R/4R Tau Ratio | 0.98 | 0.007 | 0.960 to 0.997 | 0.000062 |
| mEV 3R/4R Tau Ratio | 0.90 | 0.022 | 0.855 to 0.938 | 0.000058 |
| sEV and mEV AUC comparison | | 0.045 | -0.012 to 0.036 | 0.0763 |
| **PSP vs ALS** | | | | |
| sEV 3R/4R Tau Ratio | 0.96 | 0.000 | 0.940 to 0.99 | 0.000064 |
| mEV 3R/4R Tau Ratio | 0.95 | 0.001 | 0.888 to 0.963 | 0.000021 |
| sEV and mEV AUC comparison | | 0.001 | -0.009 to 0.025 | 0.147 |
| **PSP vs. bvFTD** | | | | |
| sEV 3R/4R Tau Ratio | 0.98 | 0.006 | 0.973 to 0.997 | 0.00009 |
| mEV 3R/4R Tau Ratio | 0.97 | 0.007 | 0.958 to 0.991 | 0.00007 |
| sEV and mEV AUC comparison | | 0.044 | -0.011 to 0.023 | 0.354 |
| **bvFTD vs HC** | | | | |
| sEV 3R/4R Tau Ratio | 0.91 | 0.016 | 0.873 to 0.944 | 0.00001 |
| mEV 3R/4R Tau Ratio | 0.90 | 0.017 | 0.870 to 0.942 | 0.00004 |
| sEV and mEV AUC comparison | | 0.023 | -0.043 to 0.048 | 0.901 |
| **bvFTD&ALS** | | | | |
| sEV 3R/4R Tau Ratio | 0.89 | 0.004 | 0.874 to 0.913 | 0.00002 |
| mEV 3R/4R Tau Ratio | 0.86 | 0.003 | 0.084 to 0.902 | 0.00005 |
| sEV and mEV AUC comparison | | 0.024 | -0.087 to 0.541 | 0.149 |

| Subcohort 1 | AUC | SE [a] | 95% CI [b] | *p* value | Subcohort 2 | AUC | SE [a] | 95% CI [b] | *p* value |
|---|---|---|---|---|---|---|---|---|---|
| **bvFTD vs. AD** | | | | | | | | | |
| sEV 3R/4R Tau Ratio | 0.90 | 0.001 | 0.896 to 0.995 | 0.000033 | | | | | |
| **bvFTD vs. svPPA** | | | | | | | | | |
| sEV 3R/4R Tau Ratio | 0.95 | 0.000 | 0.901 to 0.975 | 0.000023 | | | | | |
| | | | | | **ALS vs HC** | | | | |
| | | | | | sEV TDP-43 (pg/mL) | 0.99 | 0.003 | 0.972 to 1.000 | 0.000022 |
| | | | | | mEV TDP-43 (pg/mL) | 0.92 | 0.020 | 0.866 to 0.952 | 0.00004 |
| | | | | | Plasma Nfl (pg/mL) | 0.80 | 0.030 | 0.766 to 0.880 | 0.0157 |
| | | | | | sEV and Plasma Nfl AUC comparison | | 0.032 | 0.107 to 0.228 | 0.000035 |
| | | | | | mEV and Plasma Nfl AUC comparison | | 0.041 | 0.126 to 0.232 | 0.000051 |
| | | | | | **ALS vs. PSP** | | | | |
| | | | | | sEV TDP-43 (pg/mL) | 0.99 | 0.003 | 0.980 to 1.000 | 0.000055 |
| | | | | | mEV TDP-43 (pg/mL) | 0.94 | 0.013 | 0.909 to 0.967 | 0.00002 |
| | | | | | Plasma Nfl (pg/mL) | 0.61 | 0.034 | 0.557 to 0.673 | 0.368 |
| | | | | | sEV and Plasma Nfl AUC comparison | | 0.034 | 0.312 to 0.447 | 0.00002 |
| | | | | | mEV and Plasma Nfl AUC comparison | | 0.036 | 0.254 to 0.398 | 0.00005 |
| | | | | | **ALS vs. bvFTD** | | | | |
| | | | | | sEV TDP-43 (pg/mL) | 0.91 | 0.016 | 0.878 to 0.943 | 0.000043 |
| | | | | | mEV TDP-43 (pg/mL) | 0.88 | 0.018 | 0.841 to 0.916 | 0.000085 |
| | | | | | Plasma Nfl (pg/mL) | 0.60 | 0.032 | 0.550 to 0.661 | 0.268 |
| | | | | | sEV and Plasma Nfl AUC comparison | | 0.036 | 0.237 to 0.378 | 0.00009 |

| **Subcohort 2** | AUC | SE [a] | 95% CI [b] | *p* value |
|---|---|---|---|---|
| **ALS vs. bvFTD** | | | | |
| mEV and Plasma Nfl AUC comparison | | 0.023 | 0.202 to 0.349 | 0.00004 |
| **bvFTD vs. HC** | | | | |
| sEV TDP-43 (pg/mL) | 0.85 | 0.0001 | 0.825 to 0.901 | 0.00047 |
| mEV TDP-43 (pg/mL) | 0.80 | 0.0015 | 0.783 to 0.853 | 0.00002 |
| Plasma Nfl (pg/mL) | 0.72 | 0.055 | 0.712 to 0.751 | 0.235 |
| sEV and Plasma Nfl AUC comparison | | 0.042 | 0.034 to 0.203 | 0.005 |
| mEV and Plasma Nfl AUC comparison | | 0.049 | 0.026 to 0.169 | 0.045 |
| **bvFTD vs. PSP** | | | | |
| sEV TDP-43 (pg/mL) | 0.93 | 0.000245 | 0.865 to 0.890 | 0.00007 |
| mEV TDP-43 (pg/mL) | 0.85 | 0.0353 | 0.783 to 0.848 | 0.00004 |
| Plasma Nfl (pg/mL) | 0.63 | 0.0055 | 0.612 to 0.713 | 0.235 |
| sEV and Plasma Nfl AUC comparison | | 0.041 | 0.202 to 0.169 | 0.00008 |
| mEV and Plasma Nfl AUC comparison | | 0.049 | 0.209 to 0.153 | 0.00004 |

[a] Hanley & McNeil. 1982[1]

[b] Binomial exact

SE: Standard Error

95% CI: 95% Confidence Interval

Paired t-test

1. Hanley, J. A. & McNeil, B. J. A method of comparing the areas under receiver operating characteristic curves derived from the same cases. *Radiology* **148**, 839-843 (1983). https://doi.org:10.1148/radiology.148.3.6878708

**Supplementary Table 4: Two-sided Spearman correlation between EV 3R/4R Tau ratio with clinical and neuropsychological and clinical scales in DESCRIBE subcohort 1: PSP cases**

| | Spearman *r* values | *p* values |
|---|---|---|
| Parameters | sEV 3R/4R Tau Ratio | sEV 3R/4R Tau Ratio |
| MMSE | **0.213** | **0.017** |
| MoCA | **0.468** | **0.0034** |
| NPI-Q | -0.334 | 0.053 |
| FAQ | -0.030 | 0.881 |
| PSP-RS | **-0.497** | **0.0007** |
| PSP-SS | **-0.512** | **0.0005** |
| PSP-CDS | **-0.402** | **0.0001** |
| UPDRS-III | **-0.551** | **0.0001** |
| SAS | **-0.345** | **0.002** |
| SEADL | **0.591** | **2.92067E-05** |
| CGIs | **-0.597** | **3.76451E-05** |
| FAB | **-0.355** | **0.02** |
| PSP-QoL | **-0.613** | **1.20461E-05** |
| Plasma Nfl | **-0.436** | **0.012** |

Bold values denote statistical significance at the $p < 0.05$ level.

**Supplementary Table 5: Two-sided Spearman correlation between EV 3R/4R Tau ratio with clinical and neuropsychological scales in DESCRIBE subcohort 1: bvFTD cases**

| | Spearman *r* values | *p* values |
|---|---|---|
| Parameters | sEV 3R/4R Tau Ratio | sEV 3R/4R Tau Ratio |
| MMSE | **-0.618** | **0.00001** |
| MoCA | **-0.667** | **0.00014** |
| NPI-Q | **0.445** | **0.0039** |
| FAQ | **0.512** | **0.00085** |
| CDR-SB | **0.523** | **0.00053** |
| CDR plus NACC FTLD | **0.503** | **0.00063** |
| CBI-M | **0.618** | **2.07836E-05** |
| Plasma Nfl | **0.512** | **0.00068** |

Bold values denote statistical significance at the $p < 0.05$ level.

**Supplementary Table 6: Two-sided Spearman correlation between CSF and plasma EV tau results in DESCRIBE subcohort 1**

| | Spearman *r* values | | | *p* values | | |
|---|---|---|---|---|---|---|
| | CSF sEV 3R Tau | CSF sEV 4R Tau | CSF sEV 3R/4R Tau ratio | CSF sEV 3R Tau | CSF sEV 4R Tau | CSF sEV 3R/4R Tau ratio |
| Plasma sEV 3R Tau | **0.240** | **-0.396** | **0.389** | **0.02** | **0.02** | **0.02** |
| Plasma sEV 4R Tau | -0.065 | 0.030 | 0.006 | 0.52 | 0.87 | 0.98 |
| Plasma sEV 3R/4R Tau ratio | 0.193 | -0.339 | 0.278 | 0.06 | 0.05 | 0.12 |

Bold values denote statistical significance at the $p < 0.05$ level.

Detected CSF EV samples: CSF sEV 3R Tau n=100. CSF sEV 4R Tau n=34.

**Supplementary Table 7: Two-sided Spearman correlation between EV 3R/4R Tau ratio, EV TDP-43, age, sex and disease duration in DESCRIBE subcohort 2**

| | Spearman *r* values | | | | *p* values | | | |
|---|---|---|---|---|---|---|---|---|
| Parameters | sEV TDP-43 | mEV TDP-43 | sEV 3R/4R Tau ratio | mEV 3R/4R Tau ratio | sEV TDP-43 | mEV TDP-43 | sEV 3R/4R Tau ratio | mEV 3R/4R Tau ratio |
| Age | 0.089 | 0.057 | 0.102 | 0.087 | 0.145 | 0.524 | 0.143 | 0.134 |
| Sex | 0.053 | -0.041 | 0.071 | 0.103 | 0.102 | 0.245 | 0.178 | 0.254 |
| Disease duration | 0.074 | 0.033 | -0.023 | -0.038 | 0.236 | 0.145 | 0.224 | 0.329 |

**Supplementary Table 8: Two-sided Spearman correlation between EV 3R/4R Tau ratio, EV TDP-43 and clinical and neuropsychological scales in DESCRIBE subcohort 2: PSP cases**

| | Spearman *r* values | | | | *p* values | | | |
|---|---|---|---|---|---|---|---|---|
| Parameters | sEV TDP-43 | mEV TDP-43 | sEV 3R/4R Tau ratio | mEV 3R/4R Tau ratio | sEV TDP-43 | mEV TDP-43 | sEV 3R/4R Tau ratio | mEV 3R/4R Tau ratio |
| MMSE | 0.062 | 0.0495 | **0.386** | **0.303** | 0.654 | 0.724 | **0.003** | **0.039** |
| MoCA | 0.056 | -0.070 | **0.363** | **0.306** | 0.538 | 0.445 | **0.002** | **0.039** |
| NPI-Q | 0.023 | 0.020 | **-0.367** | **-0.362** | 0.176 | 0.235 | **0.024** | **0.029** |
| FAQ | 0.013 | 0.018 | 0.0512 | 0.066 | 0.268 | 0.146 | 0.499 | 0.460 |
| PSP-RS | -0.007 | 0.030 | **-0.304** | **-0.377** | 0.930 | 0.730 | **0.002** | **0.003** |
| PSP-SS | 0.011 | -0.037 | **-0.383** | **-0.391** | 0.893 | 0.669 | **0.030** | **0.002** |
| PSP-CDS | 0.006 | -0.002 | **-0.365** | **-0.241** | 0.588 | 0.236 | **0.006** | **0.002** |
| UPDRS | 0.010 | -0.052 | **-0.225** | **-0.237** | 0.229 | 0.559 | **0.017** | **0.011** |
| SAS | -0.046 | -0.065 | **-0.387** | **-0.346** | 0.465 | 0.365 | **0.002** | **0.019** |
| SEADL | -0.022 | -0.042 | **0.390** | **0.396** | 0.791 | 0.623 | **0.002** | **0.002** |
| CGIs | -0.012 | 0.032 | **-0.226** | **-0.189** | 0.883 | 0.995 | **0.027** | **0.029** |
| FAB | 0.062 | -0.095 | **-0.194** | **-0.134** | 0.515 | 0.320 | **0.031** | **0.041** |
| PSP-QoL | -0.088 | -0.063 | **-0.306** | **-0.320** | 0.403 | 0.546 | **0.030** | **0.002** |
| Plasma Nfl | -0.006 | -0.011 | **-0.373** | **-0.352** | 0.941 | 0.178 | **0.013** | **0.025** |

Bold values denote statistical significance at the $p < 0.05$ level.

**Supplementary Table 9: Two-sided Spearman correlation between EV 3R/4R Tau ratio, EV TDP-43 and clinical and neuropsychological scales in DESCRIBE subcohort 2: bvFTD cases**

| | Spearman *r* values | | | | *p* values | | | |
|---|---|---|---|---|---|---|---|---|
| Parameters | sEV TDP-43 | mEV TDP-43 | sEV 3R/4R Tau ratio | mEV 3R/4R Tau ratio | sEV TDP-43 | mEV TDP-43 | sEV 3R/4R Tau ratio | mEV 3R/4R Tau ratio |
| MMSE | **-0.301** | **-0.259** | **-0.220** | **-0.242** | **0.031** | **0.0423** | **0.034** | **0.031** |
| MoCA | **-0.332** | **-0.322** | **-0.253** | **-0.234** | **0.026** | **0.0318** | **0.029** | **0.022** |
| NPI-Q | **0.292** | **0.103** | **0.561** | **0.363** | **0.005** | **0.006** | **0.003** | **0.002** |
| FAQ | **0.281** | **0.257** | **0.371** | **0.254** | **0.001** | **0.004** | **0.003** | **0.003** |
| CDR-SB | **0.274** | **0.209** | **0.330** | **0.379** | **0.021** | **0.029** | **0.003** | **0.002** |
| CDR plus NACC FTLD | **0.461** | **0.371** | **0.391** | **0.469** | **0.001** | **0.0002** | **0.001** | **0.001** |
| CBI-M | **0.343** | **0.284** | **0.349** | **0.314** | **0.001** | **0.007** | **0.001** | **0.002** |
| Plasma Nfl | **0.234** | **0.22** | **0.347** | **0.316** | **0.002** | **0.0004** | **0.005** | **0.0002** |

Bold values denote statistical significance at the $p < 0.05$ level.

**Supplementary Table 10: Descriptive data of mutations in bvFTD and ALS**

| **Mutations (n=37)** | **Clinical Diagnosis** | **Mutation types** |
|---|---|---|
| *C9orf72* | bvFTD (n=13), ALS (n=5) | hexanucleotide $(G4C2)_n$ repeat expansion |
| *GRN* | bvFTD (n=4) | Pathogenic/p.Val8vs<br>Pathogenic/p.Cys26<br>Pathogenic/p.Ser226fs<br>Pathogenic/p.Met142fs |
| *VCP* | bvFTD (n=4) | Pathogenic/p.Gly157Arg<br>Pathogenic/p.Arg95Cys<br>Pathogenic/p.Gly159Arg<br>Pathogenic/p.Gly157Arg |
| ***TBK1* | bvFTD (n=1)<br>ALS (n=1) | Potentially Pathogenic/p.Leu555Ter<br>Potentially Pathogenic/p.Glu643del |
| ***MAPT* | bvFTD (n=3) | Pathogenic/P301L<br>Pathogenic/P364S<br>Pathogenic/IVS 10+16 C>T |
| *CHCHD10* | bvFTD (n=1)<br>ALS (n=1) | Pathogenic/p.Ser59Leu<br>Pathogenic/p.Arg15Leu |
| ***SOD-1* | ALS (n=2) | Pathogenic/p.Gly115Ser<br>Pathogenic/p.Leu145Phe |
| *FUS* | ALS (n=2) | Pathogenic/p.Pro525Leu<br>Pathogenic/p.Ser57Del |

*2 *TBK1*, 1 *MAPT*, 1 *CHCHD10,* and 1 *SOD-1* mutation carriers with autopsy confirmed diagnosis

## Supplementary Table 11: Descriptive data of neuropathological diagnoses in ALS, bvFTD, and PSP

| Neuropathological diagnosis | ADNC | Lewy pathology stage | Other neuropathological findings | Gene mutation | Clinical diagnosis |
|---|---|---|---|---|---|
| ALS-TDP | A3, B2, C2 | | CAA | | ALS |
| ALS-TDP | A1, B2, C0 | | CAA | | ALS |
| ALS-TDP | A0, B2, C0 | | | | ALS |
| ALS-TDP | A0, B1, C0 | | | | ALS |
| ALS-TDP | A1, B1, C0 | | CAA | | ALS |
| ALS-TDP | A0, B1, C0 | | | | ALS |
| ALS-TDP | A1, B2, C0 | | ARTAG | | ALS |
| ALS-TDP | A1, B1, C0 | | | | ALS |
| ALS-TDP | A1, B1, C0 | | | | ALSci |
| ALS-TDP | A0, B2, C0 | | CAA | | ALSci |
| ALS-TDP | A1, B1, C0 | | | | ALS |
| ALS-TDP | A1, B1, C0 | brainstem predominant | | | ALS |
| ALS-TDP | A0, B0, C0 | | | | ALS |
| ALS-TDP | A0, B1, C0 | brainstem predominant | | | ALSci |
| ALS-TDP | A1, B1, C0 | | | | ALS |
| ALS-TDP | A0, B1, C0 | brainstem predominant | CAA | | ALS |
| ALS-TDP | A1, B2, C0 | | | *TBK1* | ALS |
| ALS/FTLD-TDP (subtype B) | A1, B1, C0 | | CAA | | ALSci |
| ALS/FTLD-TDP (subtype B) | A1, B1, C0 | | | | bvFTD+ALS |
| ALS/FTLD-TDP (subtype B) | A1, B2, C1 | | B cell lymphoma, CAA | | ALSci |
| ALS/FTLD-TDP (subtype B) | A0, B0, C0 | | granulomatous angiitis | | ALSci/ALSbi |
| ALS/FTLD-TDP (subtype B) | A0, B1, C0 | brainstem predominant | | | ALS+PPA |
| ALS/FTLD-TDP (subtype B) | A0, B1,C0 | | | | bvFTD |
| FTLD-TDP (subtype A) | A1, B1, C0 | | | *TBK1* | bvFTD |
| FTLD-Tau (GGT type) | A2, B1, C1 | | CAA | | ALSbi |
| FTLD-Tau (*MAPT* mutation*)* | A1, BX, C0 | | CAA | *MAPT P301L* | bvFTD |
| PSP | A0, B0, C0 | | | | PSP (possible) |
| PSP | A2, B1, C0 | | AGD, ATRAG | | PSP (probable) |
| PSP | A3, B2, C1 | | AGD | | PSP (possible) |
| ALS-SOD1 | A1, B1, C0 | | CAA | *SOD-1* | ALSci |
| ALS-UPS | A1, B1, C0 | none | | *CHCHD10* | ALS |

ADNC, Alzhe+mer d+sease neuropatholog+cal change; AGD, argyrophilic grain disease; ALS, amyotrophic lateral sclerosis; ALSci, amyotrophic lateral sclerosis, cognitively impaired; ALSbi, amyotrophic lateral sclerosis, behaviorally impaired; ALS-TDP, ALS with TDP-43 pathology; ALS-SOD1, ALS due to SOD1 mutation; ALS-UPS, ALS with TDP-43/tau/FUS negative inclusions positive for markers of ubiquitin-proteasome system; ARTAG, aging related tau astrogliopathy; CAA, cerebral amyloid angiopathy; CBD, corticobasal degeneration; FTLD-tau, frontotemporal lobar degeneration with tau pathology; FTLD-TDP, frontotemporal lobar degeneration with TDP-43 pathology; PPA, primary progressive aphasia; svPPA, semantic variant of PPA; bvFTD, behavioral variant of FTD. PSP, progressive supranuclear palsy;

**Supplementary Table 13: Two-sided Spearman correlation between EV 3R/4R Tau ratio, age, sex, and disease duration in Sant Pau cohort**

| | Spearman *r* values | | *p* values | |
|---|---|---|---|---|
| Parameters | sEV 3R/4R Tau ratio | sEV TDP-43 | sEV 3R/4R Tau ratio | sEV TDP-43 |
| Age | 0.005 | 0.087 | 0.256 | 0.274 |
| Sex | 0.075 | 0.096 | 0.345 | 0.330 |
| Disease duration | 0.023 | 0.156 | 0.145 | 0.089 |

**Supplementary Table 14: The receiver operating characteristic area under the curve of diagnostic groups for EV 3R/4R Tau ratio and EV TDP-43 levels**

| Sant Pau cohort | AUC | SE [a] | 95% CI [b] | *p* value |
|---|---|---|---|---|
| **PSP vs. HC** | | | | |
| sEV 3R/4R Tau Ratio | 1.00 | 0.000 | 0.960 to 1.000 | 0.00004 |
| mEV 3R/4R Tau Ratio | 1.00 | 0.000 | 0.960 to 1.000 | 0.00001 |
| sEV and mEV AUC comparison | | 0.035 | -0.020 to 0.154 | 0.123 |
| **PSP vs ALS** | | | | |
| sEV 3R/4R Tau Ratio | 0.99 | 0.0002 | 0.962 to 1.000 | 0.00007 |
| mEV 3R/4R Tau Ratio | 0.94 | 0.0023 | 0.881 to 0.980 | 0.000023 |
| sEV and mEV AUC comparison | | 0.036 | -0.023 to 0.036 | 0.254 |
| **PSP vs ALS-FTD** | | | | |
| sEV 3R/4R Tau Ratio | 0.98 | 0.0149 | 0.937 to 0.999 | 0.000052 |
| mEV 3R/4R Tau Ratio | 0.93 | 0.0253 | 0.870 to 0.976 | 0.000044 |
| sEV and mEV AUC comparison | | 0.0456 | -0.0032 to 0.076 | 0.231 |
| **PSP vs bvFTD** | | | | |
| sEV 3R/4R Tau Ratio | 1.00 | 0.000 | 0.969 to 1.000 | 0.000056 |
| mEV 3R/4R Tau Ratio | 1.00 | 0.000 | 0.969 to 1.000 | 0.000012 |
| sEV and mEV AUC comparison | | 0.085 | -0.051 to 0.110 | 0.114 |
| **bvFTD vs HC** | | | | |
| sEV 3R/4R Tau Ratio | 0.95 | 0.0163 | 0.905 to 0.985 | 0.000032 |
| mEV 3R/4R Tau Ratio | 0.96 | 0.0254 | 0.918 to 0.990 | 0.000014 |
| sEV and mEV AUC comparison | | 0.0751 | -0.041 to 0.230 | 0.420 |
| **bvFTD vs ALS** | | | | |
| sEV 3R/4R Tau Ratio | 0.90 | 0.0239 | 0.845 to 0.948 | 0.000047 |
| mEV 3R/4R Tau Ratio | 0.93 | 0.0135 | 0.875 to 0.966 | 0.00003 |
| sEV and mEV AUC comparison | | 0.0441 | -0.017 to 0.135 | 0.118 |
| **bvFTD vs ALS-FTD** | | | | |
| sEV 3R/4R Tau Ratio | 0.96 | 0.0140 | 0.920 to 0.990 | 0.00001 |
| mEV 3R/4R Tau Ratio | 0.97 | 0.0125 | 0.931 to 0.994 | 0.00004 |
| sEV and mEV AUC comparison | | 0.046 | -0.017 to 0.141 | 0.144 |
| **Sant Pau cohort** | AUC | SE [a] | 95% CI [b] | *p* value |
| **ALS vs HC** | | | | |
| sEV TDP-43 (pg/mL) | 0.94 | 0.0184 | 0.892 to 0.981 | 0.00007 |
| mEV TDP-43 (pg/mL) | 0.93 | 0.241 | 0.867 to 0.969 | 0.00001 |
| sEV and mEV AUC comparison | | 0.051 | -0.017 to 0.135 | 0.532 |
| **ALS vs. ALS-FTD** | | | | |
| sEV TDP-43 (pg/mL) | 0.58 | 0.0519 | 0.491 to 0.673 | 0.1051 |
| mEV TDP-43 (pg/mL) | 0.56 | 0.0523 | 0.476 to 0.658 | 0.186 |
| sEV and mEV AUC comparison | | 0.046 | -0.017 to 0.245 | 0.148 |
| **ALS vs. PSP** | | | | |

| | | | | |
|---|---|---|---|---|
| sEV TDP-43 (pg/mL) | 0.96 | 0.0147 | 0.910 to 0.991 | 0.00004 |
| mEV TDP-43 (pg/mL) | 0.96 | 0.0151 | 0.910 to 0.991 | 0.00007 |
| sEV and mEV AUC comparison | | 0.055 | -0.021 to 0.125 | 0.117 |
| **ALS vs. bvFTD** | | | | |
| sEV TDP-43 (pg/mL) | 0.76 | 0.0376 | 0.687 to 0.832 | 0.000045 |
| mEV TDP-43 (pg/mL) | 0.76 | 0.0393 | 0.691 to 0.835 | 0.000023 |
| sEV and mEV AUC comparison | | 0.052 | -0.021 to 0.120 | 0.432 |
| **bvFTD vs HC** | | | | |
| sEV TDP-43 (pg/mL) | 0.87 | 0.0311 | 0.803 to 0.926 | 0.00004 |
| mEV TDP-43 (pg/mL) | 0.82 | 0.0365 | 0.744 to 0.884 | 0.00007 |
| Plasma Nfl (pg/mL) | 0.78 | 0.0265 | 0.734 to 0.842 | 0.003 |
| sEV and mEV TDP-43 AUC comparison | | 0.062 | -0.045 to 0.121 | 0.632 |
| sEV and Plasma Nfl AUC comparison | | 0.0312 | 0.116 to 0.21 | 0.00008 |
| mEV and Plasma Nfl AUC comparison | | 0.016 | 0.112 to 0.156 | 0.00007 |
| **bvFTD vs. ALS-FTD** | | | | |
| sEV TDP-43 (pg/mL) | 0.82 | 0.0353 | 0.745 to 0.881 | 0.00001 |
| mEV TDP-43 (pg/mL) | 0.81 | 0.0363 | 0.736 to 0.874 | 0.00004 |
| sEV and mEV AUC comparison | | 0.0785 | -0.012 to 0.187 | 0.119 |
| **bvFTD vs. PSP** | | | | |
| sEV TDP-43 (pg/mL) | 0.91 | 0.0260 | 0.851 to 0.959 | 0.00007 |
| mEV TDP-43 (pg/mL) | 0.89 | 0.0273 | 0.830 to 0.947 | 0.00009 |
| Plasma Nfl (pg/mL) | 0.71 | 0.0365 | 0.689 to 0.789 | 0.0874 |
| sEV and mEV AUC comparison | | 0.063 | -0.046 to 0.176 | 0.896 |
| sEV and Plasma Nfl AUC comparison | | 0.0152 | 0.102 to 0.256 | 0.00007 |
| mEV and Plasma Nfl AUC comparison | | 0.0315 | 0.147 to 0.175 | 0.00001 |
| **ALS-FTD vs. HC** | | | | |
| sEV TDP-43 (pg/mL) | 0.98 | 0.0003 | 0.946 to 0.999 | 0.000045 |
| mEV TDP-43 (pg/mL) | 0.98 | 0.0025 | 0.933 to 0.997 | 0.000014 |
| sEV and mEV AUC comparison | | 0.075 | -0.012 to 0.176 | 0.119 |
| **ALS-FTD vs. PSP** | | | | |
| sEV TDP-43 (pg/mL) | 0.99 | 0.0004 | 0.955 to 1.000 | 0.00007 |
| mEV TDP-43 (pg/mL) | 0.99 | 0.0003 | 0.954 to 1.000 | 0.00008 |
| sEV and mEV AUC comparison | | 0.030 | -0.018 to 0.221 | 0.387 |

[a] Hanley & McNeil. 1982

[b] Binomial exact

SE: Standard Error

95% CI: 95% Confidence Interval

Paired t-test

**Supplementary Table 15: Descriptive data of mutations in bvFTD and ALS in Sant Pau cohort**

| **Mutations (n=34)** | **Clinical Diagnosis** | **Mutation types** |
|---|---|---|
| *C9orf72* | bvFTD (n=12), ALS (n=1), ALS-FTD (n=3) | hexanucleotide (G4C2)$_n$ repeat expansion |
| *GRN* | bvFTD (n=6) | Pathogenic/c.933+1G>A<br>Pathogenic/c.933+1G>A<br>Pathogenic/c.933+1G>A<br>Pathogenic/c.933+1G>A<br>Pathogenic/ c.1095-1096del<br>Pathogenic/ c.1095-1096del |
| *VCP* | bvFTD (n=1) | Pathogenic/ K618X |
| *TBK1* | bvFTD (n=3) | Pathogenic/ W445X<br>Pathogenic/ H528Y<br>Pathogenic/ Asp135Asn |
| *SOD-1* | ALS (n=3), ALS-FTD (n=1) | Pathogenic/ c.112G>A<br>Pathogenic/ c.339C>G<br>Pathogenic/ c.112G>A<br>Pathogenic/ c.339C>G |
| *FUS* | ALS (n=3) | Pathogenic/ c.1562G>A<br>Pathogenic/ c.169_171delTCT<br>Pathogenic/c.1561C>T |
| *TARDBP n=1* | bvFTD (n=1) | Pathogenic/A90V |

**Supplementary Table 16: Two-sided Spearman correlation between sEV 3R/4R Tau ratio, sEV TDP-43, and clinical and neuropsychological scales in Sant Pau cohort: PSP cases**

| | Spearman *r* values | | | | *p* values | | | |
|---|---|---|---|---|---|---|---|---|
| Parameters | sEV TDP-43 | sEV 3R/4R Tau ratio | mEV TDP-43 | mEV 3R/4R Tau ratio | sEV TDP-43 | sEV 3R/4R Tau ratio | mEV TDP-43 | mEV 3R/4R Tau ratio |
| MMSE | -0.097 | **0.183** | -0.085 | **0.164** | 0.590 | **0.018** | 0.658 | **0.039** |
| PSP-RS | 0.008 | **-0.202** | 0.052 | **-0.167** | 0.961 | **0.009** | 0.452 | **0.002** |
| UPDRS-III | -0.098 | **-0.708** | -0.074 | **-0.304** | 0.392 | **0.006** | 0.365 | **0.001** |
| FAB | -0.165 | **-0.630** | -0.023 | **-0.307** | 0.431 | **0.003** | 0.152 | **0.032** |
| Plasma Nfl | 0.102 | **-0.425** | 0.053 | **-0.402** | 0.563 | **0.0001** | 0.223 | **0.001** |
| Bold values denote statistical significance at the $p < 0.05$ level. | | | | | | | | |

**Supplementary Table 17: Two-sided Spearman correlation between sEV 3R/4R Tau ratio, sEV TDP-43, and clinical and neuropsychological scales in Sant Pau cohort: bvFTD cases**

| | Spearman *r* values | | | | *p* values | | | |
|---|---|---|---|---|---|---|---|---|
| Parameters | sEV TDP-43 | sEV 3R/4R Tau ratio | mEV TDP-43 | mEV 3R/4R Tau ratio | sEV TDP-43 | sEV 3R/4R Tau ratio | mEV TDP-43 | mEV 3R/4R Tau ratio |
| MMSE | **-0.200** | **-0.209** | **-0.184** | **-0.171** | **0.018** | **0.016** | **0.022** | **0.026** |
| NPI-Q | **0.209** | **0.239** | **0.209** | **0.205** | **0.009** | **0.008** | **0.019** | **0.008** |
| CDR-SB | **0.280** | **0.269** | **0.284** | **0.235** | **0.006** | **0.007** | **0.036** | **0.046** |
| CDR plus NACC FTLD | **0.267** | **0.277** | **0.259** | **0.254** | **0.003** | **0.011** | **0.037** | **0.039** |
| Plasma Nfl | **0.460** | **0.457** | **0.303** | **0.386** | **0.002** | **0.0001** | **0.008** | **0.001** |

Bold values denote statistical significance at the $p < 0.05$ level.

**Supplementary Table 18: Two-sided Spearman correlation between sEV 3R/4R Tau ratio, sEV TDP-43, and clinical and neuropsychological scales in Sant Pau cohort: ALS cases**

| | Spearman *r* values | | | | *p* values | | | |
|---|---|---|---|---|---|---|---|---|
| Parameters | sEV TDP-43 | mEV 3R/4R Tau ratio | mEV TDP-43 | mEV 3R/4R Tau ratio | sEV TDP-43 | sEV 3R/4R Tau ratio | mEV TDP-43 | mEV 3R/4R Tau ratio |
| ALS FRS-R | **-0.212** | -0.032 | **-0.200** | 0.056 | **0.015** | 0.776 | **0.011** | 0.236 |
| Time since diagnosis | **0.514** | 0.065 | **0.233** | 0.035 | **0.0004** | 0.967 | **0.029** | 0.102 |

Bold values denote statistical significance at the $p < 0.05$ level.

**Supplementary Table 19: Two-sided Spearman correlation between sEV 3R/4R Tau ratio, sEV TDP-43, and clinical and neuropsychological scales in Sant Pau cohort: ALS-FTD cases**

| | Spearman *r* values | | | | *p* values | | | |
|---|---|---|---|---|---|---|---|---|
| Parameters | sEV TDP-43 | sEV 3R/4R Tau ratio | mEV TDP-43 | mEV 3R/4R Tau ratio | sEV TDP-43 | sEV 3R/4R Tau ratio | mEV TDP-43 | mEV 3R/4R Tau ratio |
| MMSE | **-0.535** | 0.0319 | **-0.533** | 0.036 | **0.0182** | 0.971 | **0.018** | 0.452 |
| ECAS Total | **-0.799** | 0.0492 | **-0.778** | 0.052 | **0.0001** | 0.790 | **0.002** | 0.103 |
| ALS FRS-R | **-0.702** | 0.124 | **-0.699** | 0.123 | **0.0002** | 0.375 | **0.003** | 0.189 |
| Time since diagnosis | **0.445** | -0.090 | **0.404** | 0.009 | **0.0005** | 0.491 | **0.001** | 0.320 |

Bold values denote statistical significance at the $p < 0.05$ level.

**Supplementary Table 20: Comparisons of AUCs and CIs for the different cohorts**

| | AUC | SE [a] | 95% CI [b] | *p* value |
|---|---|---|---|---|
| **3R/4R Tau ratio** | | | | |
| **PSP vs. HC** | | | | |
| Subcohort 1 sEV 3R/4R Tau Ratio | 0.96 | 0.035 | 0.834 to 0.984 | 0.00004 |
| Subcohort 2 sEV 3R/4R Tau Ratio | 0.98 | 0.007 | 0.960 to 0.997 | 0.000078 |
| Sant Pau sEV 3R/4R Tau Ratio | 1.00 | 0.000 | 0.960 to 1.000 | 0.00002 |
| **PSP vs. bvFTD** | | | | |
| Subcohort 1 sEV 3R/4R Tau Ratio | 0.99 | 0.007 | 0.937 to 1.000 | 0.00001 |
| Subcohort 2 sEV 3R/4R Tau Ratio | 0.98 | 0.006 | 0.973 to 0.997 | 0.00003 |
| Sant Pau sEV 3R/4R Tau Ratio | 1.00 | 0.000 | 0.969 to 1.000 | 0.000045 |
| **bvFTD vs HC** | | | | |
| Subcohort 1 sEV 3R/4R Tau Ratio | 0.93 | 0.023 | 0.884 to 0.996 | 0.000041 |
| Subcohort 2 sEV 3R/4R Tau Ratio | 0.91 | 0.016 | 0.873 to 0.944 | 0.000052 |
| Sant Pau sEV 3R/4R Tau Ratio | 0.95 | 0.0163 | 0.905 to 0.985 | 0.00004 |
| **bvFTD&ALS** | | | | |
| Subcohort 1 n/a | | | | |
| Subcohort 2 sEV 3R/4R Tau Ratio | 0.89 | 0.004 | 0.874 to 0.913 | 0.00007 |
| Sant Pau sEV 3R/4R Tau Ratio | 0.90 | 0.0239 | 0.845 to 0.948 | 0.00006 |
| | AUC | SE [a] | 95% CI [b] | *p* value |
| **TDP-43** | | | | |
| **bvFTD vs. PSP** | | | | |
| Subcohort 1 n/a | | | | |
| Subcohort 2 sEV TDP-43 (pg/mL) | 0.93 | 0.0002 | 0.865 to 0.890 | 0.00008 |
| Sant Pau sEV TDP-43 (pg/mL) | 0.91 | 0.0260 | 0.851 to 0.959 | 0.000047 |
| **bvFTD vs HC** | | | | |
| Subcohort 1 n/a | | | | |
| Subcohort 2 sEV TDP-43 (pg/mL) | 0.85 | 0.0001 | 0.825 to 0.901 | 0.000045 |
| Sant Pau sEV TDP-43 (pg/mL) | 0.87 | 0.0311 | 0.803 to 0.926 | 0.00002 |
| **bvFTD vs. ALS** | | | | |
| Subcohort 1 n/a | | | | |
| Subcohort 2 sEV TDP-43 (pg/mL) | 0.91 | 0.016 | 0.878 to 0.943 | 0.00005 |
| Sant Pau sEV TDP-43 (pg/mL) | 0.76 | 0.0376 | 0.687 to 0.832 | 0.00007 |
| **ALS vs. HC** | | | | |
| Subcohort 1 n/a | | | | |
| Subcohort 2 sEV TDP-43 (pg/mL) | 0.99 | 0.003 | 0.972 to 1.000 | 0.00008 |

| | | | | | |
|---|---|---|---|---|---|
| Sant Pau | sEV TDP-43 (pg/mL) | 0.94 | 0.0184 | 0.892 to 0.981 | 0.00008 |
| **ALS vs. PSP** | | | | | |
| Subcohort 1 | n/a | | | | |
| Subcohort 2 | sEV TDP-43 (pg/mL) | 0.99 | 0.003 | 0.980 to 1.000 | 0.00004 |
| Sant Pau | sEV TDP-43 (pg/mL) | 0.96 | 0.0147 | 0.910 to 0.991 | 0.00006 |
| Paired t-test | | | | | |

**Supplementary Table 21: The receiver operating characteristic area under the curve(ROCAUC)/Precision recall curve (PRAUC) of diagnostic groups for sEV and mEV 3R/4R Tau ratio, sEV and mEV TDP-43 levels and plasma Nfl levels**

| DESCRIBE subcohort 1 | ROCAUC/ PRAUC | 95% CI | Disease Prevalence | DESCRIBE subcohort 2 | ROCAUC/ PRAUC | 95% CI | Disease Prevalence |
|---|---|---|---|---|---|---|---|
| **PSP vs. HC** | | | | **PSP vs. HC** | | | |
| sEV 3R/4R Tau Ratio | 0.96 | | | sEV 3R/4R Tau Ratio | 0.98 | | |
| sEV 3R/4R Tau Ratio PRAUC | 0.91 | 0.84 to 0.95 | 0.00005 for PSP | sEV 3R/4R Tau Ratio PRAUC | 0.90 | 0.83 to 0.96 | 0.00005 for PSP |
| | | | | mEV 3R/4R Tau Ratio | 0.90 | | |
| | | | | mEV 3R/4R Tau Ratio PRAUC | 0.85 | 0.81 to 0.92 | 0.00005 for PSP |
| **PSP vs. AD** | | | | **PSP vs ALS** | | | |
| sEV 3R/4R Tau Ratio | 0.99 | | | sEV 3R/4R Tau Ratio | 0.96 | | |
| sEV 3R/4R Tau Ratio PRAUC | 0.92 | 0.83 to 0.94 | 0.00005 for PSP | sEV 3R/4R Tau Ratio PRAUC | 0.89-0.90 | 0.86 to 0.93/0.85 to 0.91 | 0.00005 for PSP/0.000006 for ALS |
| | | | | mEV 3R/4R Tau Ratio | 0.91 | | |
| | | | | mEV 3R/4R Tau Ratio PRAUC | 0.86-0.87 | 0.83 to 0.91/0.84 to 0.92 | 0.00005 for PSP/0.000006 for ALS |
| **PSP vs. svPPA** | | | | **PSP vs. bvFTD** | | | |
| sEV 3R/4R Tau Ratio | 0.96 | | | sEV 3R/4R Tau Ratio | 0.98 | | |
| sEV 3R/4R Tau Ratio PRAUC | 0.90 | 0.86 to 0.92 | 0.00005 for PSP | sEV 3R/4R Tau Ratio PRAUC | 0.92-0.93 | 0.86 to 0.94/0.85 to 0.96 | 0.00005 for PSP/0.00015 for bvFTD |
| | | | | mEV 3R/4R Tau Ratio | 0.97 | | |
| | | | | mEV 3R/4R Tau Ratio PRAUC | 0.90-0.91 | 0.83 to 0.94/0.84 to 0.95 | 0.00005 for PSP/0.00015 for bvFTD |
| **PSP vs bvFTD** | | | | **bvFTD vs HC** | | | |
| sEV 3R/4R Tau Ratio | 0.99 | | | sEV 3R/4R Tau Ratio | 0.91 | | |

| | | | | | | | |
|---|---|---|---|---|---|---|---|
| sEV 3R/4R Tau Ratio PRAUC | 0.93-0.94 | 0.85 to 0.96/ 0.84 to 0.97 | 0.00005 for PSP / 0.00015 bvFTD | sEV 3R/4R Tau Ratio PRAUC | 0.86 | 0.81 to 0.90 | 0.00015 for bvFTD |
| | | | | mEV 3R/4R Tau Ratio | 0.90 | | |
| | | | | mEV 3R/4R Tau Ratio PRAUC | 0.84 | 0.79 to 0.89 | 0.00015 for bvFTD |
| **bvFTD vs. HC** | | | | **bvFTD vs. ALS** | | | |
| sEV 3R/4R Tau Ratio | 0.93 | | | sEV 3R/4R Tau Ratio | 0.89 | | |
| sEV 3R/4R Tau Ratio PRAUC | 0.86 | 0.81 to 0.91 | 0.00015 for bvFTD | sEV 3R/4R Tau Ratio PR | 0.83-0.85 | 0.78 to 0.92/0.80 to 0.93 | 0.00015 for bvFTD/0.000006 for ALS |
| | | | | mEV 3R/4R Tau Ratio | 0.86 | | |
| | | | | mEV 3R/4R Tau Ratio PRAUC | 0.80-0.82 | 0.76 to 0.89/0.78 to 0.92 | 0.00015 for bvFTD/0.000006 for ALS |
| **bvFTD vs. AD** | | | | | | | |
| sEV 3R/4R Tau Ratio | 0.90 | | | | | | |
| sEV 3R/4R Tau Ratio PRAUC | 0.88 | 0.86 to 0.93 | 0.00015 for bvFTD | | | | |
| | | | | | | | |
| | | | | | | | |
| **bvFTD vs. svPPA** | | | | | | | |
| sEV 3R/4R Tau Ratio | 0.95 | | | | | | |
| sEV 3R/4R Tau Ratio PRAUC | 0.91 | 0.86 to 0.93 | 0.00015 for bvFTD | | | | |
| | | | | **ALS vs HC** | | | |
| | | | | sEV TDP-43 (pg/mL) | 0.99 | | |

| | | | | |
|---|---|---|---|---|
| | sEV TDP-43 (pg/mL) PRAUC | 0.91 | 0.86 to 0.93 | 0.000006 for ALS |
| | mEV TDP-43 (pg/mL) | 0.92 | | |
| | mEV TDP-43 (pg/mL) PRAUC | 0.84 | 0.79 to 0.91 | 0.000006 for ALS |
| | Plasma Nfl (pg/mL) | 0.80 | | |
| | Plasma Nfl (pg/mL)PRAUC | 0,74 | 0.68 to 0.81 | 0.000006 for ALS |
| | **ALS vs. PSP** | | | |
| | sEV TDP-43 (pg/mL) | 0.99 | | |
| | sEV TDP-43 (pg/mL) PRAUC | 0.93-0.92 | 0.90 to 0.98/0.86 to 0.96 | 0.000006 for ALS/0.00005 for PSP |
| | mEV TDP-43 (pg/mL) | 0.94 | | |
| | mEV TDP-43 (pg/mL) PRAUC | 0.89-0.90 | 0.86 to 0.91/0.84 to 0.93 | 0.000006 for ALS/0.00005 for PSP |
| | Plasma Nfl (pg/mL) | 0.61 | | |
| | Plasma Nfl (pg/mL) PRAUC | 0.57-0.52 | 0.52 to 0.71/0.48 to 0.60 | 0.000006 for ALS/0.00005 for PSP |
| | **ALS vs. bvFTD** | | | |
| | sEV TDP-43 (pg/mL) | 0.91 | | |
| | sEV TDP-43 (pg/mL) PRAUC | 0.86-0.88 | 0.81 to 0.92/0.82 to 0.95 | 0.000006 for ALS/0.00015 for bvFTD |
| | mEV TDP-43 (pg/mL) | 0.88 | | |
| | mEV TDP-43 (pg/mL PRAUC | 0.82-0.85 | 0.78 to 0.89/0.83 to 0.96 | 0.000006 for ALS/0.00015 for bvFTD |
| | Plasma Nfl (pg/mL) | 0.60 | | |
| | Plasma Nfl (pg/mL) PRAUC | 0.55-0.52 | 0.80 to 0.68/0.50 to 0.63 | 0.000006 for ALS/0.00015 for bvFTD |

| | | | | |
|---|---|---|---|---|
| | **bvFTD vs. HC** | | | |
| | sEV TDP-43 (pg/mL) | 0.85 | | |
| | sEV TDP-43 (pg/mL) PRAUC | 0.81 | 0.78 to 0.86 | 0.00015 for bvFTD |
| | mEV TDP-43 (pg/mL) | 0.80 | | |
| | mEV TDP-43 (pg/mL) PRAUC | 0.76 | 0.72 to 0.82 | 0.00015 for bvFTD |
| | Plasma Nfl (pg/mL) | 0.72 | | |
| | Plasma Nfl (pg/mL) PRAUC | 0.63 | 0.55 to 0.71 | 0.00015 for bvFTD |
| | **bvFTD vs. PSP** | | | |
| | sEV TDP-43 (pg/mL) | 0.93 | | |
| | sEV TDP-43 (pg/mL) PRAUC | 0.89-0.91 | 0.86 to 0.92/0.89 to 0.96 | 0.00015 for bvFTD/0.00005 for PSP |
| | mEV TDP-43 (pg/mL) | 0.85 | | |
| | mEV TDP-43 (pg/mL) PRAUC | 0.80-0.82 | 0.76 to 0.89/0.77 to 0.89 | 0.00015 for bvFTD/0.00005 for PSP |
| | Plasma Nfl (pg/mL) | 0.63 | | |
| | Plasma Nfl (pg/mL) PRAUC | 0.52-0.50 | 0.48 to 0.60/0.45 to 0.63 | 0.00015 for bvFTD/0.00005 for PSP |

ALS prevalence 0.6 - 3.8 per 100 000 persons[1]
PSP prevalence 5–7 per 100,000 persons[2]
bvFTD prevalence 15-22 per 100 000 person[3]


1. Longinetti, E. & Fang, F. Epidemiology of amyotrophic lateral sclerosis: an update of recent literature. *Curr Opin Neurol* **32**, 771-776 (2019). https://doi.org:10.1097/WCO.0000000000000730
2. Barer, Y. *et al.* Epidemiology of Progressive Supranuclear Palsy: Real World Data from the Second Largest Health Plan in Israel. *Brain Sci* **12** (2022). https://doi.org:10.3390/brainsci12091126
3. Onyike, C. U. & Diehl-Schmid, J. The epidemiology of frontotemporal dementia. *Int Rev Psychiatry* **25**, 130-137 (2013). https://doi.org:10.3109/09540261.2013.776523

**Supplementary Table 22: The receiver operating characteristic area under the curve (ROCAUC)/Precision recall curve (PRAUC)of diagnostic groups for EV 3R/4R Tau ratio and EV TDP-43 levels, Sant Pau cohort**

| **Sant Pau cohort** | ROCAUC/ PRAUC | 95% CI | Disease Prevalence |
|---|---|---|---|
| **PSP vs. HC** | | | |
| sEV 3R/4R Tau Ratio | 1.00 | | |
| sEV 3R/4R Tau Ratio PRAUC | 0.92 | 0.86 to 0.93 | 0.00005 for PSP |
| mEV 3R/4R Tau Ratio | 1.00 | | |
| mEV 3R/4R Tau Ratio PRAUC | 0.89 | 0.83 to 0.91 | 0.00005 for PSP |
| **PSP vs ALS** | | | |
| sEV 3R/4R Tau Ratio | 0.99 | | |
| sEV 3R/4R Tau Ratio PRAUC | 0.92-0.91 | 0.89 to 0.96<br>0.88 to 0.93 | 0.00005 for PSP<br>/0.000006 for ALS |
| mEV 3R/4R Tau Ratio | 0.94 | | |
| mEV 3R/4R Tau Ratio PRAUC | 0.83-0.86 | 0.79 to 0.86<br>0.78 to 0.89 | 0.00005 for PSP<br>/0.000006 for ALS |
| **PSP vs ALS-FTD** | | | |
| sEV 3R/4R Tau Ratio | 0.98 | | |
| sEV 3R/4R Tau Ratio PRAUC | 0.90 | 0.83 to 0.96 | 0.00005 for PSP |
| mEV 3R/4R Tau Ratio | 0.93 | | |
| mEV 3R/4R Tau Ratio PRAUC | 0.82 | 0.76 to 0.89 | 0.00005 for PSP |
| **PSP vs bvFTD** | | | |
| sEV 3R/4R Tau Ratio | 1.00 | | |
| sEV 3R/4R Tau Ratio PRAUC | 0.93-0.92 | 0.89 to 0.98<br>0.88 to 0.96 | 0.00005for PSP<br>/0.00015 for bvFTD |
| mEV 3R/4R Tau Ratio | 1.00 | | |
| mEV 3R/4R Tau Ratio PRAUC | 0.90-0.91 | 0.86 to 0.94<br>0.88 to 0.96 | 0.00005for PSP<br>/0.00015 for bvFTD |
| **bvFTD vs HC** | | | |
| sEV 3R/4R Tau Ratio | 0.95 | | |
| sEV 3R/4R Tau Ratio PRAUC | 0.90 | 0.87 to 0.96 | 0.00015 for bvFTD |
| mEV 3R/4R Tau Ratio | 0.96 | | |
| mEV 3R/4R Tau Ratio PRAUC | 0.88 | 0.81 to 0.90 | 0.00015 for bvFTD |
| **bvFTD vs ALS** | | | |
| sEV 3R/4R Tau Ratio | 0.90 | | |
| sEV 3R/4R Tau Ratio PRAUC | 0.86-0.87 | 0.81 to 0.90<br>0.83 to 0.92 | 0.00015 for bvFTD<br>/0.000006 for ALS |
| mEV 3R/4R Tau Ratio | 0.93 | | |
| mEV 3R/4R Tau Ratio PRAUC | 0.83-0.84 | 0.78 to 0.91<br>0.80 to 0.93 | 0.00015 for bvFTD<br>/0.000006 for ALS |
| **bvFTD vs ALS-FTD** | | | |
| sEV 3R/4R Tau Ratio | 0.96 | | |

| | | | |
|---|---|---|---|
| sEV 3R/4R Tau Ratio PRAUC | 0.91 | 0.90 to 0.98 | 0.00015 for bvFTD |
| mEV 3R/4R Tau Ratio | 0.97 | | |
| mEV 3R/4R Tau Ratio PRAUC | 0.90 | 0.86 to 0.95 | 0.00015 for bvFTD |
| **Sant Pau cohort** | ROCAUC/ PRAUC | 95% CI | Disease Prevalence |
| **ALS vs HC** | | | |
| sEV TDP-43 (pg/mL) | 0.94 | | |
| sEV TDP-43 (pg/mL) PRAUC | 0.89 | 0.86 to 091 | 0.000006 for ALS |
| mEV TDP-43 (pg/mL) | 0.93 | | |
| mEV 3R/4R TDP-43 (pg/mL) PRAUC | 0.86 | 0.83 to 0.92 | 0.000006 for ALS |
| **ALS vs. ALS-FTD** | | | |
| sEV TDP-43 (pg/mL) | 0.58 | | |
| sEV TDP-43 (pg/mL) PRAUC | 0.45 | 0.38 to 0.52 | 0.000006 for ALS |
| mEV TDP-43 (pg/mL) | 0.56 | | |
| mEV TDP-43 (pg/mL) PRAUC | 0.49 | 0.41 to 0.53 | 0.000006 for ALS |
| **ALS vs. PSP** | | | |
| sEV TDP-43 (pg/mL) | 0.96 | | |
| sEV TDP-43 (pg/mL) PRAUC | 0.91-0.92 | 0.85 to 0.96<br>0.86 to 0.95 | 0.000006 for ALS/0.00005 for PSP |
| mEV TDP-43 (pg/mL) | 0.96 | | |
| mEV TDP-43 (pg/mL) PRAUC | 0.90-0.92 | 0.86 to 0.93<br>0.89 to 0.95 | 0.000006 for ALS/0.00005 for PSP |
| sEV and mEV AUC comparison | | | |
| **ALS vs. bvFTD** | | | |
| sEV TDP-43 (pg/mL) | 0.76 | | |
| sEV TDP-43 (pg/mL) PRAUC | 0.74-0.75 | 0.71 to 0.82<br>0.70 to 0.83 | 0.000006 for ALS/0.00015 for bvFTD |
| mEV TDP-43 (pg/mL) | 0.76 | | |
| mEV TDP-43 (pg/mL) PRAUC | 0.73-0.74 | 0.68 to 0.80<br>0.70 to 0.82 | 0.000006 for ALS/0.00015 for bvFTD |
| **bvFTD vs HC** | | | |
| sEV TDP-43 (pg/mL) | 0.87 | | |
| sEV (pg/mL) PRCAUC | 0.83 | 0.78 to 0.89 | 0.00015 for bvFTD |
| mEV TDP-43 (pg/mL) | 0.82 | | |
| mEV TDP-43 (pg/mL) PRAUC | 0.78 | 0.76 to 0.85 | 0.00015 for bvFTD |
| Plasma Nfl (pg/mL) | 0.78 | | |
| Plasma Nfl (pg/mL) PRAUC | 0.65 | 0.60 to 0.75 | |
| **bvFTD vs. ALS-FTD** | | | |
| sEV TDP-43 (pg/mL) | 0.82 | | |

| | | | |
|---|---|---|---|
| sEV TDP-43 (pg/mL) PRAUC | 0.78 | 0.75 to 0.85 | 0.00015 for bvFTD |
| mEV TDP-43 (pg/mL) | 0.81 | | |
| mEV TDP-43 (pg/mL) PRAUC | 0.76 | 0.72 to 0.84 | 0.00015 for bvFTD |
| **bvFTD vs. PSP** | | | |
| sEV TDP-43 (pg/mL) | 0.91 | | |
| sEV TDP-43 (pg/mL) PRAUC | 0.88-0.90 | 0.84 to 0.92<br>0.88 to 0.96 | 0.00015 for bvFTD/0.00005 for PSP |
| mEV TDP-43 (pg/mL) | 0.89 | | |
| mEV 3R/4R TDP-43 (pg/mL) PRAUC | 0.87 -088 | 0.85 to 0.93<br>0.84 to 0.95 | 0.00015 for bvFTD/0.00005 for PSP |
| Plasma Nfl (pg/mL) | 0.71 | | |
| Plasma Nfl (pg/mL) PRAUC | 0.60-0.61 | 0.58 to 0.70<br>0.55 to 0.72 | 0.00015 for bvFTD/0.00005 for PSP |
| **ALS-FTD vs. HC** | | | |
| sEV TDP-43 (pg/mL) | 0.98 | | |
| sEV TDP-43 (pg/mL) PRAUC | 0.94 | 0.90 to 0.98 | 0.000006 for ALS |
| mEV TDP-43 (pg/mL) | 0.98 | | |
| mEV 3R/4R TDP-43 (pg/mL) PRAUC | 0.93 | 0.90 to 0.97 | 0.000006 for ALS |
| | | | |
| **ALS-FTD vs. PSP** | | | |
| sEV TDP-43 (pg/mL) | 0.99 | | |
| sEV TDP-43 (pg/mL) PRAUC | 0.95-0.94 | 0.91 to 0.99<br>0.89 to 0.96 | 0.000006 for ALS/0.00005 for PSP |
| mEV TDP-43 (pg/mL) | 0.99 | | |
| mEV 3R/4R TDP-43 (pg/mL) PRAUC | 0.94-0.93 | 0.89 to 0.98<br>0.88 to 0.96 | 0.000006 for ALS/0.00005 for PSP |
| ALS prevalence 0.6 - 3.8 per 100 000 persons[1]<br>PSP prevalence 5–7 per 100,000 persons[2]<br>bvFTD prevalence 15-22 per 100 000 person[3] | | | |


1. Longinetti, E. & Fang, F. Epidemiology of amyotrophic lateral sclerosis: an update of recent literature. *Curr Opin Neurol* **32**, 771-776 (2019). https://doi.org:10.1097/WCO.0000000000000730
2. Barer, Y. *et al.* Epidemiology of Progressive Supranuclear Palsy: Real World Data from the Second Largest Health Plan in Israel. *Brain Sci* **12** (2022). https://doi.org:10.3390/brainsci12091126
3. Onyike, C. U. & Diehl-Schmid, J. The epidemiology of frontotemporal dementia. *Int Rev Psychiatry* **25**, 130-137 (2013). https://doi.org:10.3109/09540261.2013.776523
