# Supplementary material for: Plasma extracellular vesicle tau and TDP-43 as diagnostic biomarkers in FTD and ALS
Source: Nat Med. 2024 Jun 18;30(6):1771–83. doi: 10.1038/s41591-024-02937-4 (PMC11186765; doi:10.1038/s41591-024-02937-4)
Supplement: Supplementary file 4 — Exact P values for comparisons of Extended Data Tables 1–3. [file 41591_2024_2937_MOESM4_ESM.docx]

| **Characteristic** |  |  |  |  |  |  |  |  |  |  |
| --- | --- | --- | --- | --- | --- | --- | --- | --- | --- | --- |
| Groups |  |  |  |  |  |  |  |  |  |  |
| n (total 141) |  |  |  |  |  |  |  |  |  |  |
| **p values in DESCRIBE cohort: subcohort 1** | | | | | | | | | | |
|  | HC vs. AD | HC vs. svPPA | HC vs. bvFTD | HC vs. PSP | AD vs. svPPA | AD vs. bvFTD | AD vs. PSP | svPPA vs. bvFTD | svPPA vs. PSP | bvFTD vs. PSP |
| Age (mean ± SD) | *p* = 0.023 | - | - | - | - | - | - | - | - | - |
| Disease duration (median [IQR]) | - | - | - | - | - | - | *p* = 0.026 | - | - | - |
| Aβ1-42 (pg/ml)(median[IQR]) | *p* = 0.0016 | *p* = 0.002 | *p* = 0.031 | *p* = 0.0051 | *p* = 0.0029 | *p* = 0.0002 | *p* = 0.007 | - | - | - |
| Aβ42/40 (mean ± SD) | *p* = 0.027 | - | - | - | - | - | - | - | - | - |
| tTau (pg/ml)(median[IQR]) | *p* = 0.007 | *p* = 0.04 | *p* = 0.021 | *p* = 0.0014 | *p* = 0.009 | *p* = 0.0014 | *p* = 0.01 | - | - | - |
| pTau (pg/ml)(median[IQR]) | *p* = 0.0056 | *p* = 0.001 | *p* = 0.0049 | *p* = 0.0074 | *p* = 0.004 | *p* = 0.0068 | *p* = 0.004 | - | - | - |
| MMSE (mean ± SD) | *p* = 0.007 | - | *p* = 0.0085 | - | *p* = 0.02 | *p* = 0.019 | *p* = 0.0053 | - | - | - |
| MoCA (mean ± SD) | *p* = 0.005 |  | *p* = 0.001 | *p* = 0.03 | *p* = 0.004 | *p* = 0.0086 | - | - | - | - |
| NPI-Q (mean ± SD) | *p* = 0.02 | *p* = 0.0083 | *p* = 0.0057 | *p* = 0.004 | - | *p* = 0.0075 | - | - | - | - |
| FAQ (mean ± SD) | *p* = 0.01 | *p* = 0.024 | *p* = 0.001 | *p* = 0.0035 | - | - | - | - | - | - |
| CDR-SB (mean ± SD) | *p* = 0.046 | *p* = 0.007 | *p* = 0.0042 | - | *p* = 0.001 | *p* = 0.0068 | *p* = 0.0043 | - | - | - |
| CDR plus NACC FTLD (mean ± SD) | - | - | *p* = 0.0027 | - | - | - | - | - | - | - |
| CBI-M (mean ± SD) | - | - | *p* = 0.0013 | - | - | - | - | - | - | - |
| PSP-RS (mean ± SD) |  |  |  |  |  |  |  |  |  |  |
| PSP-SS (mean ± SD) |  |  |  |  |  |  |  |  |  |  |
| PSP-CDS (mean ± SD) |  |  |  |  |  |  |  |  |  |  |
| UPDRS III (mean ± SD) |  |  |  |  |  |  |  |  |  |  |
| SAS (mean ± SD) |  |  |  |  |  |  |  |  |  |  |
| SEADL (mean ± SD) |  |  |  |  |  |  |  |  |  |  |
| CGIs (mean ± SD) |  |  |  |  |  |  |  |  |  |  |
| FAB (mean ± SD) |  |  |  |  |  |  |  |  |  |  |
| PSP-QoL (mean ± SD) |  |  |  |  |  |  |  |  |  |  |
| Language (mean ± SD) |  |  |  |  |  |  |  |  |  |  |
| Verbal fluency (mean ± SD) |  |  |  |  |  |  |  |  |  |  |
| Executive (mean ± SD) |  |  |  |  |  |  |  |  |  |  |
| Memory (mean ± SD) |  |  |  |  |  |  |  |  |  |  |
| Visuospatial (mean ± SD) |  |  |  |  |  |  |  |  |  |  |
| ALS specific(mean ± SD) |  |  |  |  |  |  |  |  |  |  |
| ALS-nonspecific (mean ± SD) |  |  |  |  |  |  |  |  |  |  |
| ECAS Total (mean ± SD) |  |  |  |  |  |  |  |  |  |  |
| ALS FRS-R (mean ± SD) |  |  |  |  |  |  |  |  |  |  |
| Plasma Nfl (pg/mL)(median[IQR]) | *p* = 0.016 | *p* = 0.002 | *p* = 0.007 | *p* = 0.0089 | - | - | - | - | - | - |
| sEV 3R Tau (pg/mL)(median[IQR]) | - | - | *p* = 0.0002 | *p* = 0.0053 | *p* = 0.0025 | - | - | - | - | *p* = 0.000026 |
| sEV 4R Tau (pg/mL)(median[IQR]) | - | - | *p* = 0.0045 | *p* = 0.0013 | *p* = 0.007 | - | - | - | - | *p* = 0.000051 |
| sEV 3R/4R Tau ratio(median[IQR]) | - | - | *p* = 0.0003 | *p* = 0.000004 | - | *p* = 0.0003 | *p* = 0.0000052 | *p* = 0.0007 | *p* = 0.0000057 | *p* = 0.0000019 |

| **Characteristic** |  |  |  |  |  |  |
| --- | --- | --- | --- | --- | --- | --- |
| **Groups** |  |  |  |  |  |  |
| **n (total 704)** |  |  |  |  |  |  |
| **p values in DESCRIBE cohort: subcohort 2** | | | | | | |
|  | HC vs. ALS | HC vs. bvFTD | HC vs. PSP | ALS vs. bvFTD | ALS vs. PSP | bvFTD vs. PSP |
| Age (mean ± SD) | *p* = 0.039 | - | - | - | - | - |
| Disease duration (median [IQR]) | - | - | - | *p* = 0.024 | - | - |
| Aβ1-42 (pg/ml)(median[IQR]) | *p* = 0.016 | *p* = 0.034 | *p* = 0.021 | *-* | *-* | *-* |
| Aβ42/40 (mean ± SD) | *-* | - | - | - | - | - |
| tTau (pg/ml)(median[IQR]) | *p* = 0.013 | *p* = 0.026 | *p* = 0.031 | *-* | *-* | *-* |
| pTau (pg/ml)(median[IQR]) | *p* = 0.02 | *p* = 0.004 | *p* = 0.029 | *-* | *-* | *-* |
| MMSE (mean ± SD) | *p* = 0.029 | *p* = 0.012 | *p* = 0.024 | *-* | *-* | *-* |
| MoCA (mean ± SD) | *p* = 0.016 | *p* = 0.021 | *-* | *-* | *-* | *-* |
| NPI-Q (mean ± SD) | *p* = 0.018 | *p* = 0.032 | *p* = 0.026 | *-* | *-* | *-* |
| FAQ (mean ± SD) | *p* = 0.025 | *p* = 0.002 | *p* = 0.011 | *-* | *-* | *-* |
| CDR-SB (mean ± SD) | *p* = 0.005 | *p* = 0.034 | *p* = 0.007 | *-* | *-* | *-* |
| CDR plus NACC FTLD (mean ± SD) | - | *p* = 0.001 | *-* | - | - | - |
| CBI-M (mean ± SD) | - | *p* = 0.029 | *-* | - | - | - |
| PSP-RS (mean ± SD) |  |  |  |  |  |  |
| PSP-SS (mean ± SD) |  |  |  |  |  |  |
| PSP-CDS (mean ± SD) |  |  |  |  |  |  |
| UPDRS III (mean ± SD) |  |  |  |  |  |  |
| SAS (mean ± SD) |  |  |  |  |  |  |
| SEADL (mean ± SD) |  |  |  |  |  |  |
| CGIs (mean ± SD) |  |  |  |  |  |  |
| FAB (mean ± SD) |  |  |  |  |  |  |
| PSP-QoL (mean ± SD) |  |  |  |  |  |  |
| Language (mean ± SD) |  |  |  |  |  |  |
| Verbal fluency (mean ± SD) |  |  |  |  |  |  |
| Executive (mean ± SD) |  |  |  |  |  |  |
| Memory (mean ± SD) |  |  |  |  |  |  |
| Visuospatial (mean ± SD) |  |  |  |  |  |  |
| ALS specific(mean ± SD) |  |  |  |  |  |  |
| ALS-nonspecific (mean ± SD) |  |  |  |  |  |  |
| ECAS Total (mean ± SD) |  |  |  |  |  |  |
| ALS FRS-R (mean ± SD) |  |  |  |  |  |  |
| Plasma Nfl (pg/mL)(median[IQR]) | *p* = 0.021 | *p* = 0.003 | *p* = 0.014 | *-* |  |  |
| sEV 3R Tau (pg/mL)(median[IQR]) | - | *p* = 0.0002 | *p* = 0.0001 | *p* = 0.0019 | *p* = 0.001 | *p* = 0.0000023 |
| sEV 4R Tau (pg/mL)(median[IQR]) | - | *p* = 0.00014 | *p* = 0.00042 | *p* = 0.0087 | *p* = 0.0041 | *p* = 0.0000017 |
| mEV 3R Tau (pg/mL)(median[IQR]) | - | *p* = 0.003 | *p* = 0.002 | *p* = 0.0029 | *p* = 0.0047 | *p* = 0.0000015 |
| mEV 4R Tau (pg/mL)(median[IQR]) | - | *p* = 0.0001 | *p* = 0.0023 | *p* = 0.0058 | *p* = 0.007 | *p* = 0.0000011 |
| sEV 3R/4R Tau ratio(median[IQR]) | - | *p* = 0.0000057 | *p* = 0.000009 | *p* = 0.0000074 | *p = 0.0000023* | *p* = 0.0000067 |
| mEV 3R/4R Tau ratio(median[IQR]) | - | *p* = 0.0000052 | *p* = 0.0000012 | *p* = 0.0000097 | *p* = 0.0000056 | *p* = 0.0000041 |
| Plasma TDP-43 (pg/mL)(median[IQR]) | - | - | - | - | - | - |
| sEV TDP-43 (pg/mL)(median[IQR]) | *p* = 0.000003 | *p* = 0.000006 | - | *p* = 0.0000074 | *p* = 0.0000028 | *p* = 0.0000012 |
| mEV TDP-43 (pg/mL)(median[IQR]) | *p* = 0.0000028 | *p* = 0.0000054 | - | *p* = 0.0000043 | *p* = 0.0000012 | *p* = 0.0000093 |
| EV particles (x10^7^/mL) | - | - | - | - | - | - |

| **Characteristic** |  |  |  |  |  |  |  |  |  |  |
| --- | --- | --- | --- | --- | --- | --- | --- | --- | --- | --- |
| Groups |  |  |  |  |  |  |  |  |  |  |
| n (total 704) |  |  |  |  |  |  |  |  |  |  |
| **p values in Sant Pau cohort** | | | | | | | | | | |
|  | HC vs. ALS | HC vs. ALS-FTD | HC vs. bvFTD | HC vs. PSP | ALS vs. ALS-FTD | ALS vs. bvFTD | ALS vs. PSP | ALS-FTD vs. bvFTD | ALS-FTD vs. PSP | bvFTD vs. PSP |
| Age (mean ± SD) | *p* = 0.029 | - | - | - | - | - | - | - | - | - |
| Aβ1-42 (pg/ml)(median[IQR]) | - | - | *p* = 0.001 | - | - | - | - | - | - | - |
| Aβ42/40 (mean ± SD) | - | - | *p* = 0.003 | - | - | - | - | - | - | - |
| tTau (pg/ml)(median[IQR]) | - | - | *p* = 0.001 | *p* = 0.026 | - |  | - | - | - | - |
| pTau (pg/ml)(median[IQR]) | - | - | *p* = 0.021 | *p* = 0.019 | - |  | - | - | - | - |
| MMSE (mean ± SD) | - | *p* = 0.02 | *p* = 0.032 | *p* = 0.014 | - |  | - | - | - | - |
| NPI-Q (mean ± SD) | *-* | *-* | *-* | *-* | - | *-* | - | *p* = 0.043 | - | - |
| CDR-SB (mean ± SD) | - | - | - | - | - | - | - | - | - | - |
| CDR plus NACC FTLD (mean ± SD) | *-* | *-* | *-* | *-* | - | *-* | - | *p* = 0.021 | - | - |
| PSP-RS (mean ± SD) |  |  |  |  |  |  |  |  |  |  |
| UPDRS III (mean ± SD) |  |  |  |  |  |  |  |  |  |  |
| FAB (mean ± SD) |  |  |  |  |  |  |  |  |  |  |
| ALS specific(mean ± SD) |  |  |  |  |  |  |  |  |  |  |
| ALS-nonspecific (mean ± SD) |  |  |  |  |  |  |  |  |  |  |
| ECAS Total (mean ± SD) |  |  |  |  |  |  |  |  |  |  |
| ALS FRS-R (mean ± SD) |  |  |  |  |  |  |  |  |  |  |
| Disease duration (months) (median [IQR]) | - | - | - | - | - | - | - | - | - | - |
| Plasma Nfl (pg/mL)(median[IQR]) | *-* | - | *p* = 0.0014 | *p* = 0.0098 | - | - | - | - | - | - |
| sEV 3R Tau (pg/mL)(median[IQR]) | - | - | *-* | *p* = 0.0026 | - | - | *p* = 0.0003 | - | *p* = 0.0003 | *p* = 0.000024 |
| sEV 4R Tau (pg/mL)(median[IQR]) | - | - | *p* = 0.001 | *p* = 0.003 | - | - | - | - | - | *p* = 0.000047 |
| mEV 3R Tau (pg/mL)(median[IQR]) | - | - | *p* = 0.023 | *p* = 0.0041 | - | - | - | - | - | *p* = 0.0000087 |
| mEV 4R Tau (pg/mL)(median[IQR]) | - | - | *p* = 0.0014 | *p* = 0.023 | - | - | - | - | - | *p* = 0.0000011 |
| sEV 3R/4R Tau ratio(median[IQR]) | - | - | *p* = 0.000009 | *p* = 0.000007 | - | *p* = 0.0000091 | *p* = 0.0000016 | *p* = 0.0000074 | *p* = 0.0000041 | *p* = 0.000006 |
| mEV 3R/4R Tau ratio(median[IQR]) | - | - | p = 0.0000056 | *p* = 0.0000027 | - | *p* = 0.0000067 | *p* = 0.0000018 | *p* = 0.0000061 | *p* = 0.0000039 | *p* = 0.0000057 |
| sEV TDP-43 (pg/mL)(median[IQR]) | *p* = 0.0000075 | *p* = 0.0000046 | *p* = 0.00074 | *p* = 0.578 | - | *p* = 0.000009 | *p* = 0.000007 | *p* = 0.0000043 | *p* = 0.0000055 | *p* = 0.0005 |
| mEV TDP-43 (pg/mL)(median[IQR]) | *p*  = 0.0000014 | *p* = 0.0000033 | *p* = 0.00059 | *p* = 0.763 | - | *p* = 0.000005 | *p* = 0.0000062 | *p* = 0.0000053 | *p* = 0.0000042 | *p* = 0.00054 |
| EV particles (x10^7^/mL) | - | - | - | - | - | - | - | - | - | - |
